# Efficient, Regioselective Design of Mixed Cellulose Esters and Macroinitiators

**DOI:** 10.1021/acs.biomac.5c00432

**Published:** 2025-08-21

**Authors:** Jeffrey E. Thompson, Kevin J. Edgar

**Affiliations:** † Macromolecules Innovation Institute, 1757 Virginia Tech, Blacksburg, Virginia 24061, United States; ‡ Department of Sustainable Biomaterials, Virginia Tech, Blacksburg, Virginia 24061, United States

## Abstract

Mixed cellulose esters and macroinitiators with high C6-OH regioselectivity were synthesized by employing a tritylation protecting strategy, using sequential one-pot protection/acylation and deprotection/reacylation transformations, reducing the number of overall discrete steps and product isolations by half. This method produced organosoluble 2,3-di-*O*-acyl-6-*O*-(4-monomethoxytrityl) (2,3A-6MeOTr) cellulose intermediates, simplifying the generation of regioselectively substituted cellulose esters with controllable degree of substitution (DS) and substitution position. Treatment of 2,3A-6MeOTr celluloses with carboxylic acid anhydrides and trifluoroacetic acid (TFA) or acyl halides resulted in one-pot detritylation and reacylation of protected C6-OH groups without acyl migration or significant backbone degradation. Thermal analysis indicated that both DS and C6 compositions affected the glass transition temperature (*T*_g_) of mixed cellulose esters, highlighting the value of this method for structure–property relationship elucidation. Notably, mixed 2,3-di-*O*-A-6-*O*-B (2,3A-6B) cellulose esters with DS­(A) > 2.69 were semicrystalline. This method permits the facile generation of regioselectively substituted 2,3A-6B cellulose esters, including well-defined macroinitiators for controlled radical polymerization.

## Graphical abstract

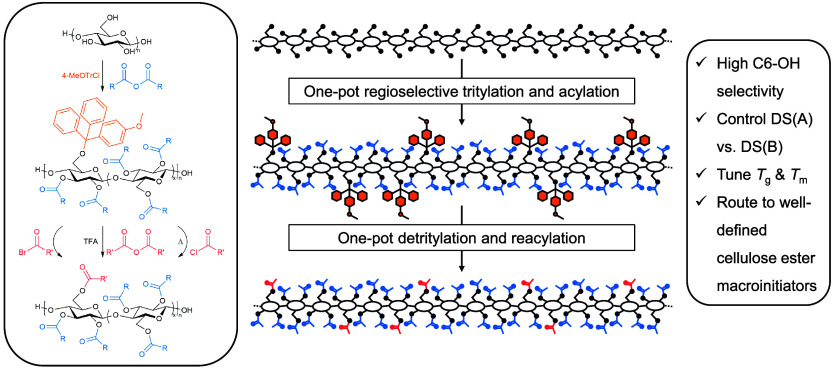

## 1 Introduction

With increasing concerns over growing plastic pollution and depletion of nonrenewable fossil fuels for energy and material production, there is great interest in developing sustainable, high-performance materials from renewable feedstocks. Polysaccharides are a diverse class of abundant, renewable, and biodegradable materials, and their derivatives have found commercial significance in applications such as biodegradable packaging, surfactants, drug delivery formulations, and optical displays. Cellulose, a linear homopolymer comprising d-glucopyranose repeating units linked by β-1→4 glycosidic bonds, is biosynthesized annually in quantities estimated at billions of metric tons and is one of the most plentiful natural polymers on Earth. As such, cellulose and its derivatives, including cellulose esters, are appealing candidates as sustainable materials for applications including packaging and medicine due to their high abundance, generally low toxicity, and plentiful hydroxy groups which can be modified to yield materials with unique properties.^1^

Physicochemical properties of cellulose esters (and other polysaccharide derivatives), such as solubility,^2^ crystallinity,^3,4^ optical behavior,^5,6^ thermal properties,^7^ enzymatic degradability,^8^ and performance in drug delivery formulations^9^ depend strongly on type, position, and degree of substitution (DS). One consequence of this fact is that the synthesis of regioselectively substituted cellulose esters is required to fully illuminate structure–property relationships. However, regioselective modification of cellulose is challenging due to the minor reactivity differences between each anhydroglucose unit (AGU) hydroxy group (at the 2-, 3-, and 6-positions), and their overall poor reactivity, due to limited approach angles and slow macromolecular diffusion, thereby requiring harsh reaction conditions that are not conducive to selectivity. There are few effective routes to regioselectively substituted cellulose esters, and many require protecting group strategies that utilize the increased steric accessibility of primary C6 hydroxy groups compared to more hindered secondary C2/C3-OH.^10^ Such regioselective methods for C6 modification include bromination with *N*-bromosuccinimide (NBS)/triphenylphosphine (PPh_3_) in *N*,*N*-dimethylacetamide (DMAc)/LiBr,^11^ chlorination with methanesulfonyl chloride in *N*,*N*-dimethylformamide (DMF),^12^ or silylation with thexyldimethylsilyl chloride in *N*-methylpyrrolidone/ammonia.^13^

The steric demand of the triphenylmethyl (trityl) group makes it useful in regioselective polysaccharide modification, particularly for selective C6 etherification. Although tritylation has long been employed to determine primary hydroxy group content in cellulose derivatives,^14^ the work of Heinze^15^ and Klemm^16^ at the University of Jena pioneered the use of trityl ethers as protecting groups to synthesize cellulose derivatives with well-defined microstructures. Tritylation has been employed to generate regioselectively substituted cellulose ethers,^15,16^ esters,^17^ graft copolymers,^18,19^ and Janus bottlebrushes.^20,21^ The typical procedure for regioselective synthesis of cellulose derivatives incorporating trityl (or methoxytrityl, which is more reactive than trityl due to increased stability of the carbocation intermediate, enabling milder conditions and higher protection selectivity) protecting groups follows the motif of separate C6 protection, C2/C3 modification, C6 deprotection, and C6 modification operations. While effective at generating cellulose derivatives with high regioselectivity, this method requires four separate isolation steps. It is also notable that this sequence requires high DS­(trityl) to afford organosoluble 6-*O*-(methoxy)­trityl cellulose intermediates.^16^ As we shall describe, in some cases, it is desirable but difficult using existing procedures to prepare regioselectively substituted derivatives with predictably low DS of the C6 substituent. Due to these limitations, we hypothesized that it would be possible to simplify and broaden the impact of the synthesis of regioselectively substituted cellulose esters via tritylation using successive one-pot, two-step chemical modifications.

Trityl ethers are formed under basic conditions and are removed under acidic conditions. For cellulose esters, tritylation is typically performed homogeneously in DMAc/LiCl with pyridine base (to consume coproduct HCl), while deprotecting trityl ethers employs a strong acid (e.g., HCl) in a polar aprotic solvent, such as tetrahydrofuran (THF). Since cellulose esters can be formed under both acidic^22^ and basic^23^ conditions in similar solvents, we asked the following question: can we conduct both tritylation and acylation in a one-pot operation? Then, can we conduct detritylation and acylation in a separate one-pot operation without compromising regioselectivity for the overall four steps but with only two reaction vessels and two isolations? Achieving such synthetic transformations would greatly simplify the synthesis of 2,3-di-*O*-A-6-*O*-B (2,3A-6B) cellulose esters for subsequent structure–property relationship studies by reducing the number of overall transformations in half compared to the sequential four-step approach. Additionally, the expected good organic solubility of the 6-trityl (or methoxytrityl) cellulose ester products from the first two-step, one-pot sequence should enable the generation of organosoluble regioselectively substituted cellulose esters with control over DS of the C6 position, by permitting control of DS­(MeOTr) vs DS­(ester) at C6. This can drastically expand the range of compositions attainable when targeting a low DS of the C6 position, as a moderate DS­(trityl) of 0.41 is required for solubility in DMF.^16^

This heightened DS and compositional control at C6 could be particularly valuable for imparting functionality that can serve as precursors to well-defined polysaccharide graft (co)­polymers. As this regioselective modification is selective for C6-OH, the incorporation of either a site for subsequent polymerization (i.e., grafting-from) or coupling to a preformed polymer (i.e., grafting to) exclusively at the C6 position ensures a maximum of one branch point per repeating unit, offering distinct control over graft density. Such materials already have been found to be useful as compatibilizers for sustainable polymer blends^24,25^ and biobased thermoplastic elastomers.^26,27^ In some cases, control of graft density has a powerful impact upon blend compatibilization, with low graft density often being essential to mitigate phase separation and enhance compatibility in immiscible blends.^28^ Additionally, polysaccharide derivatives grafted with stimuli-responsive side chains including poly­(2-(dimethylamino)­ethyl methacrylate)^29^ or poly­(2-(diisopropyl)­ethyl methacrylate)^30^ can form pH-responsive micelles capable of encapsulating hydrophobic drugs with hydrodynamic radii and critical micelle concentrations that depend on graft length and graft density. Coupled with the extensive use of cellulose esters in drug delivery formulations,^9^ well-defined graft copolymers prepared from regioselectively substituted cellulose esters could be useful in improving the bioavailability of valuable drugs.

The goal of the present study is to develop an efficient synthetic route to regioselectively substituted 2,3A-6B cellulose esters through a four-step, two-pot process (Scheme 1). We hypothesize that 2,3-di-*O*-acyl-6-*O*-(4-monomethoxytrityl) (2,3A-6MeOTr) cellulose esters with varying DS­(A) and DS­(MeOTr) can be prepared in a one-pot manner by varying the reaction conditions. Additionally, we hypothesize that the resulting 2,3A-6MeOTr cellulose esters can be converted to 2,3A-6B cellulose esters in a one-pot manner through treatment with an acyl donor (such as a carboxylic acid anhydride or acyl halide) under acidic conditions while maintaining regioselectivity. Development of such a procedure will be beneficial for future generation of regioselectively substituted 2,3A-6B cellulose esters to elucidate structure–property relationships, as well as more complicated polysaccharide derivatives including well-defined graft copolymers. The new derivatives of sustainable cellulose accessible in this way will serve as access points to biodegradable plastics, drug delivery vehicles, compatibilizers, and other useful materials.

**Scheme 1:**
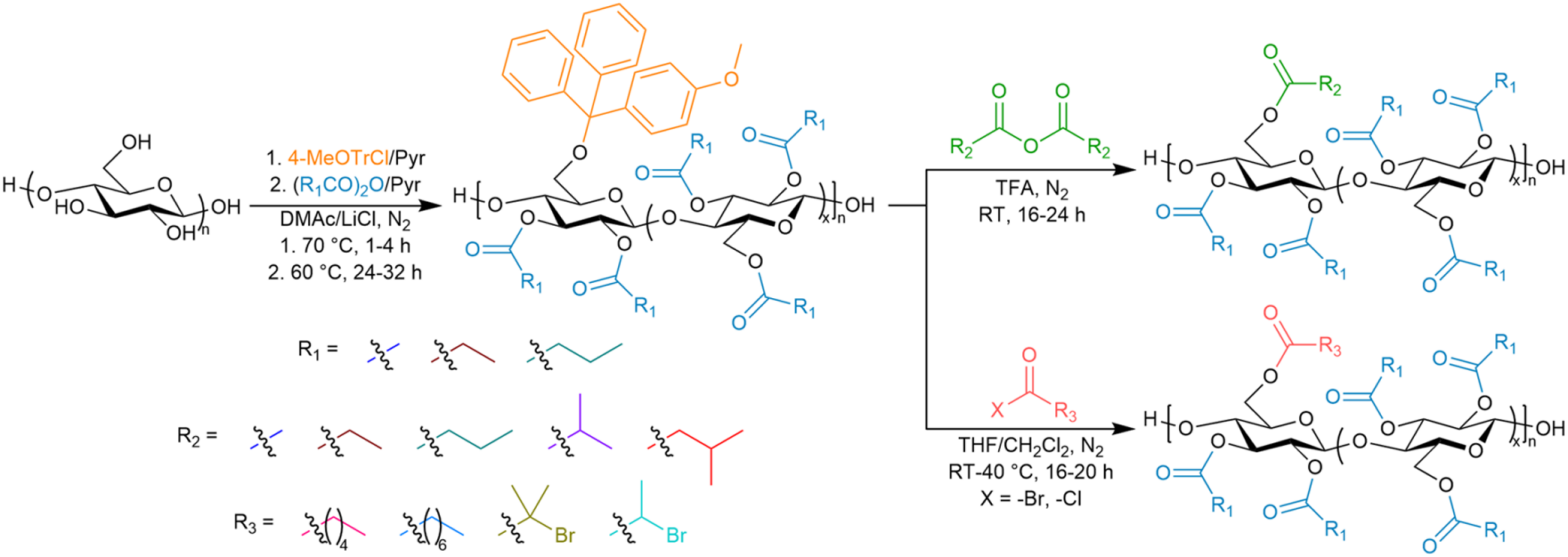
Synthetic Route to 2,3A-6B Cellulose Esters with DS and Position Control, Resulting in a Random Copolymer Comprising 2,3-Di-*O*-A-6-*O*-B and 2,3,6-Tri-*O*-A Monosaccharides

## 2 Experimental Section

### 2.1 Materials

Microcrystalline cellulose (MCC, Avicel PH-101, Fluka, DP*_n_* = 182, *Đ* = 1.81 as determined by SEC of its carbanilate)^31^ was dried overnight at 50 °C under reduced pressure before use. LiCl (Acros) was dried overnight at 120 °C under reduced pressure and stored in a desiccator prior to use. Dichloromethane (DCM, Fisher) was distilled from CaH_2_ (Acros) onto 3Å molecular sieves and stored under dry N_2_ until use. THF (Fisher) was stored over 3Å molecular sieves under dry N_2_ until use. DMAc (Fisher) and pyridine (Sigma) were stored over 4Å molecular sieves under dry N_2_ until use. 4-Monomethoxytrityl chloride (4-MeOTrCl, Oakwood), acetic anhydride (Ac_2_O, Sigma), propionic anhydride (Pr_2_O, Thermo Scientific), butyric anhydride (Bu_2_O, Sigma), isobutyric anhydride (*i*Bu_2_O, Thermo Scientific), isovaleric anhydride (*i*Va_2_O, TCI), trifluoroacetic acid (TFA, Sigma), α-bromoisobutyryl bromide (B*i*BBr, TCI), α-bromopropionyl bromide (BPrBr, Oakwood), hexanoyl chloride (HexCl, TCI), octanoyl chloride (OctCl, Sigma), 4-dimethylaminopyridine (DMAP, Sigma), and phenyl isocyanate (PhNCO, Sigma) were used as received. Regenerated cellulose dialysis tubing (1 kDa MWCO, Spectrum) was soaked in deionized H_2_O before use. All other solvents and materials were of reagent grade and used as received.

### 2.2 Measurements

NMR spectra (^1^H, ^13^C, quantitative ^13^C (q^13^C), ^1^H–^1^H correlation (COSY), ^1^H–^13^C heteronuclear single quantum coherence (HSQC), and ^1^H–^13^C heteronuclear multiple bond correlation (HMBC)) were obtained on either an Agilent MR4DD2 400 MHz spectrometer with a broadband OneNMR probe, a Bruker Avance II 500 MHz spectrometer equipped with a BBO Prodigy probe, or a Bruker Avance III 600 MHz spectrometer equipped with a TCI Prodigy probe, all conducted at room temperature (RT). All spectra were referenced to residual solvent resonances of either deuterated dimethyl sulfoxide (DMSO-*d*_6_) or deuterated chloroform (CDCl_3_). Samples were dissolved at approximately 5 mg/mL for ^1^H NMR measurements using the 400 MHz spectrometer to determine DS­(MeOTr), DS­(Ac), and DS­(X), where X is either an acetyl, propionyl, butyryl, isobutyryl, isovaleryl, hexanoyl, octanoyl, or bromopropionyl moiety. Samples were dissolved at approximately 80 mg/mL for ^1^H/^13^C/q^13^C and COSY/HSQC/HMBC using either the 500 or 600 MHz spectrometer, respectively. ^1^H NMR spectra were collected using at least 64 scans with a 1 s delay. ^13^C NMR spectra were collected using at least 2048 scans with 3 s delay. Samples for q^13^C NMR contained 25 mM chromium­(III) acetylacetonate (Cr­(Acac)_3_), and q^13^C spectra were obtained using at least 3072 scans with a 2.5 s delay. COSY (at least 4 scans, 1 s delay), HSQC (at least 16 scans, 1.5 s delay), and HMBC (at least 32 scans, 2 s delay) spectra were obtained using 512 increments. Fourier transform infrared (FTIR) spectra were collected on a Varian 670 IR spectrometer equipped with a Pike Technologies GladiATR attachment and obtained as the average of 32 scans. Thermal stability was evaluated via thermogravimetric analysis (TGA) using a TA Instruments TGA 550 instrument with a heating rate of 20 °C/min up to 500 °C in a N_2_ atmosphere. Thermal transitions were determined by differential scanning calorimetry (DSC) using a TA Instruments DSC Q2500. Approximately 3–7 mg of sample in aluminum pans was equilibrated at 25 °C then heated to approximately 25 °C below onset of decomposition (as obtained from TGA) at 10 °C/min, then cooled to 0 °C at 20 °C/min, then heated again at 10 °C/min to approximately 25 °C below onset of decomposition. The glass transition (*T*_g_) and melting (*T*_m_) temperatures were obtained from the second heating scan. The *T*_g_ was reported as the midpoint of the endothermic step transition, while the *T*_m_ was reported as the maximum of the melting endotherm, both an average of 3 runs. Size exclusion chromatography with multiangle light scattering (SEC-MALS) was performed using DMAc with 50 mM LiCl as the mobile phase at 40 °C at a flow rate of 1.0 mL/min on two Agilent Technologies PLgel 10 μm MIXED-B LS 300 × 7.5 mm^2^ columns connected in series. Detection consisted of a Wyatt Technology TRIOS II light scattering detector and Optilab T-REX differential refractive (dRI) detector. Molecular weights and dispersities were calculated using Wyatt ASTRA software and off-line d*n*/d*c* analysis, assuming 100% mass recovery. Absolute molecular weights and dispersities were calculated using Wyatt ASTRA software and d*n*/d*c* analysis, assuming 100% mass recovery. DS values were obtained by either ^1^H or q^13^C NMR spectroscopy (see the Supporting Information for calculation formulas).

### 2.3 Representative Procedure for the Synthesis of 2,3-Di-*O*-acetyl-6-*O*-(4-monomethoxytrityl) (2,3Ac-6MeOTr) Cellulose

Dissolution of MCC in DMAc/LiCl was adapted from an existing literature procedure.^32^ MCC (1.00 g, 6.17 mmol AGU) was slurried in anhydrous DMAc (15 mL) and heated to 150 °C under N_2_ over 30 min in a 3-neck flask equipped with a mechanical stirrer, N_2_ inlet, and short-path distillation apparatus. Dry LiCl (1.50 g, 35.4 mmol, 5.74 equiv/AGU) and additional anhydrous DMAc (10 mL) were quickly added to the flask; the temperature was raised to 180 °C, and DMAc (10 mL) was distilled under N_2_ to remove adventitious water present. The flask was cooled to RT under N_2_, with a transparent, viscous, amber solution resulting within 2 h. Anhydrous pyridine (2.24 mL, 27.8 mmol, 4.50 equiv/AGU) was added slowly to the flask and mixed for 10 min, followed by 4-MeOTrCl (0.92 g, 3.08 mmol, 0.50 equiv/AGU) and additional anhydrous DMAc (9 mL), which was mixed for 20 min, all at RT under N_2_. The flask was then lowered into an oil bath equilibrated at 70 °C and allowed to stir for 4 h. The oil bath temperature was then reduced to 60 °C over 20 min, after which time additional anhydrous pyridine (9.94 mL, 123.4 mmol, 20.0 equiv/AGU) was added quickly and Ac_2_O (8.75 mL, 92.5 mmol, 15.0 equiv/AGU) was added dropwise to the flask. The solution was stirred for an additional 24 h, after which time it was cooled to RT and added slowly to methanol (MeOH, 700 mL) to precipitate the product. The product was isolated by filtration, twice redissolved in CHCl_3_ (25 mL), reprecipitated in ethanol (EtOH, 500 mL), and then washed with hexanes. The final product was collected, air-dried, and then dried under reduced pressure at 50 °C overnight. If unremoved byproducts were still present in ^1^H NMR spectra, the product was washed further via Soxhlet extraction with 1:1 EtOH/diethyl ether (Et_2_O) for 24 h and then collected and dried under reduced pressure at 50 °C overnight. Entry E-MeOTr, Table 1. Yield: 1.91 g (70%). ^1^H NMR (500 MHz, CDCl_3_): 1.68 (C3–O–COCH_3_ vicinal to C2–O–MeOTr), 1.94 (C3–O–COCH_3_), 2.01 (C2–O–COCH_3_), 2.13 (C6–O–COCH_3_), 3.08–5.07 (H1–H5), 3.81 (–C­(C_6_H_4_OCH_3_)­(C_6_H_5_)_2_), 4.06–4.37 (C6–CH_2_–O–COCH_3_), 4.09–4.32 (C6–CH_2_-O-MeOTr), 6.71–7.49 (–C­(C_6_ H_4_OCH_3_)­(C_6_ H_5_)_2_). ^1^H NMR (400 MHz, DMSO-*d*_6_): 1.56 (C3–O–COCH_3_ vicinal to C2–O–MeOTr), 1.88 (C3–O–COCH_3_), 1.94 (C2–O–COCH_3_), 2.07 (C6–O–COCH_3_), 3.47–5.21 (H1–H6/6′), 3.73 (–C­(C_6_H_4_OCH_3_)­(C_6_H_5_)_2_), 6.77–6.95 (–C­(C_6_ H_4_OCH_3_)­(C_6_H_5_)_2_), 7.25–7.59 (–C­(C_6_H_4_OCH_3_)­(C_6_ H_5_)_2_). ^13^C NMR (125 MHz, CDCl_3_): 20.6 (C2–O–COCH_3_), 20.7 (C3–O–COCH_3_), 21.0 (C6–O–COCH_3_), 55.4 (–Ph–OCH_3_), 60.1 (C6–CH_2_–O–MeOTr), 62.1 (C6–CH_2_–O–COCH_3_), 71.8–76.1 (C2–C5), 86.5 (–C­(C_6_H_4_OCH_3_)­(C_6_H_5_)_2_), 97.8 (C1–CH–C2–O–MeOTr), 100.3 (C1–CH–C2–O–COCH_3_), 113.6 (Ph–CH–C–OCH_3_), 127.3–143.7 (Ph–CH), 159.1 (Ph–CH=C–OCH_3_), 169.4 (C6–O–COCH_3_), 169.9–170.4 (C2/C3–O–COCH_3_).

### 2.4 Representative Procedure for the Synthesis of 2,3-Di-*O*-acetyl-6-*O*-propionyl (2,3Ac-6Pr) Cellulose Using TFA/Pr_2_O

In an oven-dried, N_2_-flushed 2-dram vial equipped with a magnetic stir bar and rubber septum, 2,3Ac-6MeOTr cellulose (100 mg, 0.28 mmol AGU, DS­(Ac) 2.69, DS­(MeOTr) 0.31) was dissolved in Pr_2_O (3.58 mL, 27.8 mmol, 99.3 eq/AGU) at RT under dry N_2_. TFA (0.42 mL, 5.60 mmol, 20.0 equiv/AGU) was then added dropwise to the solution under dry N_2_. The solution quickly turned red and was allowed to stir overnight (16 h) at RT (for samples A- and B-Pr, 40 and 25 equiv TFA/AGU were employed, respectively, and the solution was stirred for 24 h at RT). The solution was then added dropwise to 80 mL 1:1 EtOH/hexanes to precipitate the product. The solid was isolated by filtration, then redissolved in minimal DCM, and reprecipitated in 1:1 EtOH/hexanes (80 mL). The product was then isolated by filtration, then further washed and isolated by centrifugation with EtOH (2 × 9000 rpm, 30 min, RT). The product was further rinsed with EtOH, collected, air-dried, and then dried under reduced pressure at 50 °C. Entry F-Pr, Table 2. Yield: 53.1 mg (68%). ^1^H NMR (600 MHz, DMSO-*d*_6_): 0.94 (C2–O–COCH_2_CH_3_), 1.05 (C6–O–COCH_2_CH_3_), 1.87–1.94 (C2/C3–O–COCH_3_), 2.07 (C6–O–COCH_3_), 2.26–2.45 (Pr–O–COCH_2_CH_3_), 3.66–5.08 (H1–H5), 4.00–4.23 (H6/H6′). ^1^H NMR (600 MHz, CDCl_3_): 1.04 (C2–O–COCH_2_CH_3_), 1.16 (C6–O–COCH_2_CH_3_), 1.92 (C3–O–COCH_3_), 1.99 (C2–O–COCH_3_), 2.11 (C6–O–COCH_3_), 2.18 (C2–O–COCH_2_CH_3_), 2.37 (C6–O–COCH_2_CH_3_), 3.51 (H5), 4.03 (H6′), 4.38 (H6), 4.39 (H1), 4.77 (H2), 5.04 (H3). ^13^C NMR (150 MHz, DMSO-*d*_6_): 8.8–8.9 (C2/C6–O–COCH_2_ CH_3_), 20.1–20.2 (C2/C3–O–COCH_3_), 20.6 (C6–O–COCH_3_), 26.8–26.9 (C2/C6–O–COCH_2_CH_3_), 62.6 (C6), 71.3–76.1 (C2–C5), 99.3 (C1), 169.1 (C6–O–COCH_3_), 169.4–170.4 (C2/C3–O–COCH_3_), 173.5 (C6–O–COCH_2_CH_3_). ^13^C NMR (150 MHz, CDCl_3_): 9.1 (C2–O–COCH_2_ CH_3_), 9.2 (C6–O–COCH_2_ CH_3_), 20.6 (C3–O–COCH_3_), 20.7 (C2–O–COCH_3_), 20.9 (C6–O–COCH_3_), 27.4 (C2–O–COCH_2_CH_3_), 27.5 (C6–O–COCH_2_CH_3_), 62.1 (C6), 71.8 (C2), 72.6 (C3), 72.8 (C5), 76.1 (C4), 100.6 (C1), 169.4 (C2–O–COCH_3_), 169.9 (C3–O–COCH_3_), 170.3 (C6–O–COCH_3_), 173.3 (C2–O–COCH_2_CH_3_), 173.8 (C6–O–COCH_2_CH_3_).

### 2.5 Representative Procedure for the Synthesis of 2,3-Di-*O*-acetyl-6-*O*-bromoisobutyryl (2,3Ac-6B*i*B) Cellulose Using B*i*BBr

In an oven-dried, N_2_-flushed 50 mL single-neck flask equipped with a magnetic stir bar and rubber septum, 2,3Ac-6MeOTr cellulose (500 mg, 1.63 mmol of AGU, DS­(Ac) 2.88, DS­(MeOTr) 0.12) was dissolved in dry DCM (20 mL) under N_2_. Then, B*i*BBr (0.61 mL, 4.89 mmol, 3 eq/AGU) was added dropwise to the flask under N_2_ and the solution was stirred overnight (16 h) at RT. The solution was then added dropwise to 400 mL of isopropanol (IPA) to precipitate the product, which was isolated by filtration, redissolved in minimal DCM, and reprecipitated in EtOH (400 mL). The product was then filtered off and rinsed with EtOH and hexanes, collected, air-dried, and then dried under reduced pressure at 50 °C overnight. Entry G-B*i*B, Table 4. Yield: 437 mg (93%). ^1^H NMR (600 MHz, CDCl_3_): 1.92 (C3–O–COCH_3_), 1.95 (C6–O–COCBr­(CH_3_)_2_), 1.99 (C2–O–COCH_3_), 2.11 (C6–O–COCH_3_), 3.46–5.13 (H1–H5), 3.67–3.77 (C6–CH_2_–O–COCBr­(CH_3_)_2_), 4.04–4.35 (C6–CH_2_–O–COCH_3_). ^13^C NMR (150 MHz, CDCl_3_): 20.4–20.6 (C2/C3–O–COCH_3_), 20.8 (C6–O–COCH_3_), 30.6 (C6–O–COCBr­(CH_3_)_2_), 55.8 (C6–O–COCBr­(CH_3_)_2_), 59.9 (C6–CH_2_–O-COCBr­(CH_3_)_2_), 61.9 (C6–CH_2_–O–COCH_3_), 71.6–76.0 (C2–C5), 100.5 (C1), 169.3 (C6–O–COCH_3_) 169.7–170.2 (C2/C3–O–COCH_3_), 170.8 (C6–O–CO­(CH_3_)_2_Br).

Additional synthetic procedures and spectroscopic assignments are provided in the Supporting Information.

## 3 Results and Discussion

### 3.1 One-Pot Regioselective Synthesis of 2,3A-6MeOTr Cellulose

We first sought to synthesize 2,3-di-*O*-acetyl-6-*O*-(4-monomethoxytrityl) (2,3Ac-6MeOTr) cellulose in DMAc/LiCl through treatment of microcrystalline cellulose (MCC) with 4-monomethoxytrityl chloride (4-MeOTrCl) and pyridine by adapting an existing procedure resulting in 6-*O*-monomethoxytrityl cellulose with DS­(6-MeOTr) 0.97 and 94% selectivity for C6 tritylation.^16^ Subsequent addition of excess pyridine and acetic anhydride (Ac_2_O) to the same vessel afforded persubstituted 2,3Ac-6MeOTr cellulose with DS­(Ac) 1.59 and DS­(MeOTr) 1.41 as determined by ^1^H NMR spectroscopy (Sample A-MeOTr, Table 1). In addition to the ^1^H NMR spectroscopic evidence, the lack of −OH stretching (centered at 3304 cm^–1^) and appearance of absorption bands at 704, 883 (aromatic methine bending), 1178 (methoxy ether bending), 1367 (acetyl methyl bending), 1510, 1606 (aromatic alkene stretching), and 1744–1754 cm^–1^ (ester carbonyl stretching) observed in the FTIR spectrum were consistent with full hydroxy substitution with either acetyl or methoxytrityl moieties (Figure S1). In the ^1^H NMR spectrum, we observed the characteristic resonances of the 2- and 3-*O*-acetyl methyl groups at 2.01 and 1.94 ppm, respectively, but also observed a resonance at 1.68 ppm which correlates (HMBC in CDCl_3_ (Figure S2)) with a carbonyl resonance at 169.1 ppm. We attribute this resonance to C3-*O*-acetyl methyl groups vicinal to C2-*O*-MeOTr moieties, with the chemical shift upfield being attributed to aromatic ring currents from the nearby methoxytrityl moiety. A similar effect was observed for regioselectively substituted cellulose benzoate propionates, where resonances of propionate esters vicinal to benzoates appeared upfield of other propionate ester resonances in ^1^H NMR.^5^

HSQC NMR indicated two distinct correlations in the anomeric region at 97.8 and 100.3 ppm, suggesting that some monosaccharide repeating units differed in the composition at the C2 position (Figure S3). This is in agreement with previous observations where commercially available cellulose acetate (CA) (i.e., cellulose acetate with DS­(Ac) 1.8, Eastman CA320S) exhibits two distinct anomeric resonances associated with AGUs containing either an acetylated or unsubstituted hydroxy group at C2.^12^ This information, coupled with DS calculations (DS­(MeOTr) 1.41), indicated that there was significant tritylation of the C2 position for sample A-MeOTr, which was a result of the greater acidity of the C2-OH. This is consistent with previous studies that indicate that tritylation at the C2-OH competes with C6-OH at higher amounts of tritylating reagent, although the addition of excess pyridine prior to adding Ac_2_O (intended to keep the reaction medium basic to prevent MeOTr cleavage) may explain the significant C2-OH protection compared to previous studies.^16^ A single resonance at 60.7 ppm in the ^13^C NMR spectrum of sample A-MeOTr was attributed to the tritylated C6-OH, suggesting that nearly all of the C6 positions were protected (Figure S4).

As previously mentioned, cellulose derivatives with DS at C6 much less than one can be valuable targets, enabling the synthesis of useful materials like effective blend compatibilizers, thermoplastic elastomers, and stimuli-responsive micelles. Access to such polymers in a regioselective fashion is highly desirable, making possible more enlightening and precise structure–property investigations. However, the synthesis of such derivatives through C6 trityl protection is greatly hampered by the poor solubility of cellulose containing a lower DS of C6 trityl groups. Therefore, we sought to build on our initial results to achieve a 2,3Ac-6MeOTr cellulose ester with DS­(6-MeOTr) of much less than 1.00 with good solubility in common organic solvents. We previously demonstrated control over DS­(C6) by one-pot synthesis of 2,3-di-*O*-acetyl-6-bromo-6-deoxy amylose; this was achieved by controlling the stoichiometry of the C6-targeting reagent (in that case, NBS/PPh_3_) and duration during which only the C6-OH targeting reagent was present (in other words, the delay before adding acylating reagent).^33^ In the current work, similar tactics proved amenable to the synthesis of 2,3Ac-6MeOTr cellulose with DS­(MeOTr) ranging from 0.01 to 0.96 achieved by varying stoichiometry and tritylation time (Table 1 and Figures S5–S9).

**Table 1:** Results of One-Pot Synthesis of 2,3Ac-6MeOTr Cellulose in DMAc/LiCl

sample	4-MeOTrCl:AGU (equiv)	tritylation time at 70 °C (h)	DS(MeOTr)	DS(Ac)
A-MeOTr	3:1	4	1.41	1.59
B-MeOTr	1:1	4	0.96	2.04
C-MeOTr	1:1	2	0.66	2.34
D-MeOTr	1:1	1	0.61	2.39
E-MeOTr	0.5:1	4	0.39	2.61
F-MeOTr	0.5:1	1	0.31	2.69
G-MeOTr	0.25:1	1	0.12	2.88
H-MeOTr	0.1:1	1	0.01	2.99

2,3-Di-*O*-propionyl-6-*O*-(4-monomethoxytrityl) (2,3Pr-6MeOTr) and 2,3-di-*O*-butyryl-6-*O*-(4-monomethoxytrityl) (2,3Bu-6MeOTr) celluloses were also prepared through this method by employing the corresponding acyl anhydride, achieving DS­(MeOTr) similar to that of 2,3Ac-6MeOTr sample prepared under analogous conditions (Figures 1 and S10–S16 and Table S1). However, DS calculations indicated that 2,3Pr-6MeOTr and 2,3Bu-6MeOTr cellulose were not fully substituted, potentially due to the increased steric hindrance of propionic (Pr_2_O) and butyric (Bu_2_O) anhydrides preventing complete reaction with cellulosic hydroxy groups. It is possible that extended reaction times may be required to peracylate cellulose with longer-chain aliphatic anhydrides. To the best of our knowledge, this is the first reported one-pot preparation of cellulose esters with C6-OH regioselectively protected by methoxytrityl ether groups.

**Figure 1:**
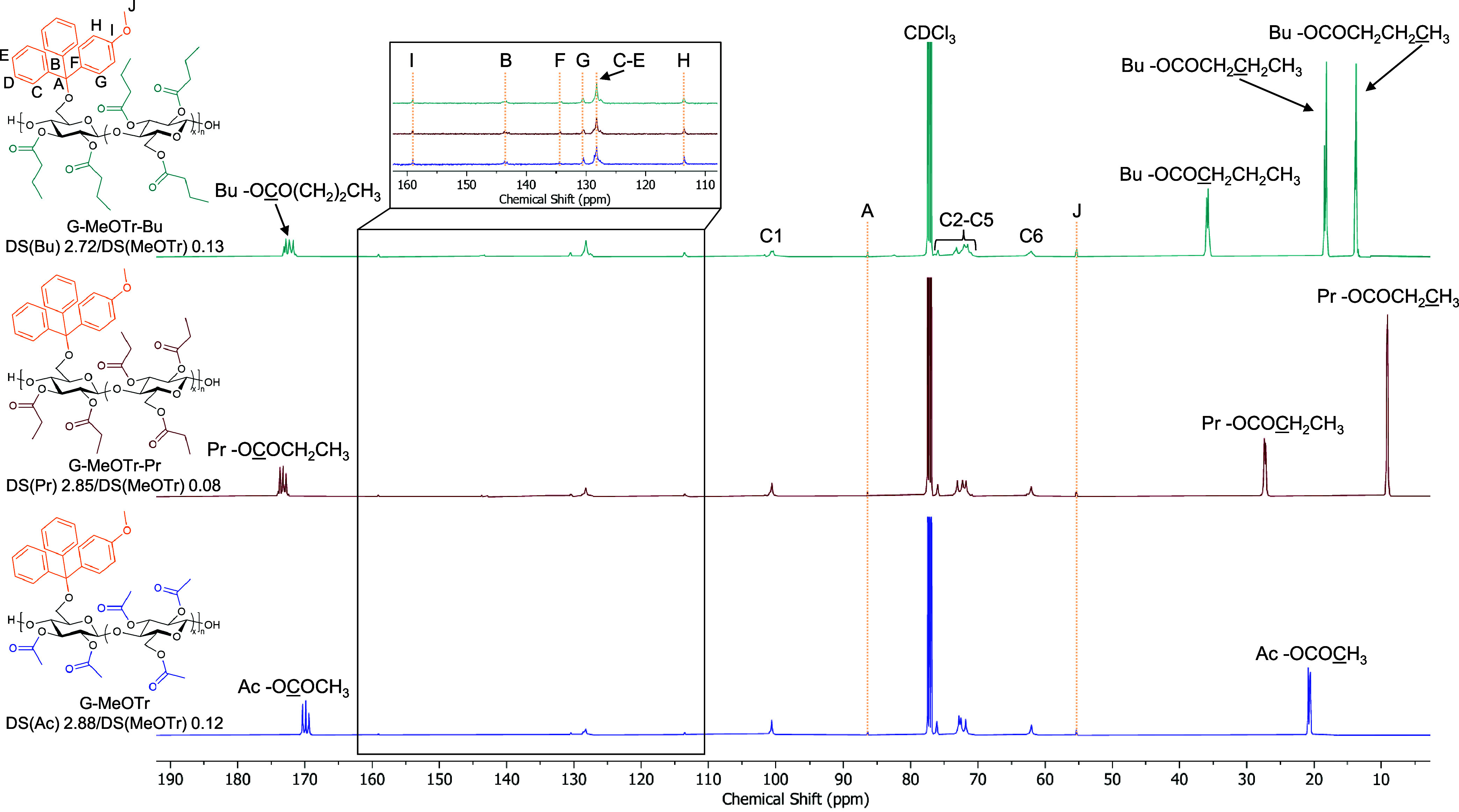
Stacked ^13^C NMR spectra of 2,3Ac-6MeOTr cellulose DS­(Ac) 2.88 (G-MeOTr), 2,3Pr-6MeOTr cellulose DS­(Pr) 2.85 (G-MeOTr-Pr), and 2,3Bu-6MeOTr cellulose DS­(Bu) 2.72 (G-MeOTr-Bu).

Notably, we observed no significant degree of polymerization (DP) loss during the conversion of MCC (DP*_n_* = 182) to 2,3Ac-6MeOTr cellulose (Table S2). This indicated that conditions for this efficient transformation were mild enough to prevent substantial hydrolysis of anomeric linkages. Interestingly, we observed an increase in DP_n_ for the derivatives analyzed. We suspect that this apparent increase in DP_n_ is due to the loss of low molecular weight (MW) fractions during the multiple precipitations employed for purification, as the synthesized 2,3Ac-6MeOTr cellulose derivatives were recovered in approximately 65–80% yields. For example, *M*_n_ for 2,3Ac-6MeOTr cellulose DS­(Ac) 1.59 (A-MeOTr) and 2.69 (F-MeOTr) was determined to be 141.3 and 70.9 kg/mol, respectively, corresponding to DP_n_ values of 231 and 197, respectively. Cellulose is known to undergo some main-chain scission during dissolution in DMAc/LiCl^34^ and is susceptible to both acidic and alkaline hydrolyses, all of which result in DP loss. An excess of pyridine was employed during the acylation-tritylation procedure (to neutralize both HCl and carboxylic acid generated during tritylation and acylation, respectively) to prevent backbone hydrolysis and unintended detritylation. However, the pyridinium hydrochloride and pyridinium alkanoate salts generated *in situ* are still weakly acidic and may cause some hydrolysis of glycosidic linkages.

### 3.2 One-Pot Synthesis of 2,3A-6B Cellulose Esters from 2,3A-6MeOTr Cellulose Employing TFA and Carboxylic Acid Anhydrides

Having established synthetic control with regard to the DS of acyl and MeOTr groups of these regioselectively substituted derivatives, we wished to quantify methoxytrityl substitution position to determine whether competing acylation affected tritylation regioselectivity, and to enable comparison with the conventional method, with its four separate steps and isolations.^17^ Surprisingly, there is a relative dearth in the literature of studies concerning the regioselective synthesis of mixed cellulose esters employing (methoxy)­trityl ethers as intermediate protecting groups, especially targeting DS­(6-ester) < 1 and DS­(2,3-ester) > 2.^8,17^ Conventionally, quantification of substitution position involves treating the tritylated cellulose derivative with a strong acid, such as HCl^16^ or HBr,^17^ to remove the trityl ether, isolating the deprotected cellulose ester, and then reacting newly liberated C6-OH with a new moiety that can easily be quantified by ^1^H NMR spectroscopy. Liebert, Hussain, and Heinze observed that propionylation of unsubstituted CA hydroxy groups with Pr_2_O facilitates quantification of CA substitution positions by ^1^H NMR spectroscopy; they observed no acyl migration during propionylation.^35^ However, we wished to achieve detritylation and propionylation of deprotected hydroxy groups in a one-pot manner and determine whether milder reaction conditions could be achieved for such a transformation.

Haloacetic acids, such as trifluoroacetic acid (TFA, p*K*_a_ = 0.23), are commonly used as detritylating agents in nucleoside chemistry^36^ and are significantly weaker acids than either HCl (calculated p*K*_a_ = −6.12) or HBr (calculated p*K*_a_ = −9.69).^37^ The lower acidity of TFA, paired with its organic solubility, piqued our interest as a milder detritylating agent compared with the mineral acids previously employed. Besides its use as a deprotecting agent, TFA (and its anhydride) have a long history as acylation catalysts in cellulose chemistry and have been employed to generate cellulose esters without incorporation of trifluoroacetyl moieties.^38^ Inspired by the “impeller method” pioneered by early cellulose chemists,^39^ we envisioned that treating 2,3Ac-6MeOTr cellulose with TFA and Pr_2_O could achieve simultaneous detritylation and propionylation. To our advantage, all 2,3Ac-6MeOTr cellulose esters prepared were soluble in Pr_2_O or dissolved after addition of TFA, permitting a transformation without the need for an inert diluent where Pr_2_O acted as both reaction medium and acylating agent. This is particularly useful as the solubility of the intermediate and excess of reagent (as solvent) allow for milder reaction conditions, reducing the likelihood of significant glycosidic linkage acidic hydrolysis.

During concurrent detritylation/propionylation, TFA serves two distinct roles: it will react stoichiometrically as a Brønsted-Lowry acid to remove the trityl ether, deprotecting the hydroxy group and generating the relatively stable trityl cation, and it will react catalytically as an “impelling agent” with Pr_2_O, as it will form the mixed trifluoroacetyl-propionyl anhydride *in situ*, which is the active acylating agent (Scheme 2). Since TFA has a lower p*K*_a_ than propionic acid (p*K*_a_ = 4.87), it is a more stable leaving group and results in preferential propionylation over trifluoroacetylation. TFA is regenerated after the mixed anhydride reacts with the liberated C6-OH, which can then react with either Pr_2_O or C6-MeOTr moieties still present in solution. We have not conducted an in-depth mechanistic analysis of this transformation, so it is possible that this is a concerted reaction, rather than sequential detritylation followed by acylation.

**Scheme 2:**
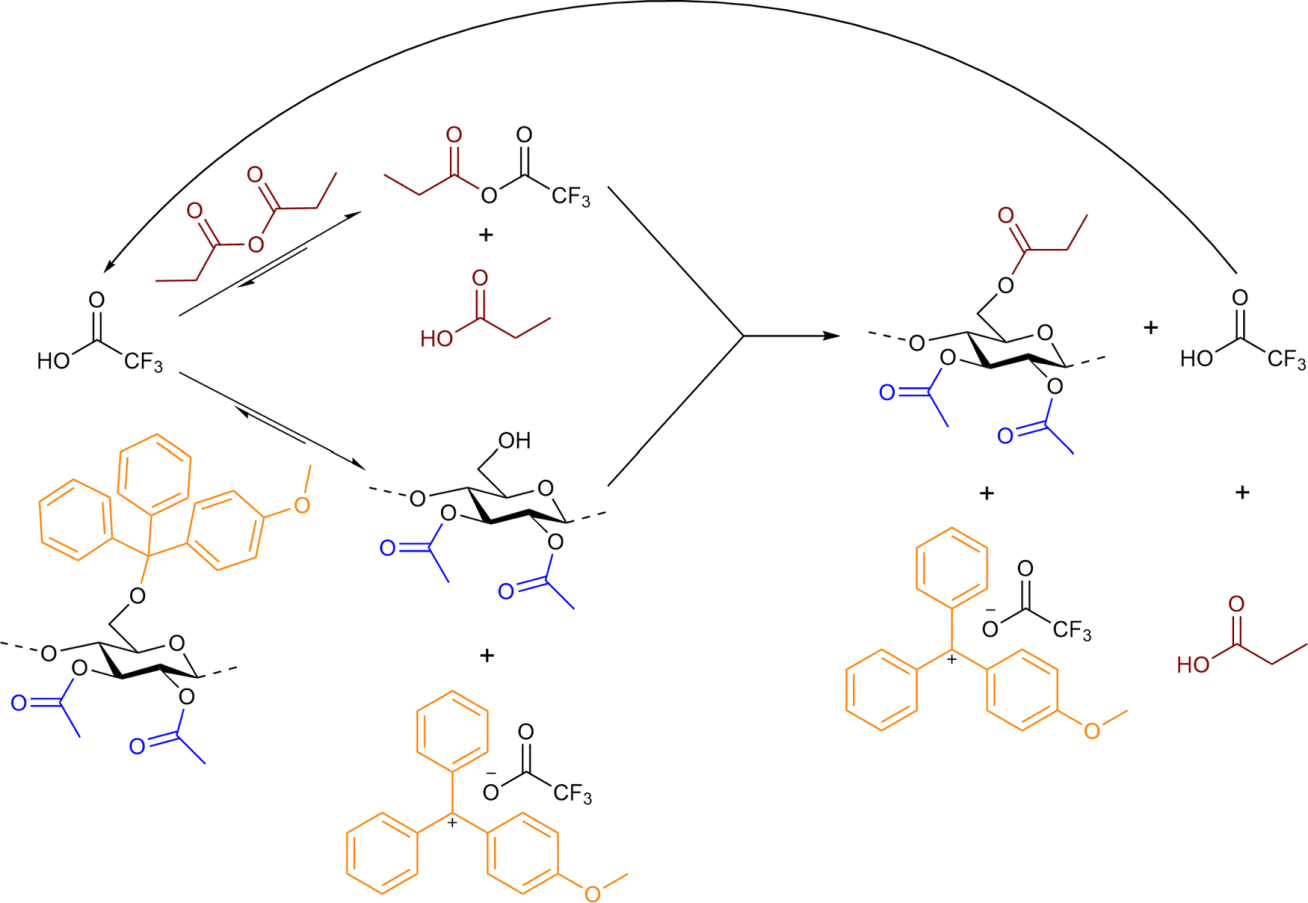
Proposed Transformation of 2,3Ac-6MeOTr Cellulose to 2,3Ac-6Pr Cellulose in Pr_2_O with Excess TFA Acting as Both Detritylating Agent and Acylation Catalyst

After treatment of the 2,3Ac-6MeOTr cellulose ester with TFA dissolved in Pr_2_O, the resulting 2,3-di-*O*-acetyl-6-*O*-propionyl (2,3Ac-6Pr) cellulose could be analyzed via ^1^H NMR in DMSO-*d*_6_ to determine the propionyl (and, therefore, monomethoxytrityl of its precursor) substitution position. Complete detritylation was confirmed by the disappearance of methoxy (3.73 ppm) and aromatic methine (6.77–7.42 ppm) resonances in ^1^H NMR spectra, while propionylation was confirmed by the appearance of new signals at 2.36 and 1.05 ppm, attributed to the propionyl methylene and methyl protons, respectively (Figure 2). Additionally, the resonances associated with C3-*O*-acetyl methyl groups vicinal to methoxytrityl ethers were not present, further providing evidence for detritylation. There was no evidence of incorporation of trifluoroacetyl esters onto the cellulose backbone, as ^1^H NMR DS calculations indicated that all hydroxy groups were substituted with either acetyl or propionyl substituents, and there were no resonances corresponding to the trifluoroacetyl groups in ^13^C NMR spectra (Figure S17).

**Figure 2:**
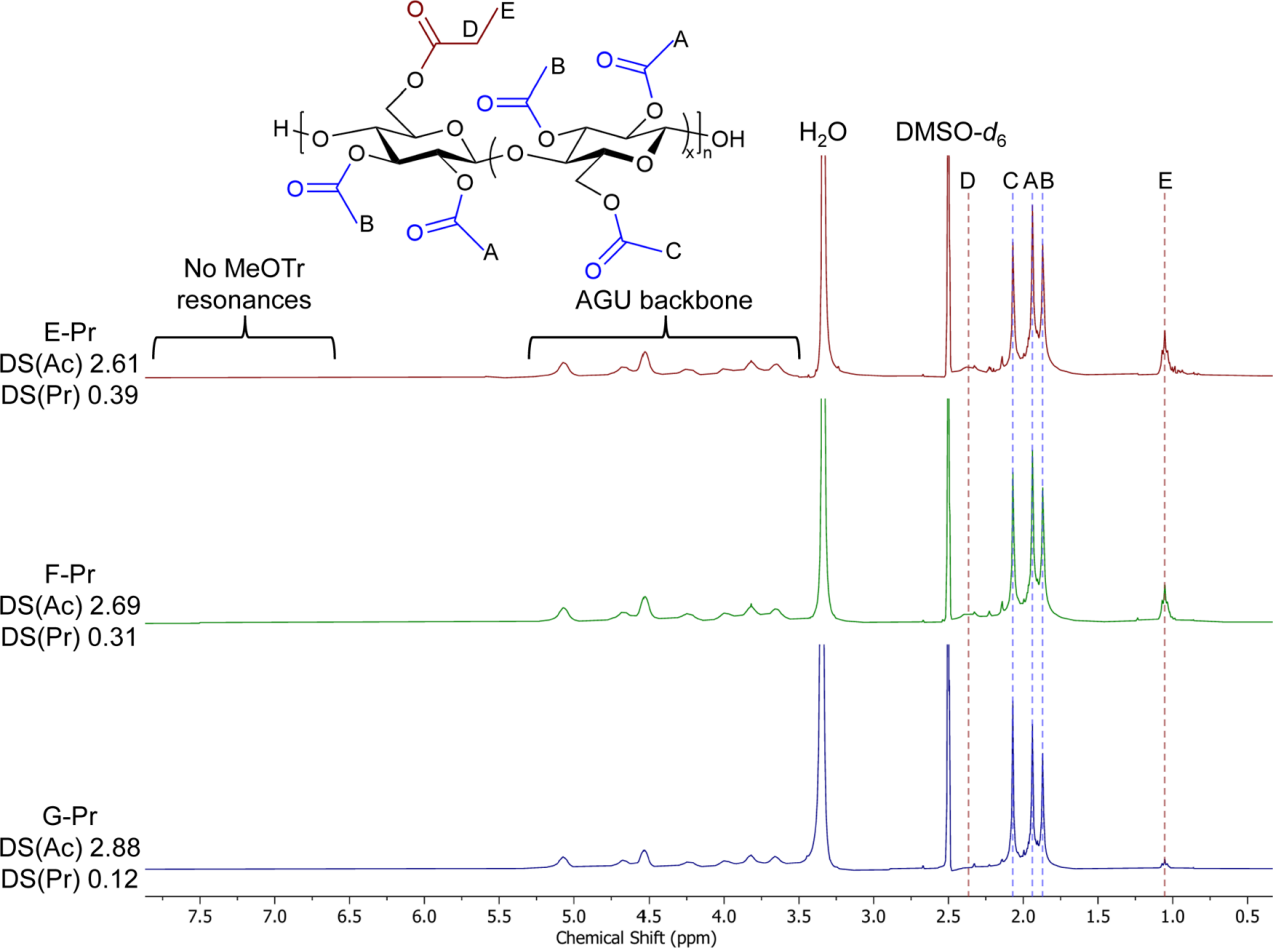
Stacked ^1^H NMR spectra of 2,3Ac-6Pr cellulose DS­(Ac) 2.61–2.88 (E-, F-, and G-Pr).

The terminal methyl protons of propionyl esters appear upfield from the acetyl group methyl protons, permitting clean DS calculation. All samples exhibited quantitative detritylation and propionylation of liberated hydroxy groups according to the DS calculations. Methyl resonances in ^1^H NMR spectra appeared at 1.05 and 0.94 ppm in DMSO-*d*_6_ for the C6 and the C2 substituted propionates, respectively. We achieved relative selectivity for C6 methoxytritylation for intermediate DS­(MeOTr) values (71–88% for DS­(MeOTr) > 0.61, samples A-D-Pr) and high selectivity for C6 methoxytritylation for low DS­(MeOTr) values (90–100% for DS­(MeOTr) < 0.39, samples E-H-Pr) (Table 2). Notably, exclusive C6 methoxytritylation was observed for DS­(MeOTr) 0.01, as there was no observable C2-*O*-propionate methyl resonance at 0.94 ppm in the ^1^H NMR spectrum of sample H–Pr, which also supports prior observations that negligible acyl migration occurs during propionylation (Figure S18). Compared to exclusive cellulose C6 methoxytritylation (76–94%, conducted without *in situ* acylation after methoxytritylation), the one-pot procedure offers comparable selectivity for the C6-OH, indicating that regioselectivity is not impaired in this procedure.^16^ Similar C6 methoxytritylation selectivity was observed for the 2,3-di-*O*-propionyl and 2,3-di-*O*-butyryl derivatives, exhibiting the versatility of this regioselective approach, where Ac_2_O was employed as the acylating agent and reaction medium, generating 2,3-di-*O*-propionyl-6-*O*-acetyl (2,3Pr-6Ac) and 2,3-di-*O*-butyryl-6-*O*-acetyl (2,3Bu-6Ac) cellulose, respectively (Table S3 and Figures S19 and S20). Surprisingly, we observed C3-*O*-acetylation for 2,3Pr-6Ac and 2,3Bu-6Ac cellulose with no detectable C2-*O*-acetylation, which we attribute to potentially incomplete acylation of C3-OH during the initial methoxytritylation/acylation step, consistent with DS calculations indicating that DS­(6-Ac) was equal to DS­(MeOTr).

**Table 2:** Results of Transformation of 2,3Ac-6MeOTr Cellulose to 2,3Ac-6Pr Cellulose with Pr_2_O/TFA

sample	DS(Ac)	DS(6-Pr)	DS(2-Pr)	C6-OH selectivity (%)	*T*_g_ (°C)	*T*_m_ (°C)
A-Pr	1.59	1.00	0.41	71	166	
B-Pr	2.04	0.77	0.19	80	162	
C-Pr	2.34	0.58	0.08	88	165	
D-Pr	2.39	0.51	0.10	84	166	
E-Pr	2.61	0.35	0.04	90	166	
F-Pr	2.69	0.28	0.03	90	167	268
G-Pr	2.88	0.11	0.01	92	169	289
H-Pr	2.99	0.01	0.00	100	171	296

In addition to preventing acyl migration and maintaining C6 regioselectivity, high DP mixed cellulose esters were prepared even with a large molar excess of TFA and generation of propionic acid throughout the reaction (Table S4). Similar to the preparation of 2,3A-6MeOTr cellulose esters, an increase in DP was observed, potentially due to the loss of low-MW fractions during workup. However, a tail in the SEC chromatogram at high elution times (i.e., lower *M*_n_) was observable for both samples E-MeOTr and E-Pr, while the SEC chromatogram of carbanilated MCC (a routine derivative employed to determine *M*_n_ for cellulose) was symmetric (Figure S21). The high elution time shoulder may be indicative of low-MW fractions (although not removed from multiple precipitation steps) produced from chain scission during dissolution in DMAc/LiCl (for the preparation of 2,3Ac-6MeOTr cellulose) or during acid-catalyzed detritylation and reacylation (for the preparation of 2,3Ac-6Pr cellulose). The persistence of this observed low *M*_n_ fraction after detritylation and reacylation makes it difficult to assess the effect of the acid-catalyzed treatment on *Đ* and *M*_n_, although the production of 2,3Ac-6Pr cellulose esters with *M*_n_ > 122.6 kg/mol (DP*_n_* > 419) was possible. It should be emphasized that direct comparison of cellulose ester *M*_n_ and DP_n_ with varying DS and substituent type is difficult, as the varying monosaccharide composition of each cellulose ester may affect solution confirmation and hydrodynamic radii, which will affect the observed MW.

Further evidence of the regioselectivity was obtained by 2D-NMR spectroscopy. HMBC is a particularly useful tool for analyzing positional substitution of cellulose esters through the correlations of AGU ring protons to the carbonyl carbon of substituted acyl groups.^2,23^ The HMBC spectrum of sample F-Pr (DS­(Ac) 2.69) is provided in Figure 3, and distinct correlations at 4.77/169.4 and 5.04/169.9 ppm were observed, corresponding to the H2/C2 acetyl carbonyl and H3/C3 acetyl carbonyl, respectively. No correlations were observed between either H2 or H3 and propionyl carbonyl moieties, further suggesting the high regioselectivity of propionylation (and therefore methoxytritylation) for the C6 position. There were no correlations observed between H6/H6′ and either acetyl or propionyl carbonyl resonances, although our group has previously reported difficulty in observing correlations between H6/H6′ and C6 acyl carbonyl moieties.^2,23^ The HMBC spectra, combined with ^1^H NMR measurements of relative DS and 2D-NMR analyses (Figures S22–S35), provide strong evidence that regioselectivity is maintained during both one-pot tritylation/acylation and detritylation/reacylation procedures.

**Figure 3:**
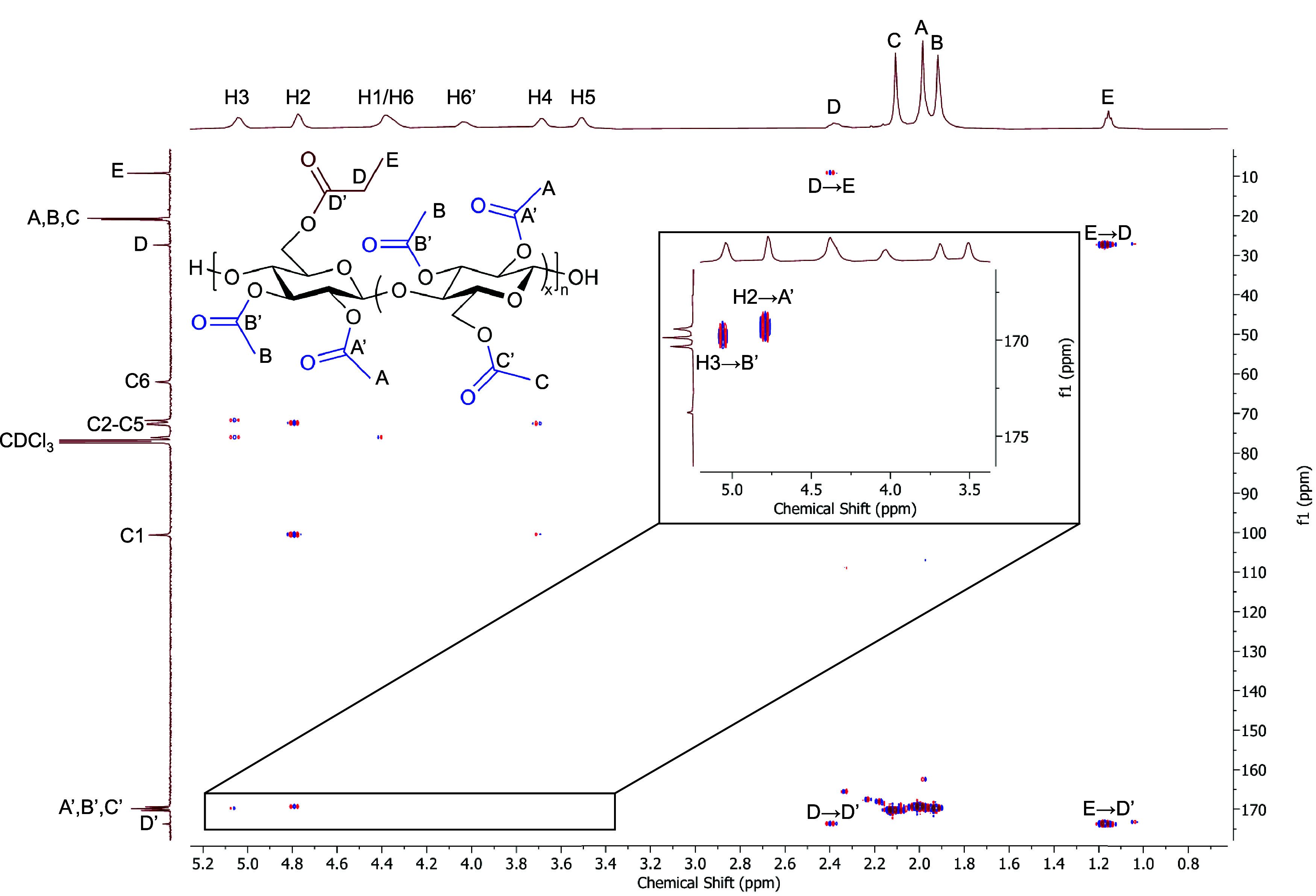
HMBC spectrum of 2,3Ac-6Pr cellulose DS­(Ac) 2.69 (F-Pr).

As expected, we observed a relationship between the glass transition temperature (*T*_g_) and DS­(Pr), where *T*_g_ generally decreased as DS­(Pr) increased. This trend is common in mixed cellulose esters, where *T*_g_ decreases with increasing DS of longer-chain esters and decreasing DS of shorter-chain esters or hydroxy groups.^2,5,7,32^ Increasing ester chain length both increases the available free volume and interferes with intermolecular main-chain interactions, causing a *T*_g_ depression. Sample B-Pr (DS­(Pr) 0.96) exhibited the lowest *T*_g_ of 162 °C while sample H-Pr (DS­(Pr) 0.01) exhibited the highest *T*_g_ of 171 °C, as observed by DSC. Interestingly, sample A-Pr (DS­(Pr) 1.41) exhibited a higher *T*_g_ of 166 °C than sample B-Pr (DS­(Pr) 0.96), which is opposite the trend observed for samples B-H-Pr (Figure S36). As significant tritylation at C2 (and therefore, propionylation at C2) suggests that A-Pr is a random copolymer comprising 2,3-di-*O*-acetyl-6-*O*-propionyl and 3-*O*-acetyl-2,6-di-*O*-propionyl AGU repeating units, we attribute this increase in *T*_g_ to possible specific inter- and intramolecular interactions, similar to the increase in *T*_g_ for some random copolymers of methyl methacrylate and vinylbenzylthymine.^40^ Samples A-E-Pr were completely amorphous and exhibited only a *T*_g_, due to the random copolymeric nature of mixed cellulose esters (Figure S37).^32,41^

We were surprised to see that samples F-H-Pr were semicrystalline with melting endotherms ranging from 268 to 296 °C, respectively (Figure S38), with *T*_m_ increasing linearly with decreasing DS­(Pr) and increasing DS­(Ac) (Figure S39). The high C6-OH tritylation selectivity (≥90%) suggests that samples F-H-Pr are random copolymers comprising 2,3,6-tri-*O*-acetyl and 2,3-di-*O*-acetyl-6-*O*-propionyl repeating units. Since there is no way to control the sequence of repeating units, the distribution of 2,3Ac-6Pr and triacylated monosaccharides is random. As DS­(Pr) increased, the relative fraction of 2,3Ac-6Pr repeating units increased, resulting in shorter uninterrupted stretches of triacylated repeating units capable of crystallizing, causing *T*_m_ to decrease. A similar melting point depression was observed for poly­(ether ketone ketone) (PEKK) treated with NBS in solution, where an increase of randomly distributed noncrystallizable (i.e., brominated) repeating units caused *T*_m_ to decrease compared to unmodified PEKK.^42^

The thermal properties of sample H-Pr approached those of CA-436-80S (Eastman Chemical Company cellulose triacetate DS­(Ac) 2.86, *T*_g_ = 181 °C, *T*_m_ = 295 °C), although sample H-Pr had a noticeably lower *T*_g_ (171 °C) due to the presence of propionate esters and absence of hydroxy groups acting as hydrogen bond donors. This is an exciting result, especially when designing a functionalized CA, as this indicates that the crystallizability of triacetylated AGU repeating units is not impaired by the presence of 2,3Ac-6Pr repeating units. Additionally, 2,3Pr-6Ac and 2,3Bu-6Ac cellulose (samples G-Ac/Pr and G-Ac/Bu, respectively) were semicrystalline and exhibited melting endotherms at 234 and 174 °C, respectively (Table S3 and Figure 4). These melting temperatures are similar to those reported for cellulose tripropionate (*T*_m_ = 235 °C) and cellulose tributyrate (*T*_m_ = 182 °C), further suggesting that stretches of triacylated repeating units maintain crystallizability, with the presence of noncrystallizable repeating units decreasing *T*_m_ relative to that of the triester.^43^

**Figure 4:**
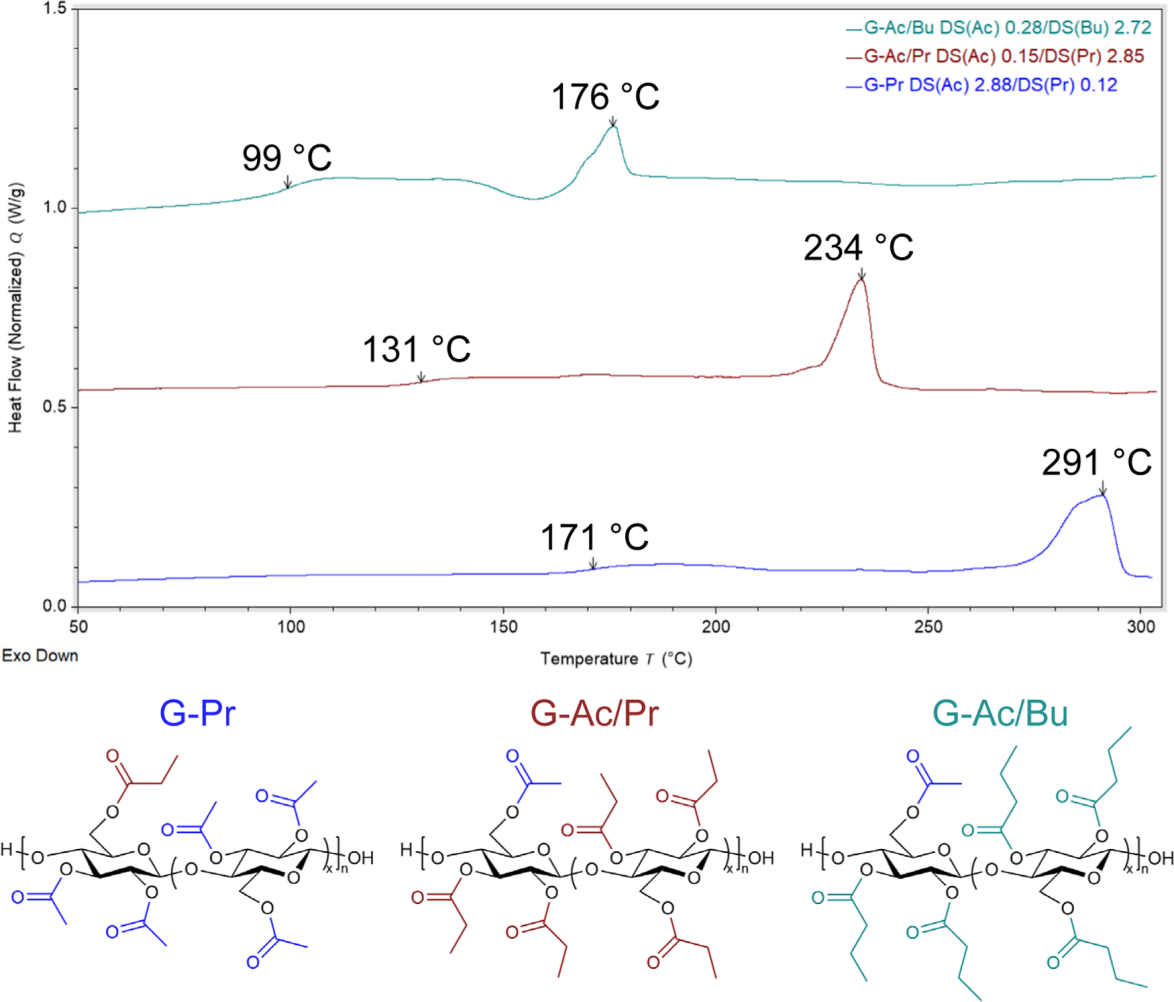
Representative DSC thermograms of 2,3Ac-6Pr cellulose DS­(Ac) 2.88 (G-Pr), 2,3Pr-6Ac cellulose DS­(Pr) 2.85 (G-Ac/Pr), and 2,3Bu-6Ac cellulose DS­(Bu) 2.72 (G-Ac/Bu).

After successful transformation of the 6-MeOTr ether to either the 6-Ac or 6-Pr ester in a one-pot procedure, we wondered whether this approach would be applicable to other carboxylic acid anhydrides. This procedure could be valuable for elucidating structure–property relationships between thermal (i.e., *T*_g_) and viscoelastic properties (i.e., tan δ) of cellulose esters while varying the ester composition exclusively at the C6 position. Since we hypothesize that stretches of triacyl AGU repeating units comprise the crystalline phase and the 2,3Ac-6B repeating units comprise the amorphous phase, we were curious as to whether the *T*_g_ could be modified through selective alteration of the C6 ester moiety, as the glass transition is experienced only by the amorphous phase.

We chose to modify samples F- and G-MeOTr using a procedure similar to that used for one-pot detritylation/propionylation, except for selecting from Bu_2_O, isobutyric (*i*Bu_2_O), and isovaleric (*i*Va_2_O) anhydrides as acylating agents and reaction media. Sample F-MeOTr was readily soluble in Bu_2_O but was not fully soluble in *i*Bu_2_O or *i*Va_2_O until addition of TFA, while sample G-MeOTr only dissolved in the selected acyl anhydrides after adding TFA. Interestingly, G-MeOTr gelled in Bu_2_O/TFA after 1.5 h, gelled in *i*Bu_2_O/TFA after 2.5 h, and gelled in *i*Va_2_O/TFA after 30 min, requiring dilution with DCM to maintain stirring at ambient temperature. In each case, full deprotection of the C6-MeOTr ether was observed according to ^1^H NMR, with no observable deacetylation (Figures 5 and S40). Full conversion to the desired ester was observed for butyryl and isobutyryl derivatives, although isovaleryl derivatives contained some hydroxy groups that were not acylated. This is likely due to some combination of the hydrophobic nature and steric bulk of *i*Va_2_O, which prevents effective wetting of the cellulose ester. DS calculations suggest full acylation of the C6 hydroxy group with only partial acylation of the C2 hydroxy group (Table 3).

**Figure 5:**
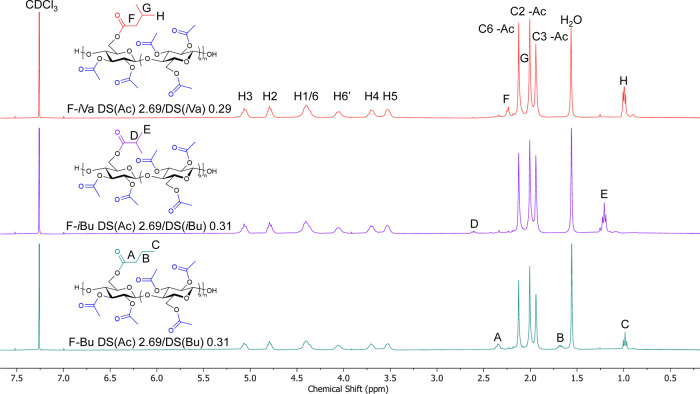
Stacked ^1^H NMR spectra of 2,3Ac-6Bu, 2,3Ac-6*i*Bu, and 2,3Ac-6*i*Va cellulose DS­(Ac) 2.69 (F-Bu, F-*i*Bu, and F-*i*Va).

**Table 3:** Properties of Regioselectively Substituted Mixed 2,3Ac-6B Aliphatic Cellulose Esters Prepared through One-Pot Detritylation/Reacylation^a^

sample	DS(Ac)	DS(B)	DS(OH)	*T*_g_ (°C)	*T*_m_ (°C)
E-Hex	2.61	0.37	0.02	151	
E-Oct	2.61	0.36	0.03	139	
F-Bu	2.69	0.31	0.00	165	263
F-*i*Bu	2.69	0.31	0.00	169	
F-*i*Va	2.69	0.29	0.02	166	
G-Bu	2.88	0.12	0.00	174	288
G-*i*Bu	2.88	0.12	0.00	171	287
G-*i*Va	2.88	0.10	0.02	169	284

a B = butyryl, isobutyryl, isovaleryl, hexanoyl, or octanoyl.

### 3.3 One-Pot Synthesis of 2,3Ac-6B Cellulose Esters from 2,3Ac-6MeOTr Cellulose Employing Acyl Chlorides

Based on encouraging results in synthesizing 2,3A-6B cellulose esters with high regioselectivity and control over DS­(A)/DS­(B), we explored the feasibility of a one-pot/two-step detritylation/acylation reaction employing acyl halides rather than a carboxylic acid anhydride and TFA. One-pot detritylation/acylation with acyl halides was pioneered for the efficient synthesis of *O*-acylated-5-hydroxymethyl-oxazolidinone derivatives,^44^ but has not been reported for the synthesis of regioselectively substituted cellulose derivatives. Bergmeier and Arason used acyl chlorides as the sole reagent, relying on trace impurities of HCl to initiate detritylation while using the generation of acylation coproduct HCl to further propel detritylation.^43^ However, employing a strong acid during the reaction may reduce DP through acidic hydrolysis of the glycosidic linkage, so the careful choice of reaction conditions may be necessary to prevent chain degradation.

We chose to investigate the reactivity of 2,3Ac-6MeOTr cellulose with hexanoyl (HexCl) and octanoyl chlorides (OctCl) as exemplar acyl chlorides. Synthesis of cellulose esters with long-chain alkanoates is challenging and can be very useful, generating self-plasticizing esters.^41^ In our initial forays, treatment of sample E-MeOTr with 5 mol equiv of acyl chloride per AGU in THF at RT for 24 h resulted in only partial detritylation and reacylation, as evidenced by the presence of both aliphatic ester and aromatic MeOTr resonances in ^1^H NMR spectra (Figure S41). Increasing the temperature to 40 °C and the molar ratio of acyl chloride:AGU to 7.5:1, full detritylation as detected by ^1^H NMR was achieved in 20 h without any acetate hydrolysis (Table 3 and Figures S42 and S43). Nearly quantitative reacylation with HexCl and OctCl was observed, with DS­(Hex) 0.37 for sample E-Hex and DS­(Oct) 0.36 for E-Oct. This suggests that all liberated C6 hydroxy groups were acylated with only partial acylation of secondary C2 hydroxy groups, which is reasonable due to the higher reactivity of the C6-OH vs C2-OH. To our knowledge, this is the first documented example of the conversion of a 2,3Ac-6MeOTr cellulose derivative to a 2,3Ac-6B cellulose ester through the sole employment of an acyl chloride.

*T*_g_ depended in a sensible fashion upon both the composition and DS of the C6 moiety (Figure 6). For E-Pr (*T*_g_ = 167 °C), E-Hex (*T*_g_ = 151 °C), and E-Oct (*T*_g_ = 139 °C), *T*_g_ decreased linearly as the acyl chain length of the C6 moiety increased, attributable to the increase in free volume afforded by longer linear acyl tails which inhibit main-chain intermolecular interactions (Figure S44). Danjo and Iwata prepared multiple cellulose triesters to determine the effect of side-chain branching on the physicochemical properties. These derivatives included cellulose tripropionate (*T*_g_ = 125 °C), cellulose tributyrate (*T*_g_ = 87 °C), cellulose tri­(isobutyrate) (*T*_g_ = 115 °C), and cellulose tri­(isovalerate) (*T*_g_ = 79 °C), and followed the trend of *T*_g,Pr_ > *T*_g,*i*Bu_ > *T*_g,Bu_ > *T*_g,*i*Va_.^43^ Our cellulose triesters had much lower DS of longer-chain substituent at C6 (DS ranging from 0.10 to 0.37, Table 3). At these lower DS values, compounded by the variable DS in the series, the longer-chain substituents had a more muted impact on *T*_g_ (165–169 °C, higher because of the rather high DS­(Ac) in this series); no clear trend was observed.

**Figure 6:**
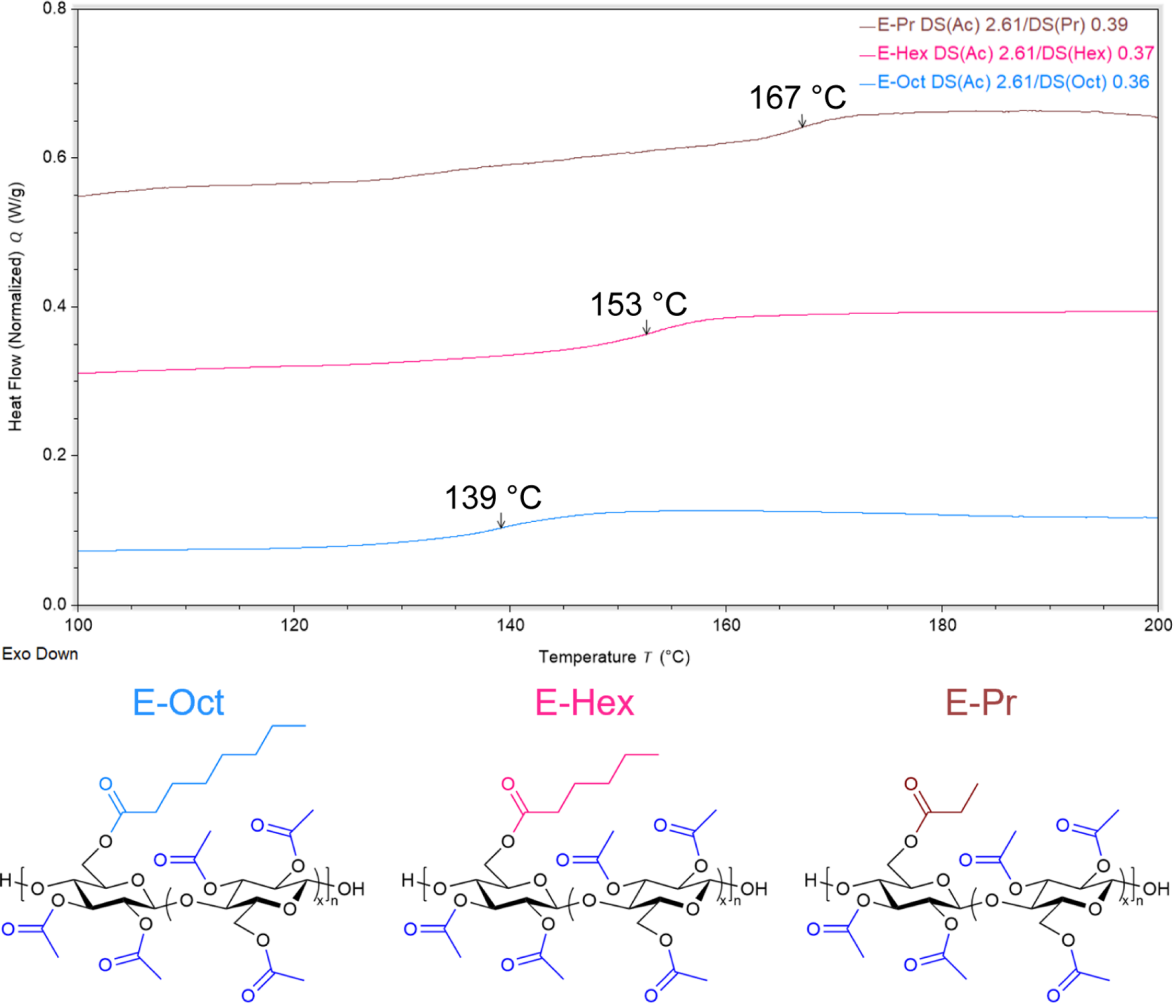
Representative DSC thermograms of 2,3Ac-6Pr, 2,3Ac-6Hex, and 2,3Ac-6Oct cellulose DS­(Ac) 2.61 (E-Pr, E-Hex, and E-Oct).

While F-Pr and F-Bu exhibited melting endotherms at 268 and 263 °C, respectively, neither F-*i*Bu nor F-*i*Va exhibited melting endotherms, suggesting that larger isobutyryl or isovaleryl ester moieties inhibited crystallization of triacetylated AGU repeating units. All aliphatic ester derivatives tested showed improved thermal stability compared with unmodified MCC, with the onset of thermal degradation occurring near 320 °C (Figure S45). All samples derived from G-MeOTr exhibited melting endotherms ranging from 284 °C for G-*i*Va to 296 °C for G-Pr.

### 3.4 One-Pot Synthesis of 2,3Ac-6B Cellulose Ester Macroinitiators from 2,3Ac-6MeOTr Cellulose Employing Acyl Bromides

After demonstrating the reactivity of 2,3Ac-6MeOTr cellulose toward acyl chlorides and identifying effective detritylation/acylation conditions, we were interested in knowing whether acyl bromides could act also as both detritylating and acylating agents. Bromoacyl derivatives, including α-bromoisobutyrate^18,26,45^ and α-bromopropionate^29,46^ esters, are useful macroinitiators in grafting-from polymerizations to prepare polysaccharide graft copolymers. Since acyl bromides are more reactive than acyl chlorides, attributable to the more stable bromide anion formed after hydrolysis, we believed that the desired synthetic transformation could be achieved at ambient temperatures. We chose α-bromoisobutyryl (B*i*BBr) and α-bromopropionyl (BPrBr) bromides as model acyl bromides as both reagents have been used to incorporate initiating sites for controlled radical polymerizations from polysaccharides and could be useful in future studies.

^1^H NMR analysis indicated complete removal of MeOTr moieties from samples F- and G-MeOTr after treatment with B*i*BBr in DCM overnight at RT, indicated by a lack of aromatic methine and methoxy resonances. B*i*B methyl resonances overlapped with the acetyl methyl resonances requiring that we use quantitative ^13^C (q^13^C) NMR to determine DS­(B*i*B). Although full detritylation was observed, reacylation was incomplete, potentially due to water present in the system, which could hydrolyze the acyl bromide faster than the acyl bromide reacted with deprotected C6-OH groups (Table 4). Interestingly, sample E-MeOTr treated with BPrBr in THF exhibited full detritylation and full acylation, as observed in the ^1^H NMR spectrum (Figure S46). Differences between B*i*BBr and BPrBr reactivity indicate that the solvent employed may affect conversion from the protected ether to the desired ester or that the increased steric bulk of the additional methyl group of the B*i*B moiety hinders acylation. HSQC analysis of sample F-B*i*B showed only a single correlation at 1.95/30.6 ppm, corresponding to the B*i*B methyl groups, suggesting that regioselectivity was maintained and little acyl migration took place (Figure S47). While the relatively low DS­(B*i*B) obtained was somewhat disappointing, the ability to synthesize a low DS­(B*i*B) derivative should not be discounted, as subsequent graft copolymer prepared with a low DS of initiating sites (and therefore low graft density) could be useful in preparing blend compatibilizers. This is also the first reported instance of using acyl bromides to convert a cellulosic C6-O-MeOTr ether to a C6-O-α-haloester. It should also be noted that such haloesters are susceptible to nucleophilic substitution with the azide anion,^47^ providing a new route to cellulose esters with regioselective incorporation of “click” chemistry functionality.

**Table 4:** Results from One-Pot Conversion of 2,3Ac-6MeOTr Cellulose to the Desired 2,3Ac-6B Ester Macroinitiator with Acyl Bromides^a^

sample	DS(Ac)	DS(MeOTr)	DS(B)	DS(OH)
E-BPr	2.61	0.00	0.39	0.00
F-B*i*B	2.69	0.00	0.09	0.22
G-B*i*B	2.88	0.00	0.03	0.09
F-MeOTr-B*i*B	2.69	0.26	0.05	0.00
G-MeOTr-B*i*B	2.88	0.07	0.05	0.00

a B = bromopropionyl or bromoisobutyryl.

Having successfully demonstrated the ability of B*i*BBr to act as both a detritylating and acylating agent, we wondered whether adding an acylation catalyst could help increase DS­(B*i*B) during the one-pot detritylation/acylation procedure. We treated samples F- and G-MeOTr with 5 equiv B*i*BBr per AGU and 1.25 equiv 4-dimethylaminopyridine (DMAP) per AGU in anhydrous DCM under dry N_2_ for 16 h (Samples F-MeOTr-B*i*B and G-MeOTr-B*i*B, Table 4). After isolation, we were surprised to observe ^13^C NMR resonances corresponding to both the aromatic resonances of the C6-MeOTr moiety (113.6–159.1 ppm, CDCl_3_) and the bromoisobutyryl methyl groups (30.6 ppm, CDCl_3_) (Figure 7). The addition of DMAP increased DS­(B*i*B) from 0.03 to 0.05 for G-B*i*B-MeOTr, although DS­(B*i*B) decreased from 0.09 to 0.05 for F-B*i*B-MeOTr. The slight change in DS­(B*i*B) in addition to preventing detritylation suggests that DMAP acted primarily as a base to neutralize HBr present during the reaction rather than as an acylation catalyst. This could be a potentially useful route to cellulose derivatives containing two distinct moieties at the C6 position, allowing for further control over cellulose ester microstructure for precise structure–property relationship elucidation. Regardless of the presence or absence of DMAP, high DP cellulose esters containing α-bromoester moieties were obtained after treatment with acyl bromides (Table S5).

**Figure 7:**
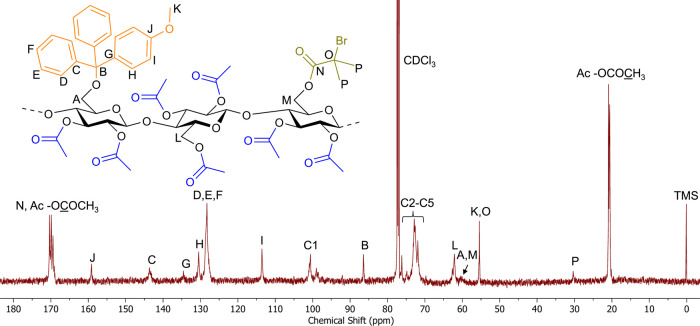
^13^C NMR spectrum of 2,3Ac-6MeOTr-6B*i*B cellulose DS­(Ac) 2.69 (F-MeOTr-B*i*B).

## 4 Conclusions

We have demonstrated an efficient synthetic route to regioselectively substituted 2,3A-6B mixed cellulose esters with control over DS­(A) and DS­(B) by employing two sequential one-pot, two-step transformations. MCC dissolved in DMAc/LiCl could be converted to 2,3A-6MeOTr cellulose with a high tritylation selectivity for C6-OH and minimal backbone degradation. Treatment of 2,3A-6MeOTr cellulose in one of three ways: a carboxylic acid anhydride with TFA, an acyl chloride, or an acyl bromide, all under mild conditions, resulted in detritylation and reacylation of the liberated C6 hydroxy group with no observable acyl migration. Elements of novelty in this work include reduction in isolations from four to two; ability to synthesize cellulose 2,3A-6B esters of many acids with strong regiochemical control and ability to prepare even low 6-DS, avoiding solubility issues; direct replacement of trityl with, for example, α-bromopropionyl esters, creating a useful macroinitiator for graft copolymers (useful in biodegradable plastics and drug delivery, for example); creating a new route to cellulose long-chain esters or esters with branched acids, thereby permitting plastics free of external plasticizers.

These transformations permit investigation of relationships between structure, i.e., DS­(C6) or C6 ester identity, and properties including molecular mobility as measured by DSC; this concept is demonstrated herein by an initial study of thermal properties. We are currently investigating these regioselectively substituted cellulose derivatives to generate high-performance biobased materials, including macroinitiators for controlled radical polymerizations and optical compensation films. They will be subjects of future studies to elucidate structure–property relationships in cellulose ester derivatives.

## Supplementary Material



## Notes

Degree of substitution (DS) calculations; additional synthetic procedures, spectral assignments, and properties of regioselectively substituted 2,3A-6B cellulose esters; FTIR, ^1^H NMR, ^13^C NMR, COSY, HSQC, and HMBC spectra of 2,3A-6MeOTr cellulose esters; ^1^H NMR, ^13^C NMR, COSY, HSQC, and HMBC spectra of 2,3A-6B cellulose esters; COSY spectra of 2,3Ac-6*i*Va cellulose; ^1^H NMR and COSY spectra of 2,3Ac-6Hex and 2,3Ac-6Oct cellulose; representative DSC thermograms of 2,3Ac-6Pr cellulose and Eastman CA-436-80S; plots of *T*_g_ versus DS­(Pr), *T*_m_ versus DS­(Pr), and *T*_g_ versus acyl chain length; representative TGA thermograms of MCC and 2,3Ac-6B cellulose esters; HMBC spectrum of 2,3Ac-6BPr cellulose; and HSQC spectrum of 2,3Ac-6B*i*B cellulose. (PDF)
