# Supplementary material for: Efficient, Regioselective Design of Mixed Cellulose Esters and Macroinitiators
Source: Biomacromolecules. 2025 Aug 21;26(9):5680–93. doi: 10.1021/acs.biomac.5c00432 (PMC12421686; doi:10.1021/acs.biomac.5c00432)
Supplement: Supplementary file 1 [file bm5c00432_si_001.pdf]

Supplementary Information:

Efficient, Regioselective Design of Mixed Cellulose Esters and Macroinitiators

Jeffrey E. Thompson[1*] and Kevin J. Edgar[1,2]

1. Macromolecules Innovation Institute, Virginia Tech, Blacksburg, VA 24061, United States

2. Department of Sustainable Biomaterials, Virginia Tech, Blacksburg, VA 24061, United States

*Corresponding author: Jeffrey E. Thompson – Macromolecules Innovation Institute, Virginia Tech, Blacksburg, VA 24061, United States, Email: jethompson@vt.edu.

Number of pages: 48

Number of equations: 14

Number of figures: 47

Number of tables: 5

Number of procedures: 6

Degree of substitution (DS) calculations

$$1)\quad DS(MeOTr) = \frac{1}{2}\left[\frac{I(MeOTr - CH)}{I(bb) - \frac{3}{14}I(MeOTr - CH)}\right]$$

DS(MeOTr) is the DS of 4-monomethoxytrityl groups in 2,3A-6MeOTr cellulose. I(MeOTr -CH) is the integral of aromatic methine resonances and I(bb) is the integral of cellulose AGU backbone resonances, both obtained from ^{1}H NMR in DMSO-d_6. There are 7 protons per cellulose AGU. There are 3 methoxy protons and 14 aromatic protons per MeOTr moiety. Since the methoxy resonance overlaps with the cellulose backbone, the AGU backbone integral value must be corrected.

$$2)\quad DS(Ac) = \frac{7}{3}\left[\frac{I(Ac - CH_3)}{I(bb) - \frac{3}{14}I(MeOTr - CH)}\right]$$

DS(Ac) is the DS of acetyl groups in 2,3Ac-6MeOTr cellulose. I(Ac -CH_3) is the integral of acetyl methyl resonances, and I(bb) and I(MeOTr -CH) are the same as in Equation 1, with the same backbone correction applied as in Equation 1, both obtained from ^{1}H NMR. There are 7 protons per cellulose AGU and 3 methyl protons per acetyl group.

$$3)\ \ DS(Ac) = \frac{7}{3}\left[\frac{I(Ac - CH_3)}{I(bb)}\right]$$

DS(Ac) is the DS of acetyl groups of detritylated cellulose acetates (CA). I(bb) is the same as in Equation 1 and I(Ac -CH_3) is the same as in Equation 2. There are 7 protons per cellulose AGU and 3 methyl protons per acetyl group.

$$4)\ \ DS(Pr) = \frac{7}{3}\left[\frac{I(Pr - CH_3)}{I(bb)}\right]$$

DS(Pr) is the DS of propionyl groups of detritylated cellulose acetate propionates (CAP). I(bb) is the same as in Equation 1 and I(Pr -CH_3) is the integral of propionyl methyl resonances obtained from ^{1}H NMR. There are 3 methyl protons per propionyl group.

$$5)\ \ C6 - OH\ Selectivity = \frac{DS(6 - Pr)}{DS(2 - Pr) + DS(6 - Pr)} \times 100$$

DS(6-Pr) is the DS of propionyl groups at the C6 position (1.05 ppm) and DS(2-Pr) is the DS of propionyl groups at the C2 position (0.94 ppm) in DMSO-d_6 after transformation of 2,3Ac-6MeOTr cellulose to 2,3Ac-6Pr cellulose in Pr_2O/TFA.

$$6)\ \ DS(Bu) = \frac{7}{3}\left[\frac{I(Bu - CH_3)}{I(bb)}\right]$$

DS(Bu) is the DS of butyryl groups of detritylated cellulose acetate butyrates (CAB). I(bb) is the same as in Equation 1 and I(Bu -CH_3) is the integral of butyryl methyl resonances obtained from ^{1}H NMR. There are 3 methyl protons per butyryl group.

$$7)\ \ DS(iBu) = \frac{7}{6}\left[\frac{I(iBu - CH_3)}{I(bb)}\right]$$

DS(*i*Bu) is the DS of isobutyryl groups of detritylated cellulose acetate isobutyrates (CA*i*B). I(bb) is the same as in Equation 1 and I(*i*Bu -CH_3) is the integral of isobutyryl methyl resonances obtained from ^{1}H NMR. There are 6 methyl protons per isobutyryl group.

$$8)\ \ DS(iVa) = \frac{7}{6}\left[\frac{I(iVa - CH_3)}{I(bb)}\right]$$

DS(*i*Va) is the DS of isovaleryl groups of detritylated cellulose acetate isovalerates (CA*i*V). I(bb) is the same as in Equation 1 and I(*i*Va -CH_3) is the integral of isovaleryl methyl resonances obtained from ^{1}H NMR. There are 6 methyl protons per isovaleryl group.

$$9)\ \ DS(Hex) = \frac{7}{3}\left[\frac{I(Hex - CH_3)}{I(bb)}\right]$$

DS(Hex) is the DS of hexanoyl groups of detritylated cellulose acetate hexanoate (CAH). I(bb) is the same as in Equation 1 and I(Hex -CH_3) is the integral of hexanoyl methyl resonances obtained from ^{1}H NMR. There are 3 methyl protons per hexanoyl group.

$$10)\ \ DS(Oct) = \frac{7}{3}\left[\frac{I(Oct - CH_3)}{I(bb)}\right]$$

DS(Oct) is the DS of octanoyl groups of detritylated cellulose acetate octanoates (CAO). I(bb) is the same as in Equation 1 and I(Oct -CH_3) is the integral of octanoyl methyl resonances obtained from ^{1}H NMR. There are 3 methyl protons per octanoyl group

$$11)\ \ DS(BiB) = \frac{1}{2}\left[\frac{I(BiB - CH_3)}{I(C1)}\right]$$

DS(B*i*B) is DS of bromoisobutyryl groups of detritylated cellulose acetate bromoisobutyrates (CAB*i*B). Unfortunately, the resonances of bromoisobutyryl methyl protons and acetyl methyl protons overlap in ^{1}H NMR, preventing accurate DS calculation. I(B*i*B -CH_3) is the integral of bromoisobutyryl methyl group resonances, and I(C1) is the integral of C1 resonances, both obtained from q^{13}C NMR. There are 2 methyl carbon resonances per B*i*B group and 1 methine carbon C1 resonance per cellulose AGU.

$$12)\ \ DS(BPr) = \frac{7}{3}\left[\frac{I(BPr - CH_3)}{I(bb) - \frac{1}{3}I(BPr - CH_3)}\right]$$

DS(BPr) is the DS of bromopropionyl groups of detritylated cellulose acetate bromopropionates (CABPr). I(BPr -CH_3) is the integral of bromopropionyl methyl groups obtained from ^{1}H NMR. Since the bromopropionyl methine protons overlap with the AGU backbone, the integral value of the backbone must be corrected. There are 3 methyl protons and 1 methine proton per bromopropionyl moiety.

$$13)\quad DS(OH) = 3 - DS(Ac) - DS(X)$$

DS(OH) is the DS of hydroxy groups of detritylated CAB, CA*i*B, CA*i*V, CAH, CAO, CAB*i*B, or CABPr. The maximum DS attainable is 3, DS(Ac) is the DS of acetyl groups, and DS(X) refers to the DS of butyryl, isobutyryl, isovaleryl, hexanoyl, octanoyl, bromoisobutyryl, or bromopropionyl moieites.

$$14)\quad DS(BiB) = 3 - DS(Ac) - DS(Pr)$$

DS(B*i*B) is the DS of bromoisobutyryl groups of partially detritylated CAB*i*B obtained after treatment with Pr_2O/TFA, assuming that all deprotected C6-OMeOTr ethers were converted to C6-OB*i*B esters after treatment with B*i*BBr and DMAP. DS(Ac) is the DS of acetyl groups of DS(Pr) is the DS of propionyl groups after treatment of partially detritylated CAB*i*B after treatment with Pr_2O/TFA.

Synthesis of 2,3-di-*O*-propionyl-6-*O*-(4-monomethoxytrityl) (2,3Pr-6MeOTr) and 2,3-di-*O*-butyryl-6-*O*-(4-monomethoxytrityl) (2,3Bu-6MeOTr) cellulose

MCC was dissolved in DMAc/LiCl and tritylation was conducted as stated in the main text. The synthesis procedure was similar when Pr_2O/Bu_2O were employed as acylating agents, except the solution was stirred for 36 h at 60 °C before cooling to RT and adding to 1.1L 10:1 chilled MeOH:H_2O to precipitate the product. The crude product was isolated by filtration, then redissolved in THF, transferred to 1 kDa MWCO dialysis tubing, and placed in a beaker containing EtOH. The tubing was dialyzed against EtOH for 1 d, acetone for 1 d, then H_2O for 2 d, after point a white precipitate formed inside the tubing. The contents were collected via centrifugation (9000 rpm, 30 min), rinsed with MeOH, then dried under reduced pressure at 50 °C overnight.

<u>2,3Pr-6MeOTr cellulose</u>

Entry G-MeOTr-Pr, Table S5.1. Yield: 1.35 g (63%). ^{1}H NMR (500 MHz, $CDCl_3$): 1.01 (C3 -O-$COCH_2C\underline{H}_3$), 1.04 (C2 -O-$COCH_2C\underline{H}_3$), 1.16 (C6 -O-$COCH_2C\underline{H}_3$), 2.15 (C3 -O-$COC\underline{H}_2CH_3$), 2.22 (C2 -O-$COC\underline{H}_2CH_3$), 2.36 (C6 -O-$COC\underline{H}_2CH_3$), 3.48 (H5), 3.67 (H4), 3.78 (-$C(C_6H_4OC\underline{H}_3)(C_6H_5)_2$), 4.02–4.37 (H6/6'), 4.38 (H1) 4.76 (H2), 5.05 (H3), 6.84–7.44 (-$C(C_6\underline{H}_4OCH_3)(C_6\underline{H}_5)_2$). ^{13}C NMR (125 MHz, $CDCl_3$): 8.9 (C2 -O-$COCH_2\underline{C}H_3$), 9.0 (C3 -O-$COCH_2\underline{C}H_3$), 9.2 (C6 -O-$COCH_2\underline{C}H_3$), 27.2 (C2 -O-$CO\underline{C}H_2CH_3$), 27.3 (C3 -O-$CO\underline{C}H_2CH_3$), 27.4

(C6 -O-CO$\underline{C}H_2CH_3$), 55.4 (-Ph-O$\underline{C}H_3$), 62.0 (C6), 71.7 (C2), 72.3 (C3), 73.0 (C5), 76.0 (C4), 86.4 (-$\underline{C}(C_6H_4OCH_3)(C_6H_5)_2$), 100.5 (C1), 113.5 (Ph -$\underline{C}$H-C-OCH_3), 128.5–143.7 (Ph -$\underline{C}$H), 159.1 (Ph -CH=$\underline{C}$-OCH_3), 172.8 (C2 -O-$\underline{C}OCH_2CH_3$), 173.2 (C3 -O-$\underline{C}OCH_2CH_3$), 173.7 (C6 -O-$\underline{C}OCH_2CH_3$).

2,3Bu-6MeOTr cellulose

Entry G-MeOTr-Bu, Table S5.1. Yield: 1.85 g (77%). ^{1}H NMR (500 MHz, $CDCl_3$): 0.87 (C3 -O-$COCH_2CH_2C\underline{H}_3$), 0.88 (C2 -O-$COCH_2CH_2C\underline{H}_3$), 0.98 (C6 -O-$COCH_2CH_2C\underline{H}_3$), 1.42–1.59 (C2/3 -O-$COCH_2C\underline{H}_2CH_3$), 1.66 (C6 -O-$COCH_2C\underline{H}_2CH_3$), 2.13 (C3 -O-$COC\underline{H}_2CH_2CH_3$), 2.18 (C2 -O-$COC\underline{H}_2CH_2CH_3$), 2.32 (C6 -O-$COC\underline{H}_2CH_2CH_3$), 3.49 (H5), 3.62 (H4), 3.78 (-$C(C_6H_4OC\underline{H}_3)(C_6H_5)_2$), 4.04–4.27 (H6/6'), 4.36 (H1), 4.77 (H2), 5.06 (H3), 6.84–7.44 (-$C(C_6\underline{H}_4OCH_3)(C_6\underline{H}_5)_2$). ^{13}C NMR (125 MHz, $CDCl_3$): 13.7 (C2 -O-$COCH_2CH_2\underline{C}H_3$), 13.8 (C3 -O-$COCH_2CH_2\underline{C}H_3$), 13.9 (C6 -O-$COCH_2CH_2\underline{C}H_3$), 18.2 (C2/3 -O-$COCH_2\underline{C}H_2CH_3$), 18.5 (C6 -O-$COCH_2\underline{C}H_2CH_3$), 35.5 (C2 -O-$CO\underline{C}H_2CH_2CH_3$), 35.8 (C3 -O-$CO\underline{C}H_2CH_2CH_3$), 36.0 (C6 -O-$CO\underline{C}H_2CH_2CH_3$), 55.4 (-Ph-O$\underline{C}H_3$), 62.0 (C6), 71.5 (C2), 72.0 (C3), 73.2 (C5), 75.9 (C4), 86.3 (-$\underline{C}(C_6H_4OCH_3)(C_6H_5)_2$), 100.4 (C1), 113.6 (Ph -$\underline{C}$H-C-OCH_3), 127.6–143.3 (Ph -$\underline{C}$H), 159.1 (Ph -CH=$\underline{C}$-OCH_3), 171.7 (C2 -O-$\underline{C}OCH_2CH_2CH_3$), 172.3 (C3 -O-$\underline{C}OCH_2CH_2CH_3$), 172.8 (C6 -O-$\underline{C}OCH_2CH_2CH_3$).

Synthesis of 2,3Ac-6B cellulose esters using TFA and carboxylic anhydrides

The synthesis procedure was similar for the other carboxylic acid anhydrides used (Bu_2O, *i*Bu_2O, *i*Va_2O), with 20 eq/AGU of TFA and a final concentration of 25 mg/mL. For derivatives of sample G-MeOTr, anhydrous DCM (0.5 mL/50 mg cellulose ester) was added to permit stirring at RT. For workup of 2,3Ac-6Bu cellulose, 2,3Ac-6*i*Bu cellulose, and 2,3Ac-6*i*Va cellulose, the solution was added dropwise to Et_2O and the resulting precipitate was isolated by filtration. The crude product was redissolved in minimal DCM, precipitated in 1:1 EtOH:hexanes, filtered, redissolved in minimal DCM, and precipitated in EtOH. Solid products were then further rinsed with EtOH, collected, air-dried, and then dried under reduced pressure at 50 °C overnight.

2,3-di-*O*-acetyl-6-*O*-butyryl (2,3Ac-6Bu) cellulose

Entry F-Bu, Table 5.3. Yield: 15.6 mg (38%). ^1H NMR (400 MHz, DMSO-d_6): 0.82 (C2 -O-$COCH_2CH_2C\underline{H}_3$), 0.90 (C6 -O-$COCH_2CH_2C\underline{H}_3$), 1.52–1.63 (Bu -O-$COCH_2C\underline{H}_2CH_3$), 1.87 (C3 -O-$COC\underline{H}_3$), 1.94 (C2 -O-$COC\underline{H}_3$), 2.07 (C6 -O-$COC\underline{H}_3$), 2.29–2.38 (Bu -O-$COC\underline{H}_2CH_2CH_3$), 3.67–5.07 (H1–H6/H6'). ^1H NMR (600 MHz, $CDCl_3$): 0.89 (C2 -O-$COCH_2CH_2C\underline{H}_3$), 0.99 (C6 -O-$COCH_2CH_2C\underline{H}_3$), 1.62–1.73 (Bu -O-$COCH_2C\underline{H}_2CH_3$), 1.94 (C3 -O-$COC\underline{H}_3$), 2.01 (C2 -O-$COC\underline{H}_3$), 2.13 (C6 -O-$COC\underline{H}_3$), 2.31–2.38 (Bu -O-$COC\underline{H}_2CH_2CH_3$), 3.53 (H5), 3.71 (H4), 4.06 (H6'), 4.39 (H6), 4.40 (H1), 4.79 (H2), 5.07 (H3).

2,3-di-*O*-acetyl-6-*O*-isobutyryl (2,3Ac-6*i*Bu) cellulose

Entry F-*i*Bu, Table 5.3. Yield: 14.6 mg (35%). ^1H NMR (400 MHz, DMSO-d_6): 0.99 (C2 -O-$COCH(C\underline{H}_3)_2$), 1.12 (C6 -O-$COCH(C\underline{H}_3)_2$), 1.87 (C3 -O-$COC\underline{H}_3$), 1.94 (C2 -O-$COC\underline{H}_3$), 2.07 (C6 -O-$COC\underline{H}_3$), 2.53–2.65 (*i*Bu -O-$COC\underline{H}(CH_3)_2$), 3.66–5.07 (H1–H6/H6'). ^1H NMR (600 MHz, $CDCl_3$): 1.08 (C2 -O-$COCH(C\underline{H}_3)_2$), 1.21 (C6 -O-$COCH(C\underline{H}_3)_2$), 1.94 (C3 -O-$COC\underline{H}_3$), 2.01 (C2 -O-$COC\underline{H}_3$), 2.13 (C6 O-$COC\underline{H}_3$), 2.55–2.67 (*i*Bu -O-$COC\underline{H}(CH_3)_2$), 3.53 (H5), 3.71 (H4), 4.06 (H6'), 4.39 (H6), 4.40 (H1), 4.79 (H2), 5.07 (H3).

2,3-di-*O*-acetyl-6-*O*-isovaleryl (2,3Ac-6*i*Va) cellulose

Entry F-*i*Va, Table 5.3. Yield: 20.8 mg (49%). ^1H NMR (400 MHz, DMSO-d_6): 0.85 (C2 *i*Va -$CH_2CH(C\underline{H}_3)_2$), 0.93 (C6 *i*Va -$CH_2CH(C\underline{H}_3)_2$), 1.87 (C3 -O-$COC\underline{H}_3$), 1.90 (C2 *i*Va -$CH_2C\underline{H}(CH_3)_2$), 1.94 (C2 -O-$COC\underline{H}_3$), 2.05 (C6 *i*Va -$CH_2C\underline{H}(CH_3)_2$), 2.07 (C6 -O-$COC\underline{H}_3$), 2.14–2.23 (*i*Va -$C\underline{H}_2CH(CH_3)_2$), 3.66–5.07 (H1–H6/H6'). ^1H NMR (600 MHz, $CDCl_3$): 0.89 (C2 *i*Va -$CH_2CH(C\underline{H}_3)_2$), 0.99 (C6 *i*Va -$CH_2CH(C\underline{H}_3)_2$), 1.94 (C3 -O-$COC\underline{H}_3$), 1.99 (C2 *i*Va -$CH_2C\underline{H}(CH_3)_2$), 2.01 (C2 -O-$COC\underline{H}_3$), 2.12 (C6 *i*Va -$CH_2C\underline{H}(CH_3)_2$), 2.13 (C6 -O-$COC\underline{H}_3$), 2.22–2.26 (*i*Va -$C\underline{H}_2CH(CH_3)_2$), 3.53 (H5), 3.71 (H4), 4.06 (H6'), 4.39 (H6), 4.40 (H1), 4.79 (H2), 5.07 (H3).

Synthesis of 2,3-di-*O*-propionyl-6-*O*-acetyl (2,3Pr-6Ac) and 2,3-di-*O*-butyryl-6-*O*-acetyl (2,3Bu-6Ac) cellulose esters

For 2,3Pr-6MeOTr and 2,3Bu-6MeOTr cellulose, Ac_2O was employed as the acylating agent and reaction medium with 20 eq/AGU of TFA, a final concentration of 25 mg/mL, and stirring at RT for 16 h under N_2. For 2,3Pr-6Ac cellulose, the solution was added dropwise to 80 mL EtOH to

precipitate the product, isolated via filtration, twice redissolved in THF, and twice reprecipitated in EtOH, after which the resulting white solid was isolated via filtration and dried overnight at 50 ˚C under reduced pressure. For 2,3Bu-6Ac cellulose, the solution was added dropwise to 80 mL 7:1 EtOH:hexanes, isolated via filtration, then washed via centrifugation with 3:1 EtOH:H_2O (3 x 9000 rpm, 30 min, 15 ˚C) before collecting the product and drying overnight at 50 ˚C under reduced pressure.

2,3Pr-6Ac cellulose

Entry G-Ac/Pr, Table S5.2. Yield: 65.3 mg (68%). ^{1}H NMR (600 MHz, $CDCl_3$): 1.02 (C3 -O-$COCH_2C\underline{H}_3$), 1.05 (C2 -O-$COCH_2C\underline{H}_3$), 1.17 (C6 -O-$COCH_2C\underline{H}_3$), 1.90 (C3 -O-$COC\underline{H}_3$), 2.11 (C6 -O-$COC\underline{H}_3$), 2.17 (C3 -O-$COC\underline{H}_2CH_3$), 2.23 (C2 -O-$COC\underline{H}_2CH_3$), 2.38 (C6 -O-$COC\underline{H}_2CH_3$), 3.49 (H5), 3.68 (H4), 4.03 (H6’), 4.36 (H6), 4.38 (H1), 4.78 (H2), 5.03 ($\underline{H}$3 -O-$COCH_3$), 5.06 ($\underline{H}$3 -O-$COCH_2CH_3$). ^{13}C NMR (150 MHz, $CDCl_3$): 9.0 (C3 -O-$COCH_2\underline{C}H_3$), 9.1 (C2 -O-$COCH_2\underline{C}H_3$), 9.2 (C6 -O-$COCH_2\underline{C}H_3$), 20.6 (C3 -O-$CO\underline{C}H_3$), 20.9 (C6 -O-$CO\underline{C}H_3$), 27.2 (C3 -O-$CO\underline{C}H_2CH_3$), 27.4 (C2 -O-$CO\underline{C}H_2CH_3$), 27.5 (C6 -O-$CO\underline{C}H_2CH_3$), 62.1 (C6), 71.8 (C2), 72.3 ($\underline{C}$3 -O-$COCH_2CH_3$), 72.6 ($\underline{C}$3 -O-$COCH_3$) 73.1 (C5), 76.0 (C4), 100.5 (C1), 169.8 (C3 -O-$\underline{C}OCH_3$), 170.3 (C6 -O-$\underline{C}OCH_3$), 172.8 (C2 -O-$\underline{C}OCH_2CH_3$), 173.2 (C3 -O-$\underline{C}OCH_2CH_3$), 173.7 (C6 -O-$\underline{C}OCH_2CH_3$).

2,3Bu-6Ac cellulose

Entry G-Ac/Bu, Table S5.2. Yield: 39.3 mg (42%). ^{1}H NMR (600 MHz, $CDCl_3$) 0.88 (C3 -O-$COCH_2CH_2C\underline{H}_3$), 0.89 (C2 -O-$COCH_2CH_2C\underline{H}_3$), 0.98 (C6 -O-$COCH_2CH_2C\underline{H}_3$), 1.51–1.57 (C2/C3 -O-$COCH_2C\underline{H}_2CH_3$), 1.67 (C6 -O-$COCH_2C\underline{H}_2CH_3$), 1.90 (C3 -O-$COC\underline{H}_3$), 2.11 (C6 -O-$COC\underline{H}_3$), 2.13 (C3 -O-$COC\underline{H}_2CH_2CH_3$), 2.19 (C2 -O-$COC\underline{H}_2CH_2CH_3$), 2.33 (C6 -O-$COC\underline{H}_2CH_2CH_3$), 3.50 (H5), 3.63 (H4), 4.04 (H6’), 4.37 (H6), 4.38 (H1) 4.78 (H2), 5.02 ($\underline{H}$3 -O-$COCH_3$), 5.07 ($\underline{H}$3 -O-$COCH_2CH_2CH_3$). ^{13}C NMR (150 MHz, $CDCl_3$): 13.7 (C3 -O-$COCH_2CH_2\underline{C}H_3$), 13.8 (C2 -O-$COCH_2CH_2\underline{C}H_3$), 13.9 (C6 -O-$COCH_2CH_2\underline{C}H_3$), 18.2 (C2/C3 -O-$COCH_2\underline{C}H_2CH_3$), 18.5 (C6 -O-$COCH_2\underline{C}H_2CH_3$), 20.6 (C3 -O-$CO\underline{C}H_3$), 20.9 (C6 -O-$CO\underline{C}H_3$), 35.8 (C2/C3 -O-$CO\underline{C}H_2CH_2CH_3$), 36.0 (C6 -O-$CO\underline{C}H_2CH_2CH_3$), 62.0 (C6), 71.6 (C2), 71.9 ($\underline{C}$3 -O-$COCH_2CH_2CH_3$), 72.6 ($\underline{C}$3 -O-$COCH_3$), 73.2 (C5), 76.0 (C4), 100.3 (C1), 169.8 (C3 -O-$\underline{C}OCH_3$), 170.3 (C6 -O-$\underline{C}OCH_3$), 171.8 (C2 -O-$\underline{C}OCH_2CH_2CH_3$), 172.4 (C3 -O-$\underline{C}OCH_2CH_2CH_3$), 172.9 (C6 -O-$\underline{C}OCH_2CH_2CH_3$).

Synthesis of 2,3Ac-6B cellulose esters using acyl halides

The synthesis procedure was similar to that of acyl bromides in THF (25 mg/mL), except 7.5 eq/AGU of acyl chloride (HexCl or OctCl) was used and the reaction solution was stirred under dry N_2 at 40 °C for 20 h. The solution was then added dropwise to 40 mL 1:1 EtOH:hexanes to precipitate the product, which was isolated by filtration, twice redissolved in minimal THF, and twice reprecipitated in 1:1 EtOH:hexanes. The resulting product was isolated by filtration, rinsed with EtOH and IPA, collected, and dried under reduced pressure at 50 °C overnight.

2,3-di-*O*-acetyl-6-*O*-hexanoyl (2,3Ac-6Hex) cellulose

50 mg 2,3Ac-6MeOTr cellulose (E-MeOTr, DS(Ac) 2.61, DS(MeOTr) 0.39, 0.13 mmol AGU), 2 mL THF, 0.13 mL HexCl (0.95 mmol, 7.5 eq/AGU). Entry E-Hex, Table 5.3. Yield: 25.7 mg (74%). ^{1}H NMR (600 MHz, $CDCl_3$): 0.91 (Hex -O-$CO(CH_2)_4C\underline{H}_3$), 1.31–1.33 (Hex -O-$COCH_2CH_2(C\underline{H}_2)_2CH_3$), 1.63 (Hex -O-$COCH_2C\underline{H}_2(CH_2)_2CH_3$), 1.94 (C3 -O-$COC\underline{H}_3$), 2.01 (C2 -O-$COC\underline{H}_3$), 2.13 (C6 -O-$COC\underline{H}_3$), 2.35 (Hex -O-$COC\underline{H}_2(CH_2)_3CH_3$), 3.52 (H5), 3.71 (H4), 4.05 (H6’), 4.38 (H6), 4.39 (H1), 4.79 (H2), 5.05 (H3).

2,3-di-*O*-acetyl-6-*O*-octanoyl (2,3Ac-6Oct) cellulose

50 mg 2,3Ac-6MeOTr cellulose (E-MeOTr, DS(Ac) 2.61, DS(MeOTr) 0.39, 0.13 mmol AGU), 2 mL THF, 0.16 mL OctCl (0.95 mmol, 7.5 eq/AGU). Entry E-Hex, Table 5.3. Yield: 22.3 mg (55%). ^{1}H NMR (600 MHz, $CDCl_3$): 0.88 (Oct -O-$CO(CH_2)_6C\underline{H}_3$), 1.28–1.31 (Oct -O-$COCH_2CH_2(C\underline{H}_2)_4CH_3$), 1.62 (Oct -O-$COCH_2C\underline{H}_2(CH_2)_4CH_3$), 1.94 (C3 -O-$COC\underline{H}_3$), 2.01 (C2 -O-$COC\underline{H}_3$), 2.13 (C6 -O-$COC\underline{H}_3$), 2.35 (Oct -O-$COC\underline{H}_2(CH_2)_5CH_3$), 3.52 (H5), 3.71 (H4), 4.05 (H6’), 4.38 (H6), 4.39 (H1), 4.79 (H2), 5.05 (H3).

The synthesis procedure for 2,3Ac-6BPr cellulose was similar to that provided for the synthesis of 2,3Ac-6B*i*B cellulose in Section 2.5, except 5 eq BPrBr per AGU was employed.

2,3-di-*O*-acetyl-6-*O*-bromopropionyl (2,3Ac-6BPr) cellulose

250 mg 2,3Ac-6MeOTr cellulose (E-MeOTr, DS(Ac) 2.61, DS(MeOTr) 0.39, 0.66 mmol AGU), 10 mL THF, 0.35 mL BPBr (3.31 mmol, 5 eq/AGU). Entry E-BPr, Table 5.4. Yield: 199 mg (93%). ^{1}H NMR (600 MHz, $CDCl_3$): 1.77 (C2 -O-$COCHBrC\underline{H}_3$), 1.84 (C6 -O-$COCHBrC\underline{H}_3$), 1.92 (C3 -O-$COC\underline{H}_3$), 1.99 (C2 -O-$COC\underline{H}_3$), 2.11 (C6 -O-$COC\underline{H}_3$), 3.52 (H5), 3.69 (H4), 4.04–4.35

(C6 -$C\underline{H}_2$-O-$COCH_3$), 4.12–4.49 (C6 -$C\underline{H}_2$-O-$COCHBrCH_3$), 4.40 (H1), 4.43–4.47 (C2/C6 -O-$COC\underline{H}BrCH_3$), 4.78 (H2), 5.05 (H3). ^{13}C NMR (150 MHz, $CDCl_3$): 20.6 (C3 -O-$CO\underline{C}H_3$), 20.7 (C2 -O-$CO\underline{C}H_3$), 20.9 (C6 -O-$CO\underline{C}H_3$), 21.3–21.7 (C2/C6 -O-$COCHBr\underline{C}H_3$), 39.4 (C2 -O-$CO\underline{C}HBrCH_3$), 39.8 (C6 -O-$CO\underline{C}HBrCH_3$), 62.1 (C6 -$\underline{C}H_2$-O-$COCH_3$), 63.2 (C6 -$\underline{C}H_2$-O-$COCHBrCH_3$), 71.8 (C2), 72.6 (C3), 72.8 (C5), 76.1 (C4), 100.6 (C1), 169.4 (C2 -O-$\underline{C}OCH_3$), 169.6 (C6 -O-$\underline{C}OCHBrCH_3$), 169.7 (C2 -O-$\underline{C}OCHBrCH_3$), 169.9 (C3 -O-$\underline{C}OCH_3$), 170.4 (C6 -O-$\underline{C}OCH_3$).

Carbanilation of MCC

Cellulose was converted to its tricarbanilate according to an existing literature procedure for SEC analysis.[1] To an oven dry 50 mL round bottom 2-neck flask flushed with dry N_2 and equipped with a N_2 inlet, rubber septum, and magnetic stir bar, MCC (100 mg, 0.62 mmol AGU) was suspended in dry pyridine (20 mL, 248.3 mmol, 400 eq/AGU) under dry N_2. PhNCO (2 mL, 18.3 mmol, 30 eq/AGU) was added dropwise to the slurry under dry N_2. The flask was placed in an oil bath equilibrated at 80 ˚C and allowed to stir at that temperature for 24 h. The flask was then removed from heat and allowed to cool to RT, after which 2 mL MeOH was added to the flask to quench remaining unreacted PhNCO. The solution was then added dropwise to 200 mL 3:7 MeOH:H_2O to precipitate the product. The product was then isolated by filtration. The product was then collected and resuspended in EtOH, after which it was isolated again by filtration and rinsed extensively with EtOH, then MeOH, then H_2O. The resulting white powder was collected, air dried, and then dried under reduced pressure at 50 ˚C overnight. Yield: 110 mg (35%). ^1H NMR (400 MHz, DMSO-d_6) 3.52–5.19 (H1–H6/H6’), 6.63–7.42 (C2/C3/C6 -O-CONH-$C_6\underline{H}_5$), 9.09–9.41 (C2/C3/C6 -O-CON$\underline{H}$-C_6H_5).

Table S1: Results of one-pot synthesis of 2,3Pr-6MeOTr and 2,3Bu-6MeOTr celluloses in DMAc/LiCl with same tritylation conditions as sample G-MeOTr (A = propionyl or butyryl).

**Sample**	**4-MeOTrCl:AGU (Equiv)**	**Tritylation time at 70 ˚C (h)**	**DS(MeOTr)**	**DS(A)**
G-MeOTr-Pr	0.25:1	1	0.08	2.85
G-MeOTr-Bu	0.25:1	1	0.13	2.72

Table S2: SEC results of selected 2,3A-6MeOTr cellulose esters (A = acetyl or butyryl).

**Sample**	M_n **(kg/mol)**	DP_n	*Đ*	**dn/dc**
A-MeOTr	141.3	231	2.67	0.086
E-MeOTr	69.7	184	1.82	0.065
F-MeOTr	70.9	197	1.86	0.060
G-MeOTr	176.7	559	4.43	0.034
G-MeOTr-Bu	91.7	236	3.06	0.038
H-MeOTr	176.9	609	3.01	0.027

Table S3: Results of transformation of 2,3Pr-6MeOTr or 2,3Bu-6MeOTr cellulose to 2,3Pr-6Ac or 2,3Bu-6Ac cellulose after treatment with Ac_2O/TFA (A = propionyl or butyryl).

Sample	DS(A)	DS(6-Ac)	DS(3-Ac)	DS(2-Ac)	Selectivity	T_g (˚C)	T_m (˚C)
G-Ac/Pr	2.85	0.08	0.07	0.00	100%	130	234
G-Ac/Bu	2.72	0.13	0.15	0.00	100%	99	174

Table S4: SEC results of selected 2,3A-6B cellulose esters prepared using TFA and carboxylic acid anhydrides (A = acetyl or propionyl, B = propionyl, isovaleryl, isobutyryl, or acetyl).

Sample	M_n (kg/mol)	DP_n	*Đ*	dn/dc
A-Pr	146.3	475	2.03	0.038
E-Pr	122.6	419	1.89	0.034
F-*i*Va	103.1	301	1.68	0.030
G-*i*Bu	367.3	1260	4.95	0.020
H-Pr	185.1	642	3.59	0.027

Table S5: SEC results of selected 2,3Ac-6B cellulose esters prepared employing acyl bromides (B = bromopropionyl or bromoisobutyryl).

Sample	M_n (kg/mol)	DP_n	*Đ*	dn/dc
E-BPr	147.5	455	2.23	0.035
F-B*i*B	81.1	281	1.73	0.039
F-MeOTr-B*i*B	94.6	268	1.84	0.058

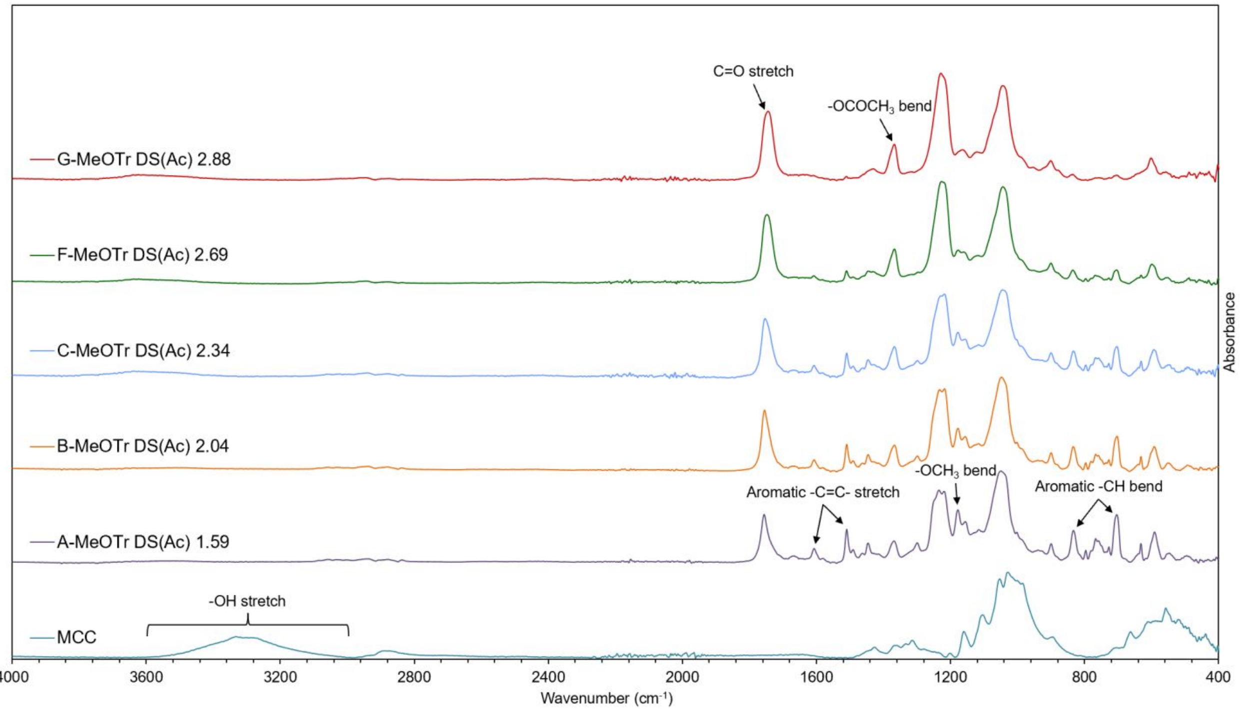

<u>**Figure S1:** Stacked FTIR spectra of MCC and 2,3Ac-6MeOTr celluloses with varying DS(Ac)/DS(MeOTr).</u>

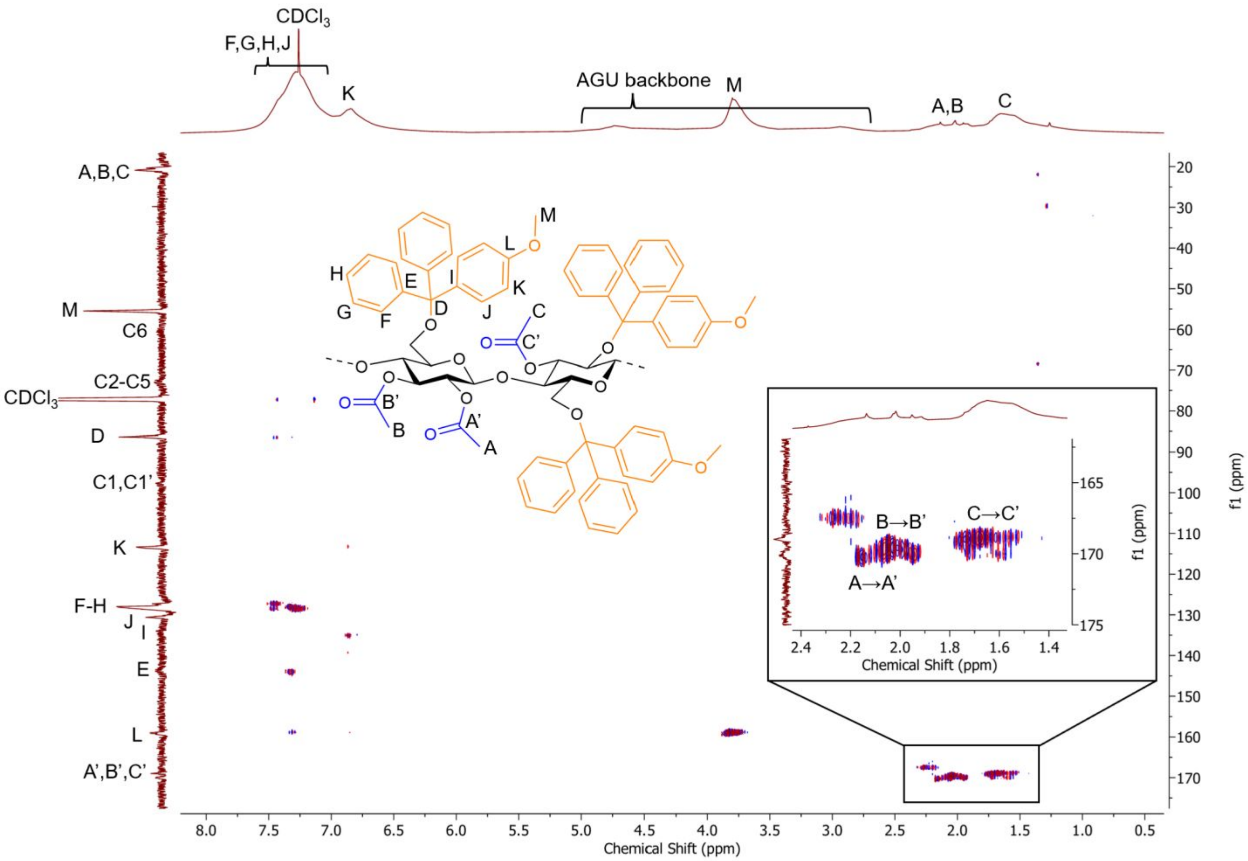

Figure S2: HMBC spectrum of 2,3Ac-6MeOTr cellulose DS(Ac) 1.59 (A-MeOTr).

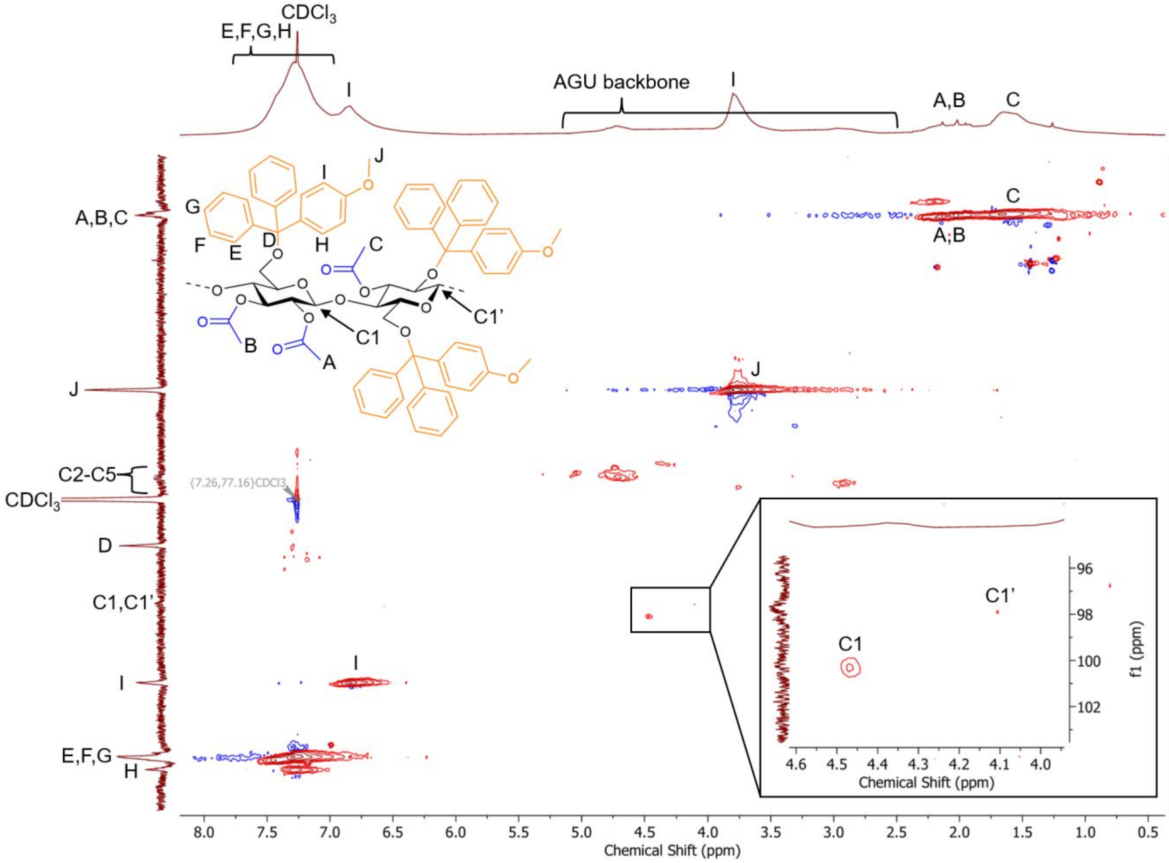

<u>**Figure S3:** HSQC spectrum of 2,3Ac-6MeOTr cellulose DS(Ac) 1.59 (A-MeOTr).</u>

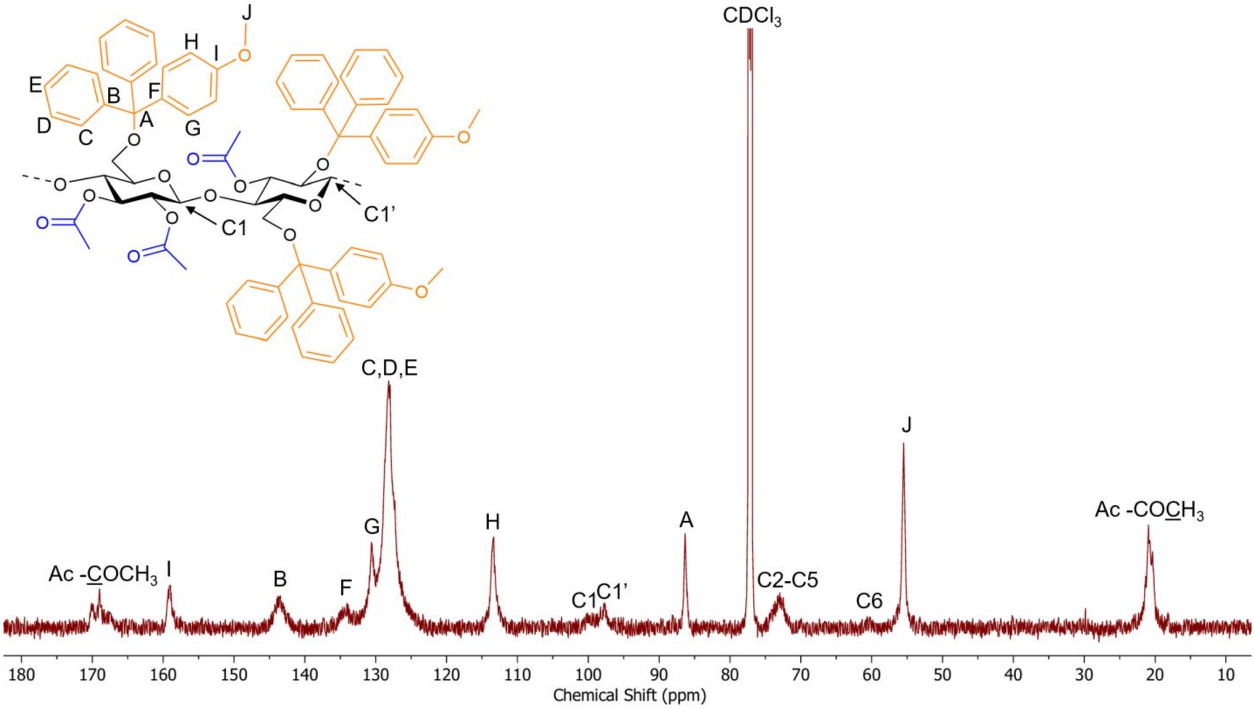

<u>**Figure S4:** ^{13}C NMR spectrum of 2,3Ac-6MeOTr cellulose DS(Ac) 1.59 (A-MeOTr).</u>

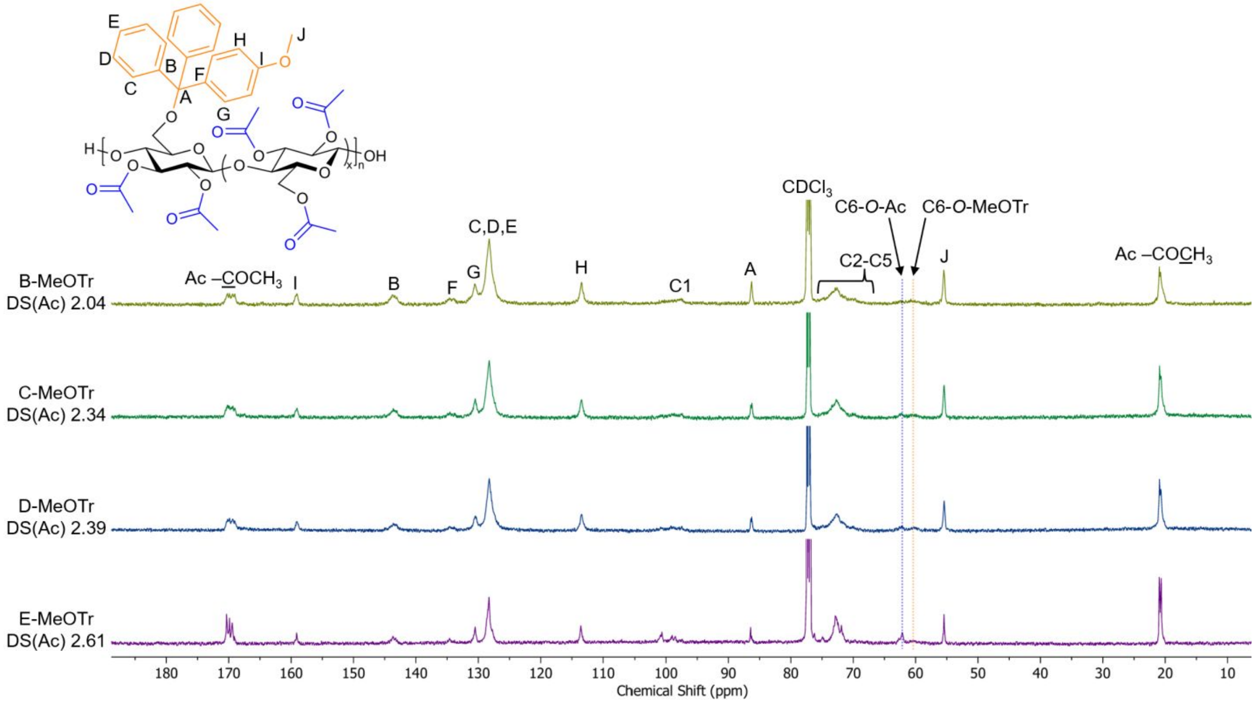

Figure S5: Stacked ^{13}C NMR spectra of 2,3Ac-6MeOTr cellulose DS(Ac) 2.04–2.61 (B–E-MeOTr).

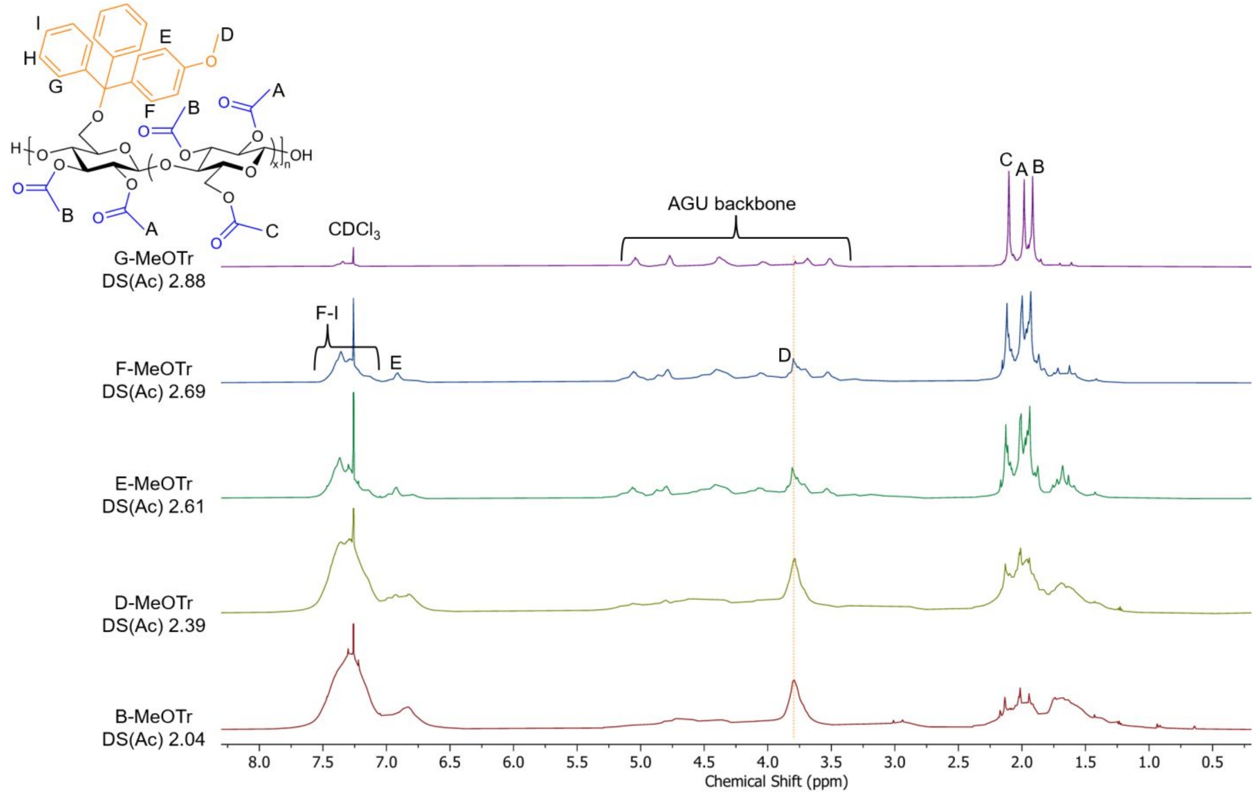

Figure S6: Stacked ^{1}H NMR spectra of 2,3Ac-6MeOTr cellulose DS(Ac) 2.04–2.88 (B-, D-, E-, F-, and G-MeOTr).

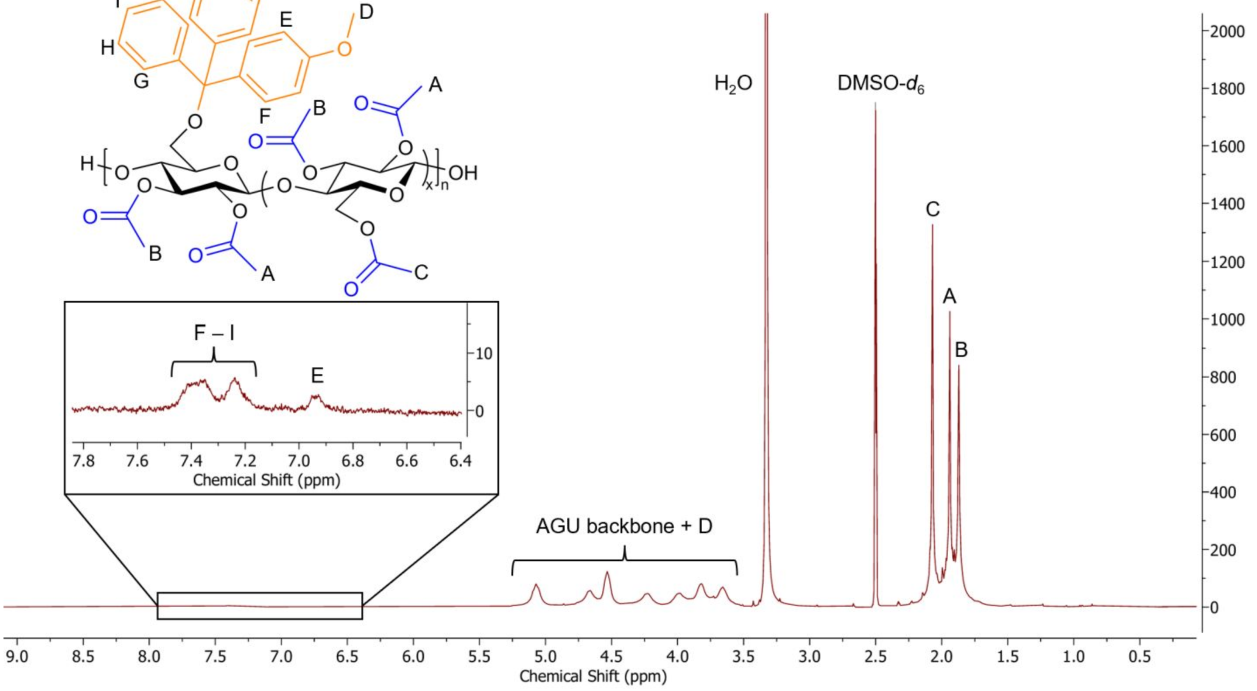

Figure S7: ^{1}H NMR spectrum of 2,3Ac-6MeOTr cellulose DS(Ac) 2.99 (H-MeOTr).

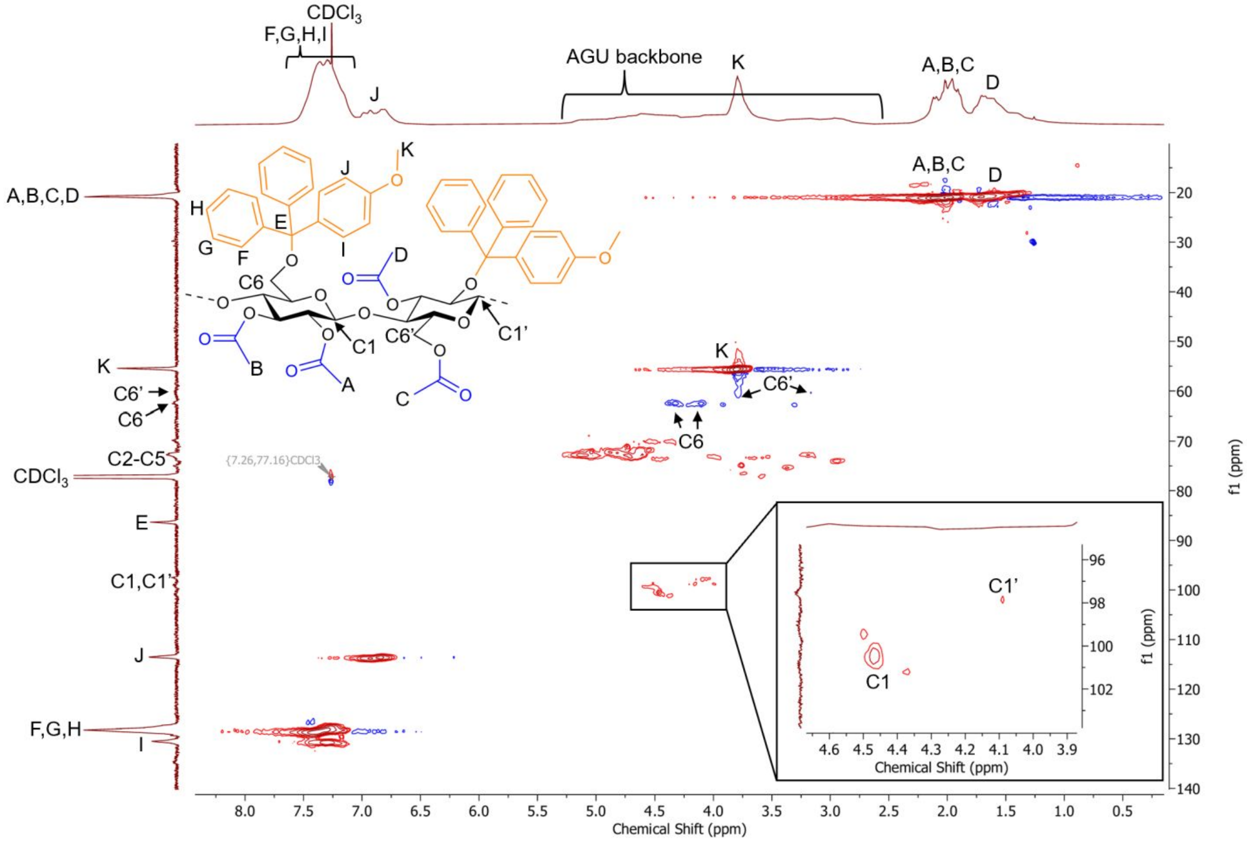

Figure S8: HSQC spectrum of 2,3Ac-6MeOTr cellulose DS(Ac) 2.34 (C-MeOTr).

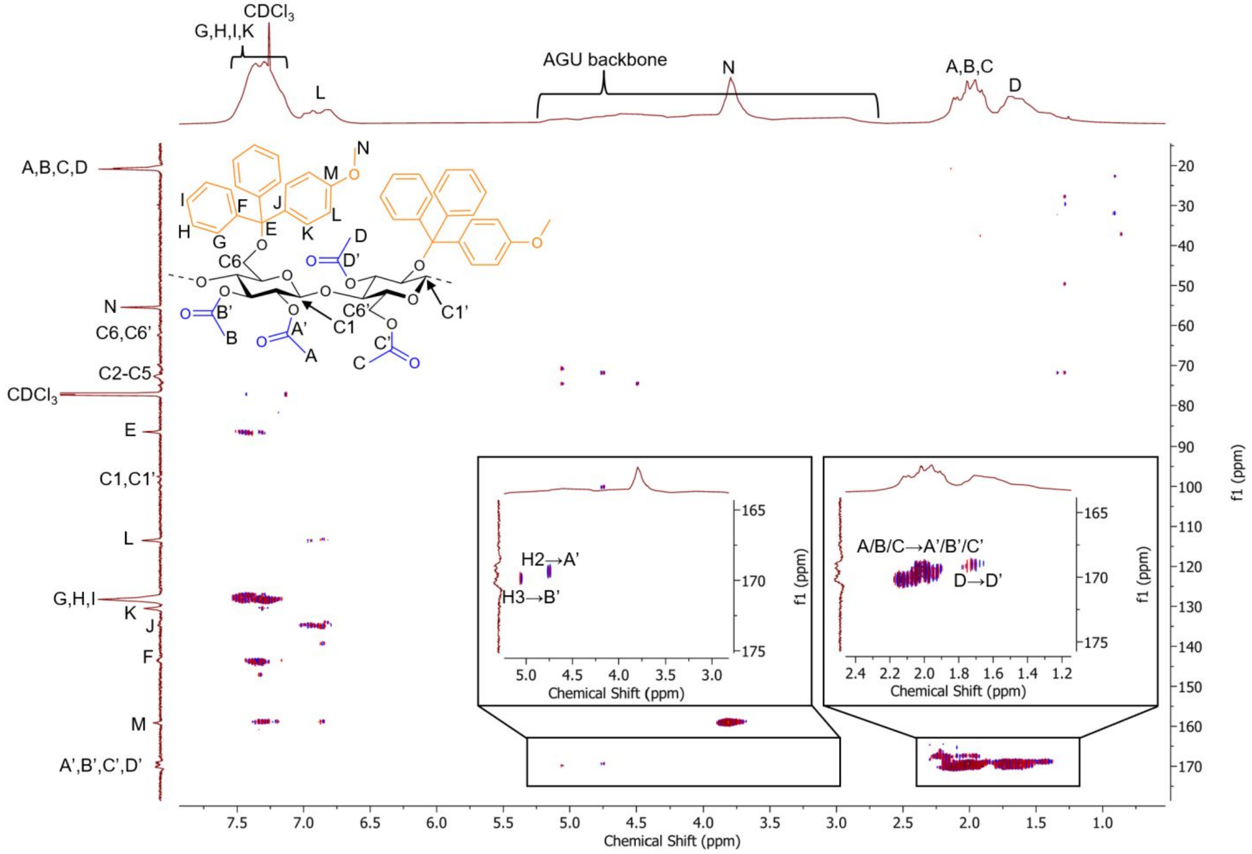

Figure S9: HMBC spectrum of 2,3Ac-6MeOTr cellulose DS(Ac) 2.34 (C-MeOTr).

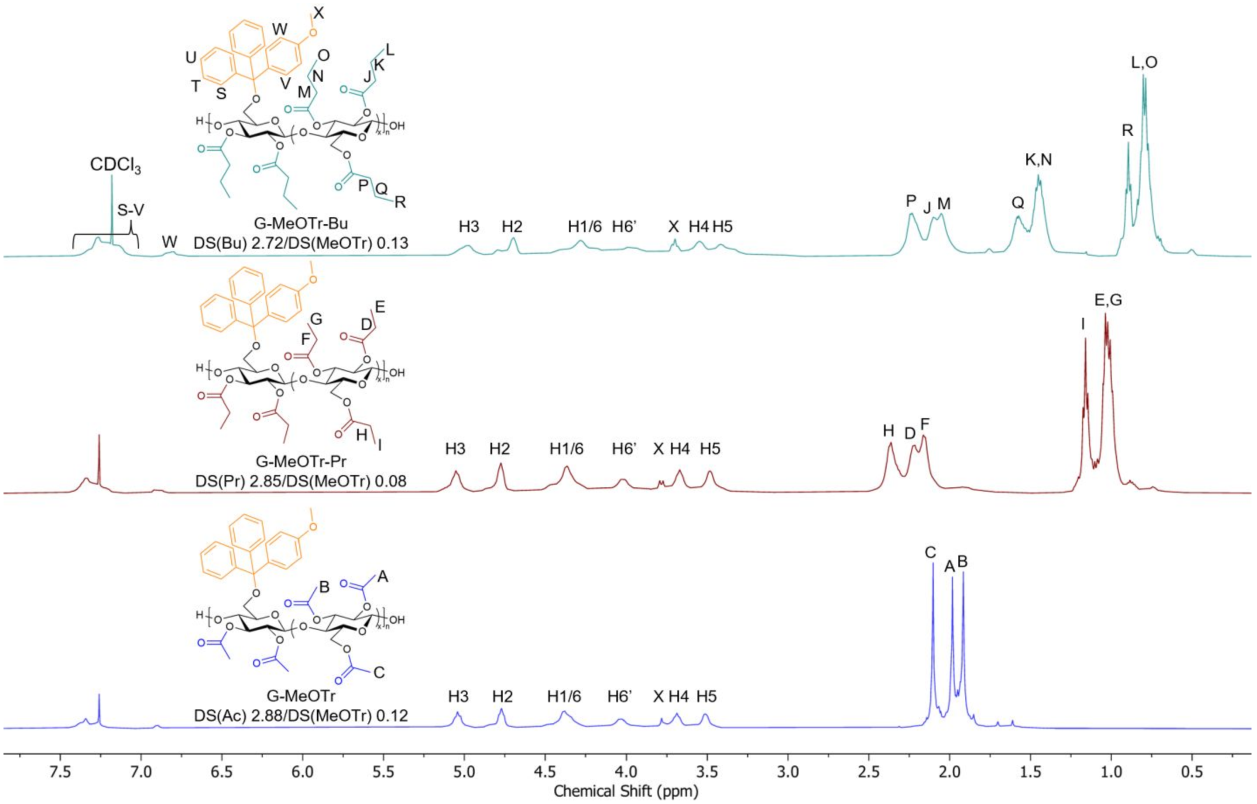

Figure S10: Stacked ^{1}H NMR spectra of 2,3Ac-6MeOTr cellulose DS(Ac) 2.88 (G-MeOTr), 2,3Pr-6MeOTr cellulose DS(Pr) 2.85 (G-MeOTr-Pr), and 2,3Bu-6MeOTr cellulose DS(Bu) 2.72 (G-MeOTr-Bu).

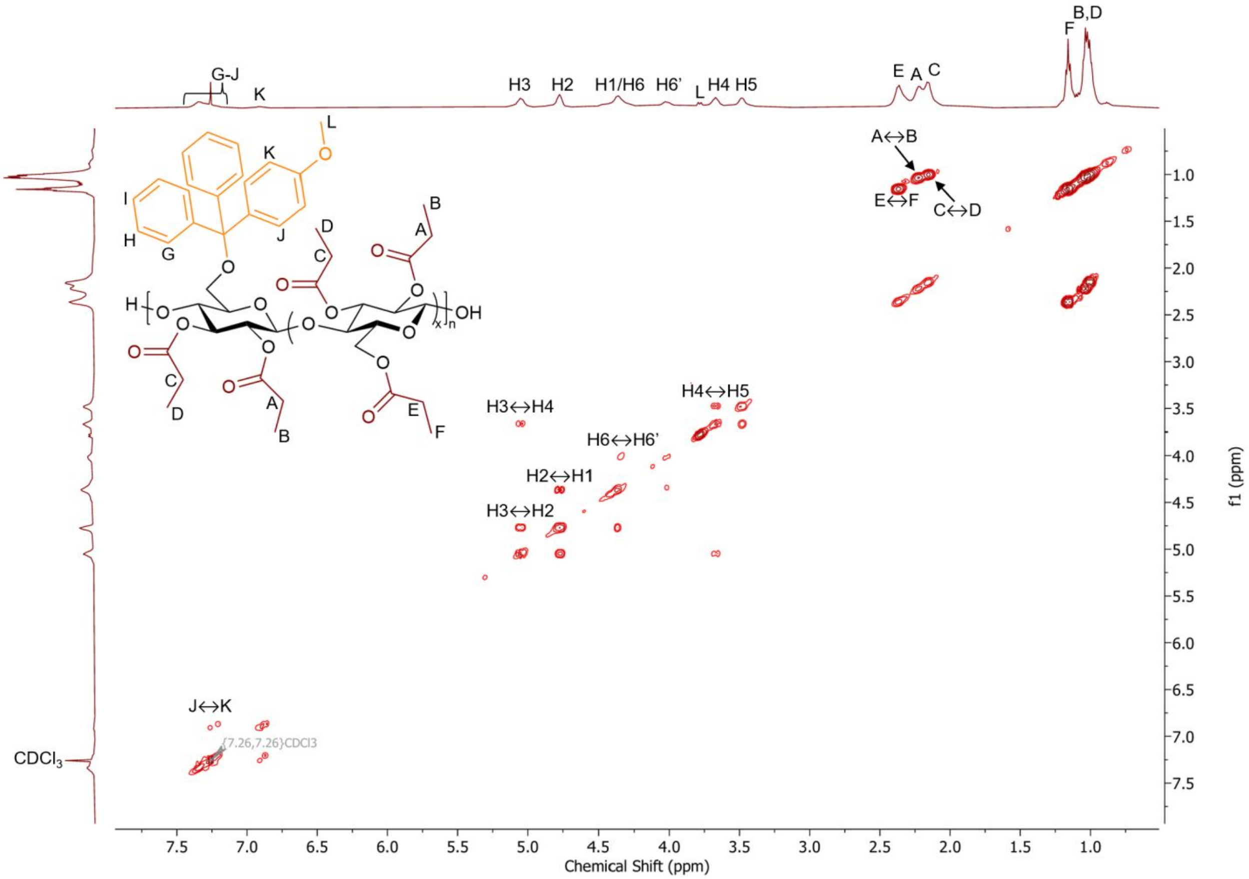

Figure S11: COSY spectrum of 2,3Pr-6MeOTr cellulose DS(Pr) 2.85 (G-MeOTr-Pr).

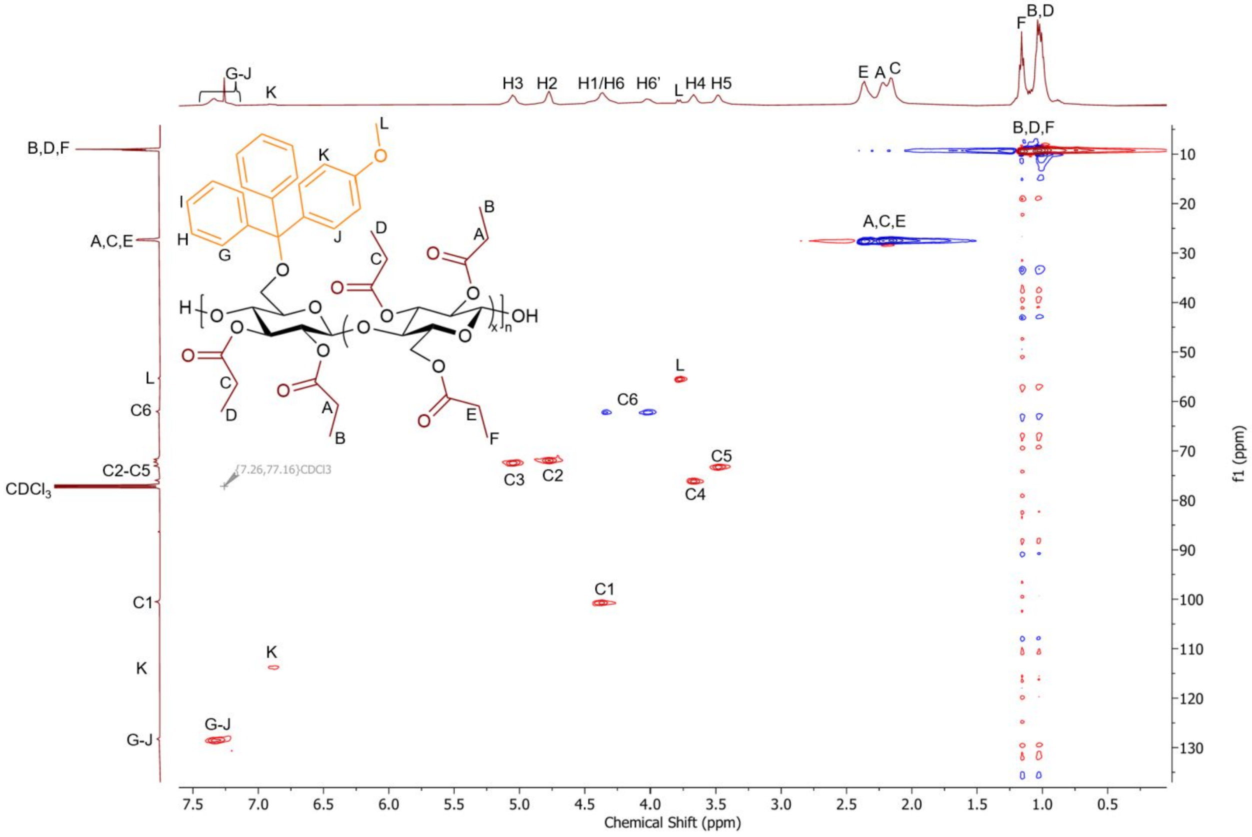

Figure S12: HSQC spectrum of 2,3Pr-6MeOTr cellulose DS(Pr) 2.85 (G-MeOTr-Pr).

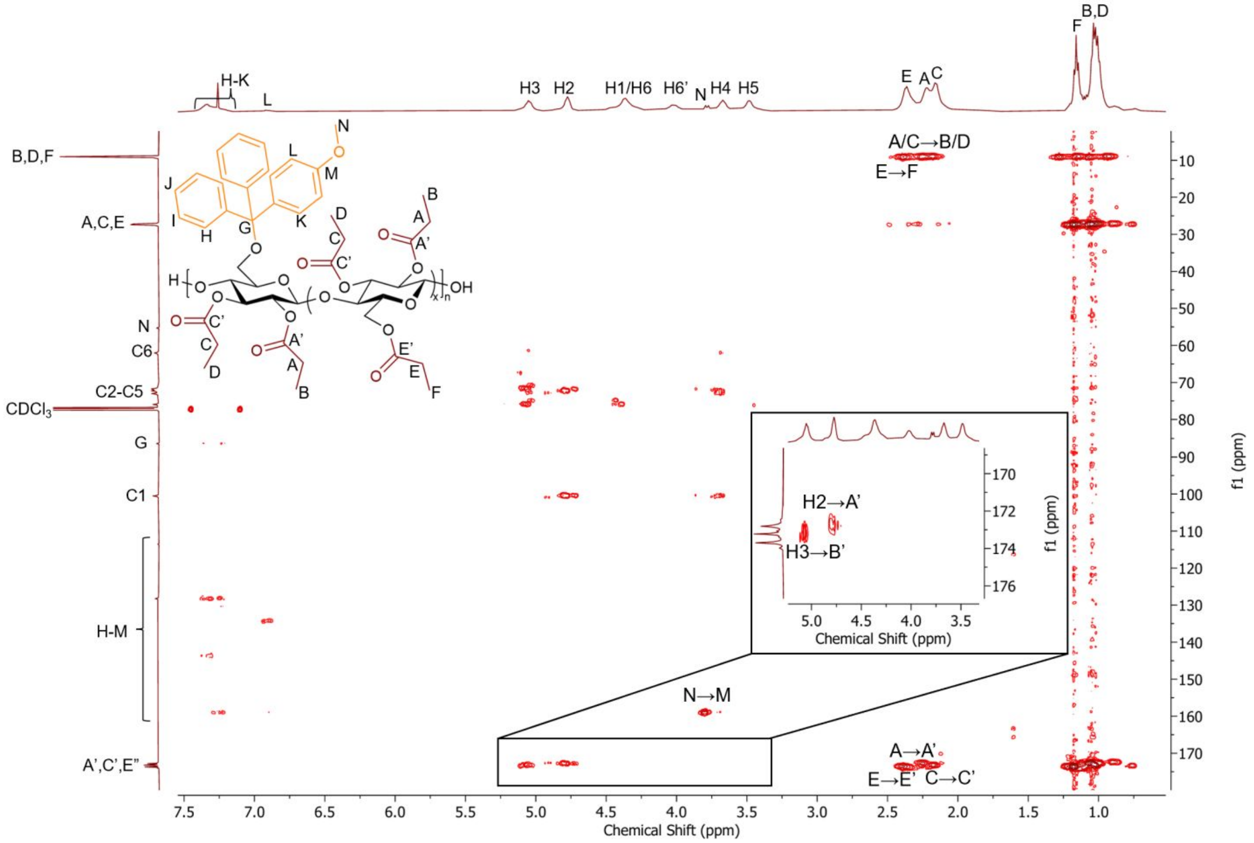

Figure S13: HMBC spectrum of 2,3Pr-6MeOTr cellulose DS(Pr) 2.85 (G-MeOTr-Pr).

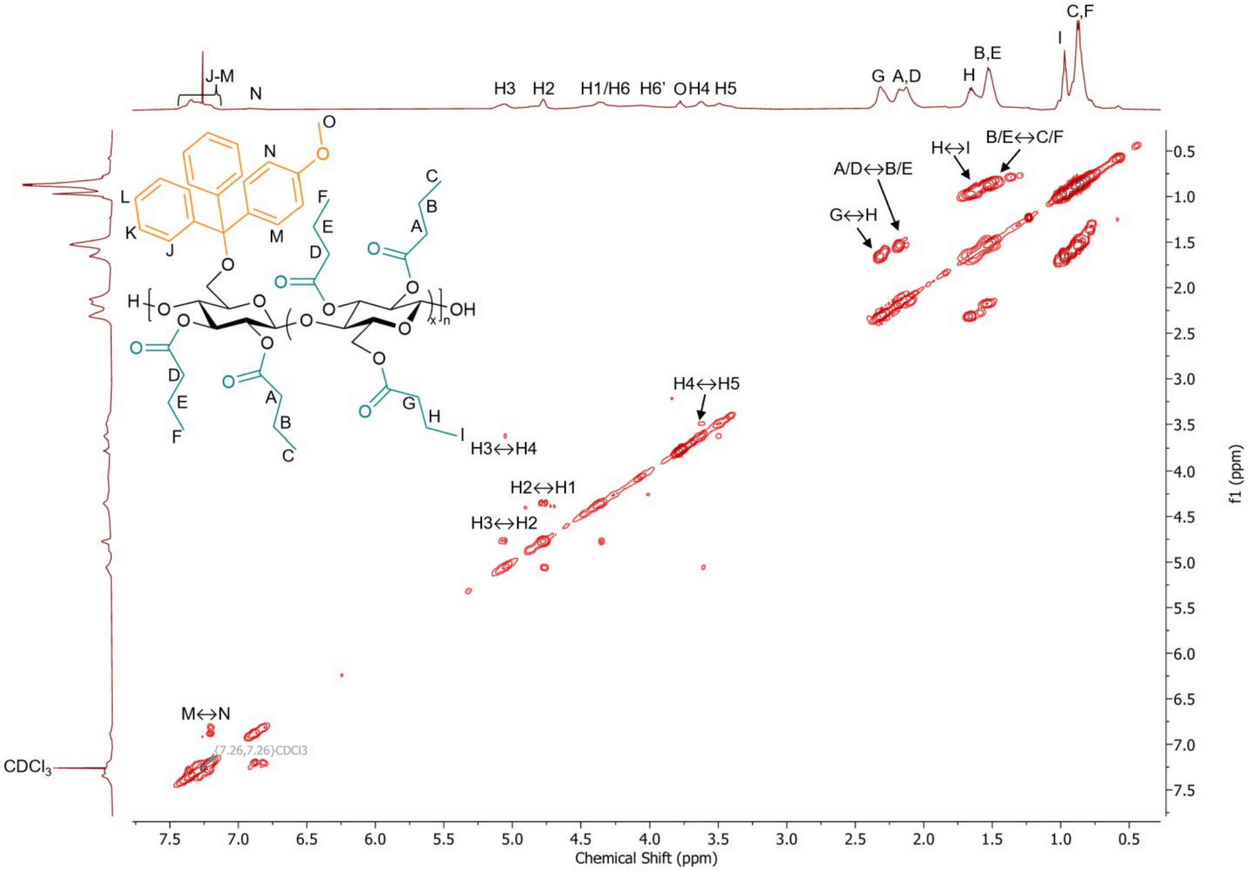

Figure S14: COSY Spectrum of 2,3Bu-6MeOTr cellulose DS(Bu) 2.72 (G-MeOTr-Bu).

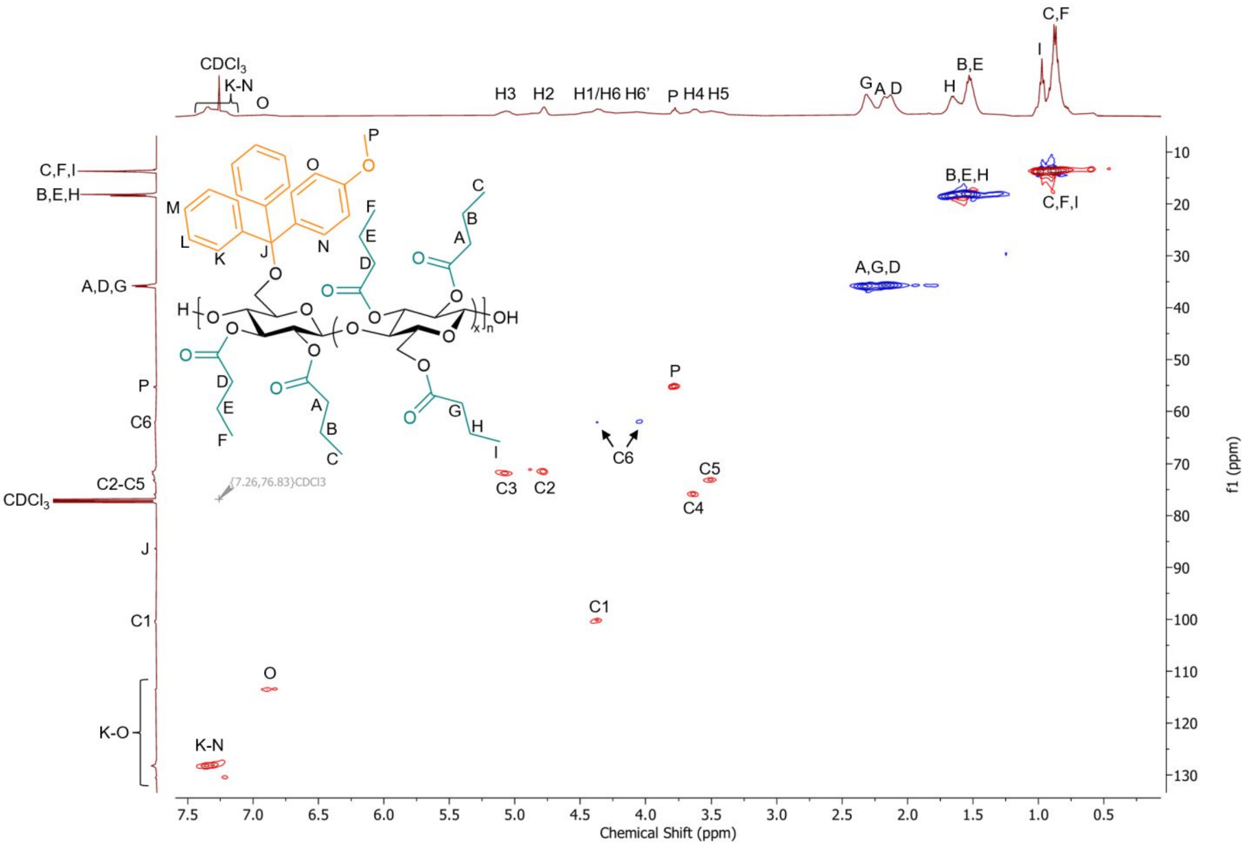

<u>**Figure S15:** HSQC spectrum of 2,3Bu-6MeOTr cellulose DS(Bu) 2.72 (G-MeOTr-Bu).</u>

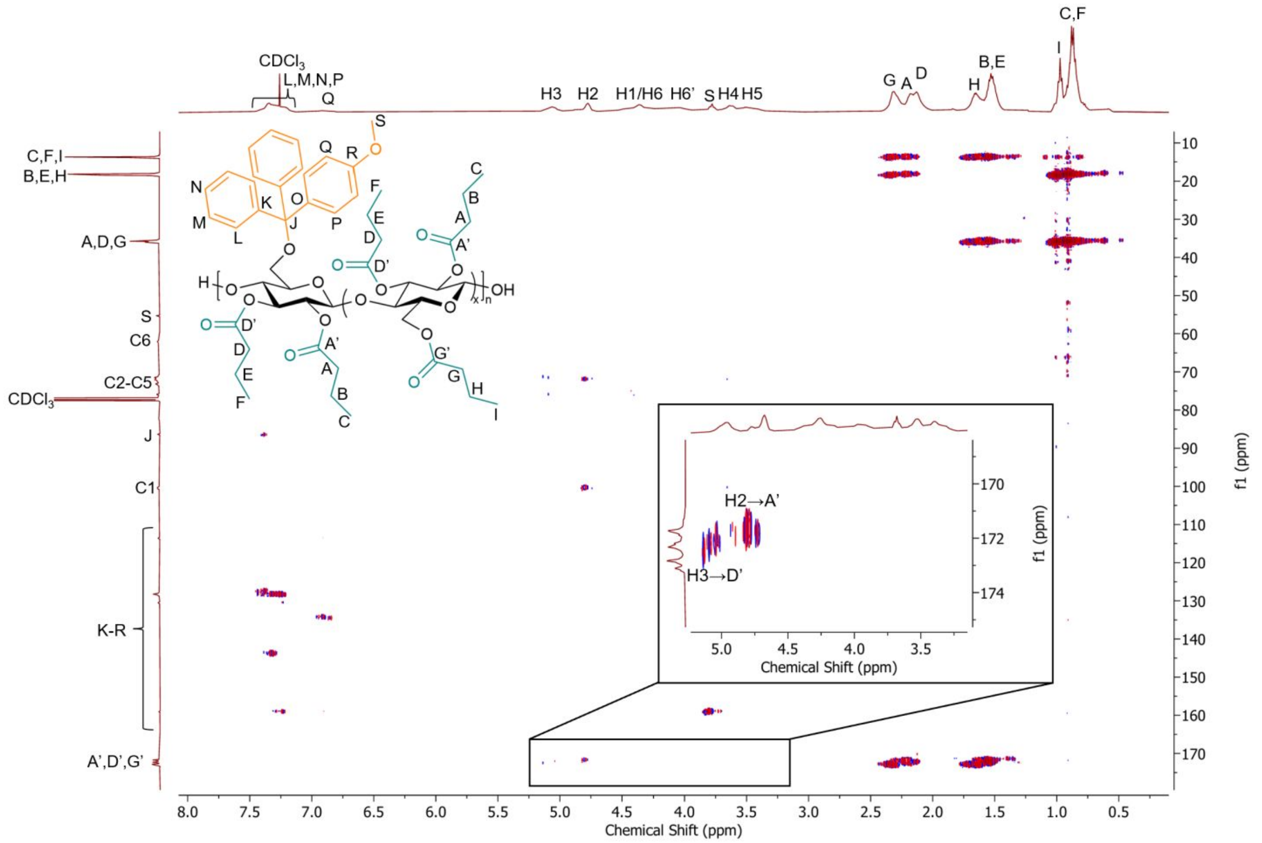

<u>**Figure S16:**</u> <u>HMBC spectrum of 2,3Bu-6MeOTr cellulose DS(Bu) 2.72 (G-MeOTr-Bu).</u>

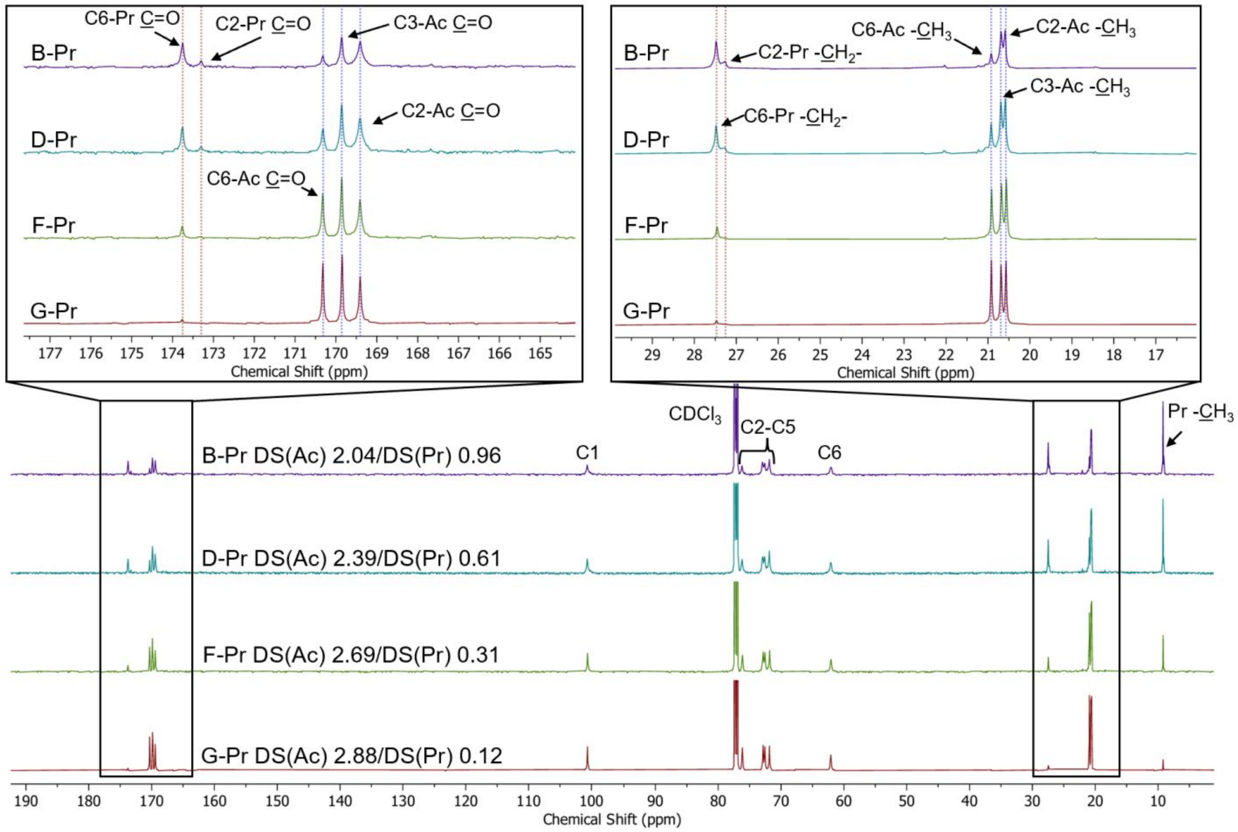

Figure S17: Stacked ^{13}C NMR spectra of 2,3Ac-6Pr celluloses with varying DS(Ac)/DS(Pr) (B-, D-, F-, and G-Pr).

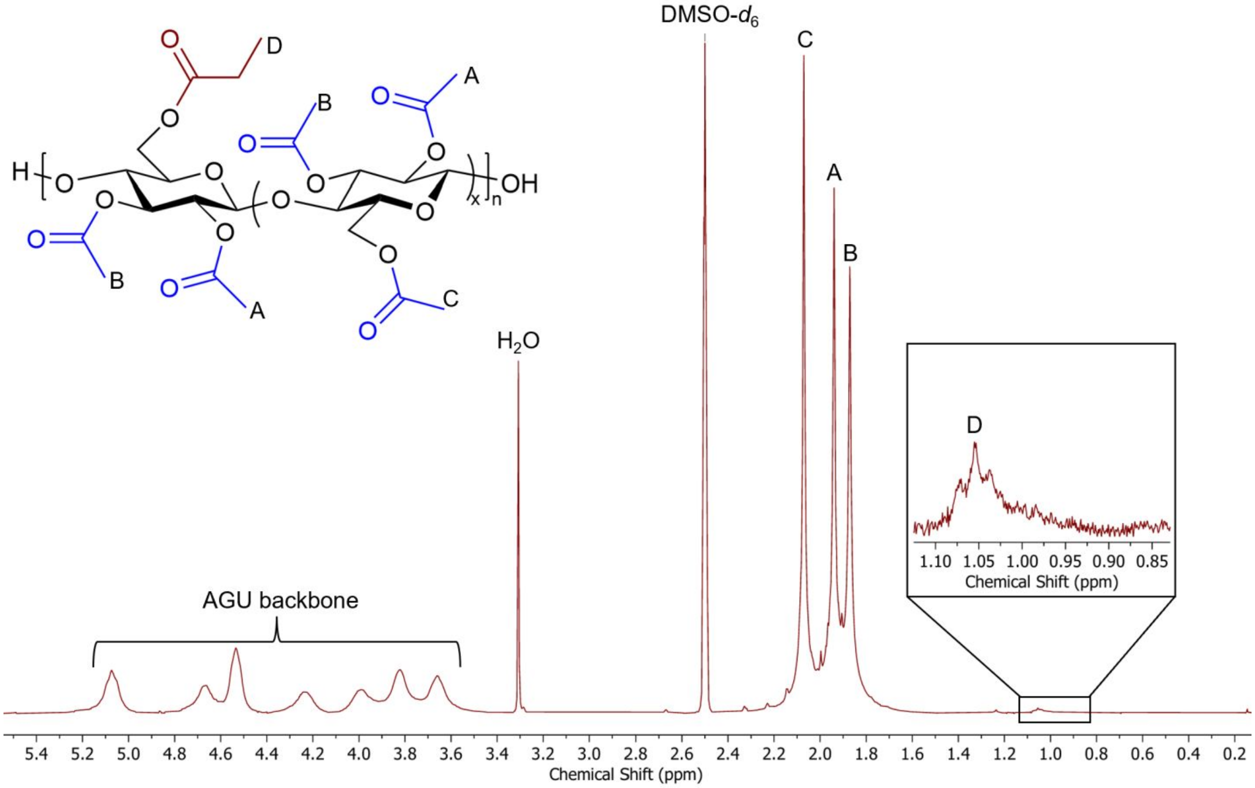

Figure S18: ^{1}H NMR spectrum of 2,3Ac-6Pr cellulose DS(Ac) 2.99 (H-Pr).

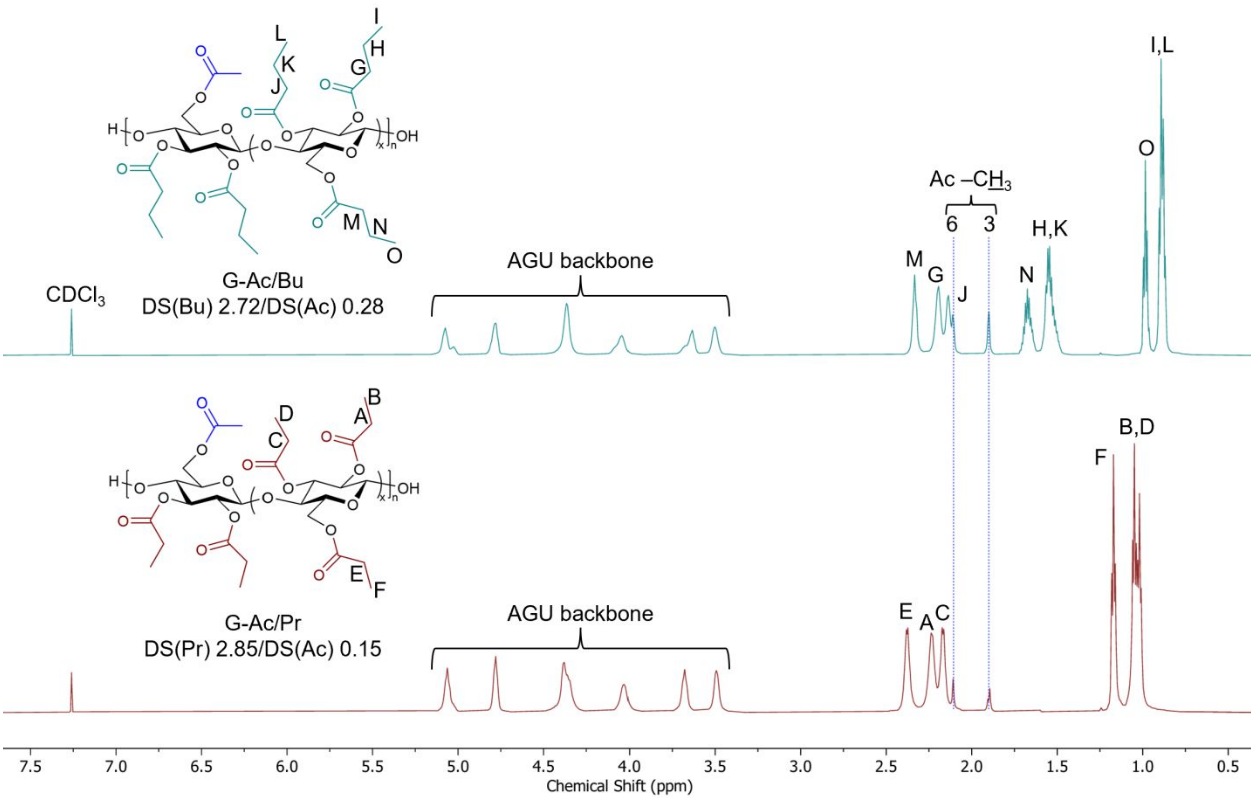

Figure S19: Stacked ^{1}H NMR spectra of 2,3Pr-6Ac cellulose DS(Pr) 2.85 (G-Ac/Pr) and 2,3Bu-6Ac cellulose DS(Bu) 2.72 (G-Ac/Bu).

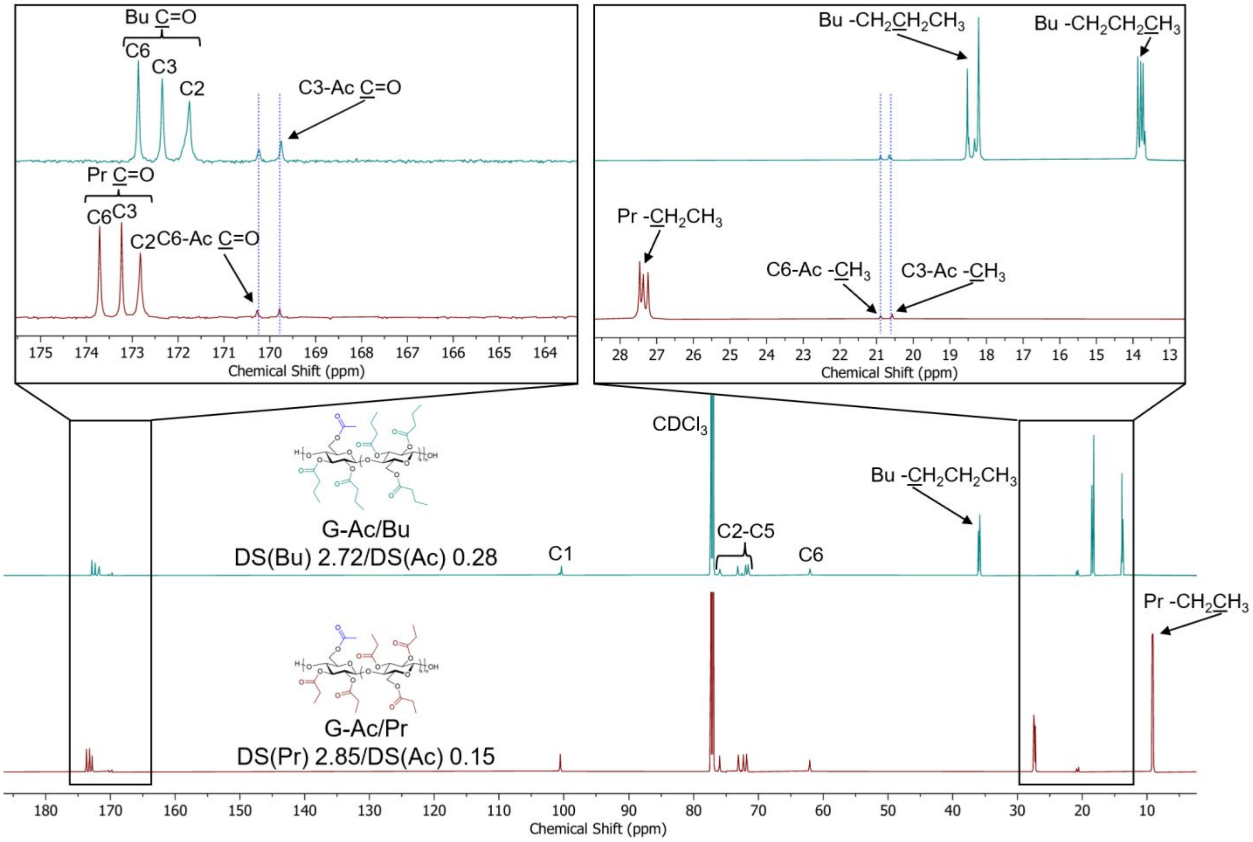

Figure S20: Stacked ^{13}C NMR spectra of 2,3Pr-6Ac cellulose DS(Pr) 2.85 (G-Ac/Pr) and 2,3Bu-6Ac cellulose DS(Bu) 2.72 (G-Ac/Bu).

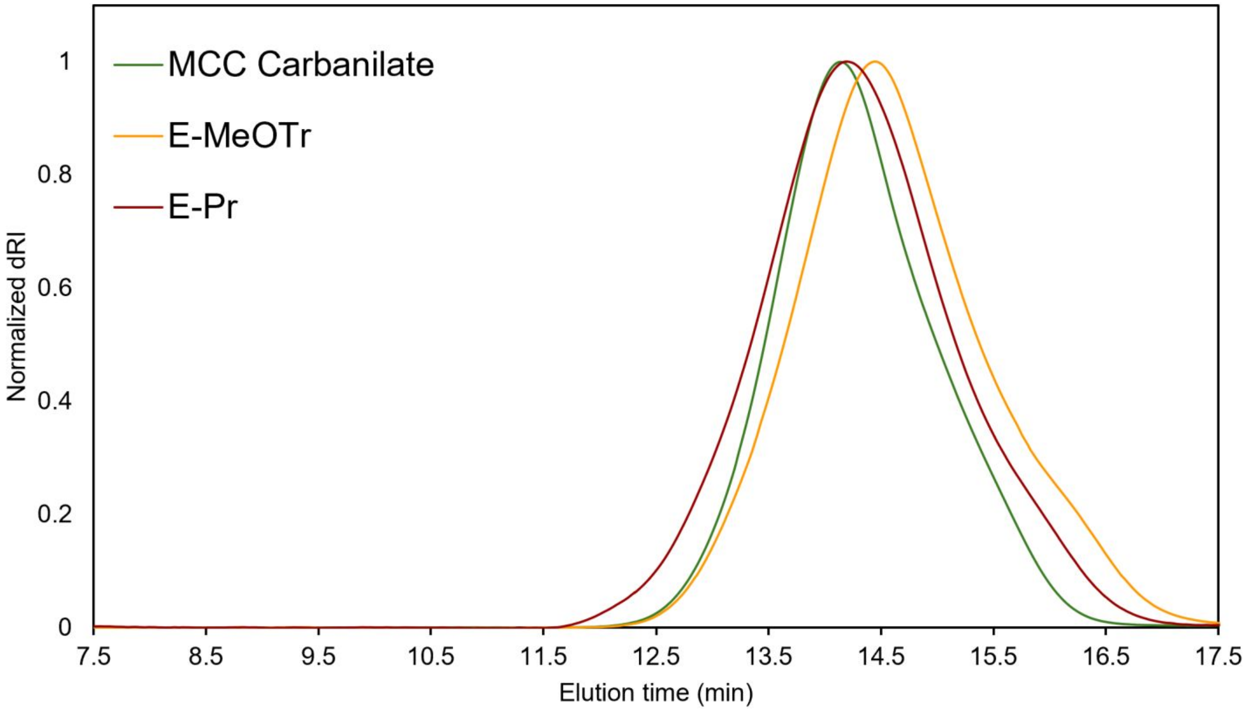

Figure S21: SEC chromatograms of carbanilated MCC, sample E-MeOTr, and sample E-Pr.

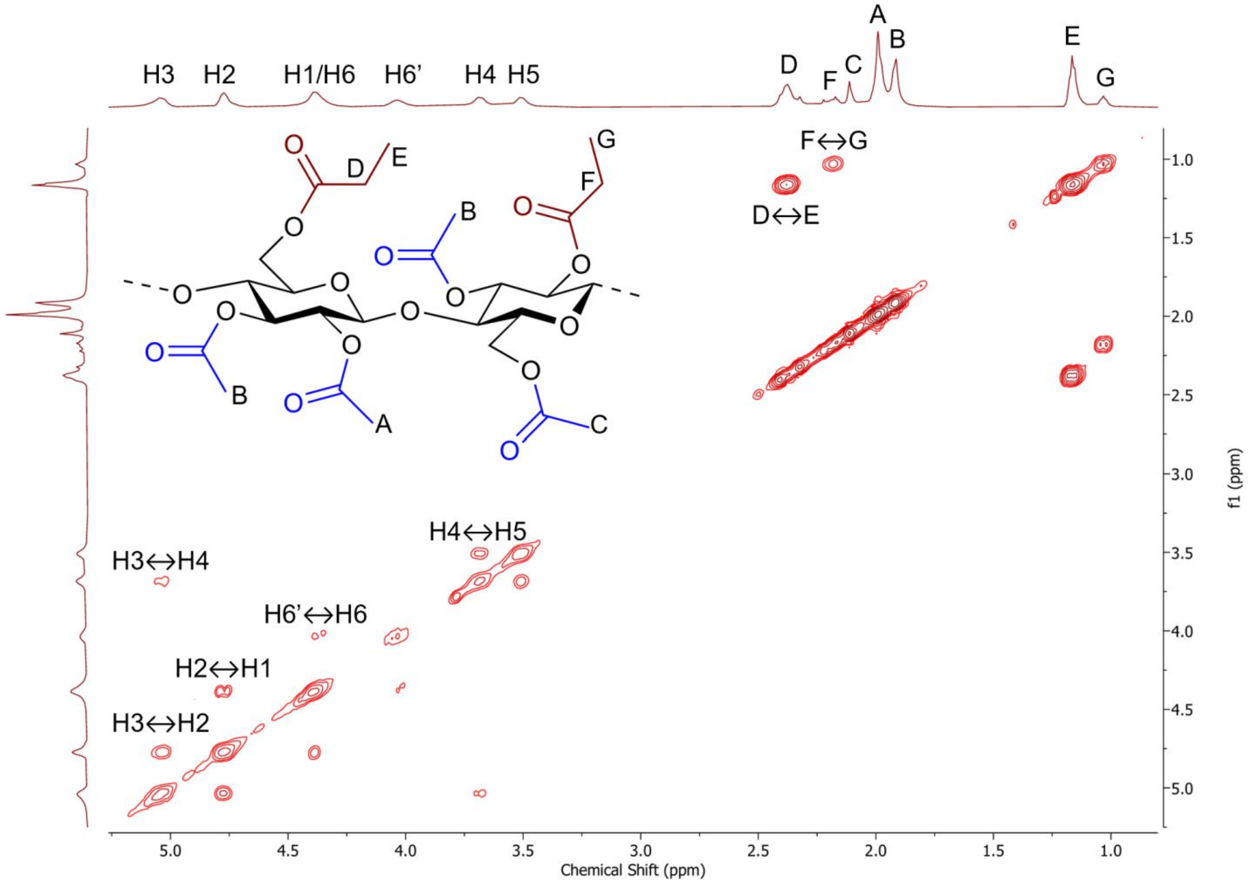

Figure S22: COSY spectrum of 2,3Ac-6Pr cellulose DS(Ac) 2.04 (B-Pr, $CDCl_3$).

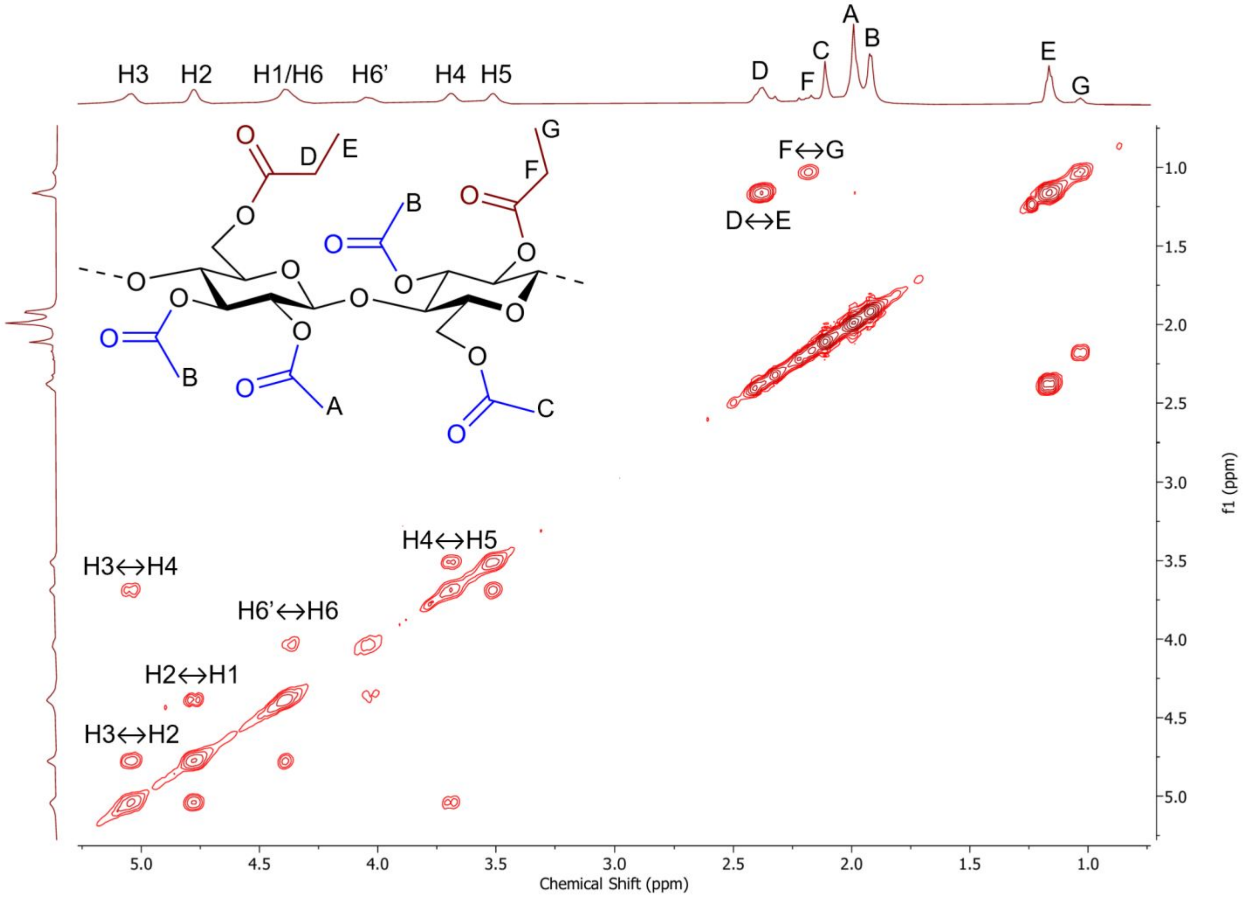

Figure S23: COSY spectrum of 2,3Ac-6Pr cellulose DS(Ac) 2.39 (D-Pr, $CDCl_3$).

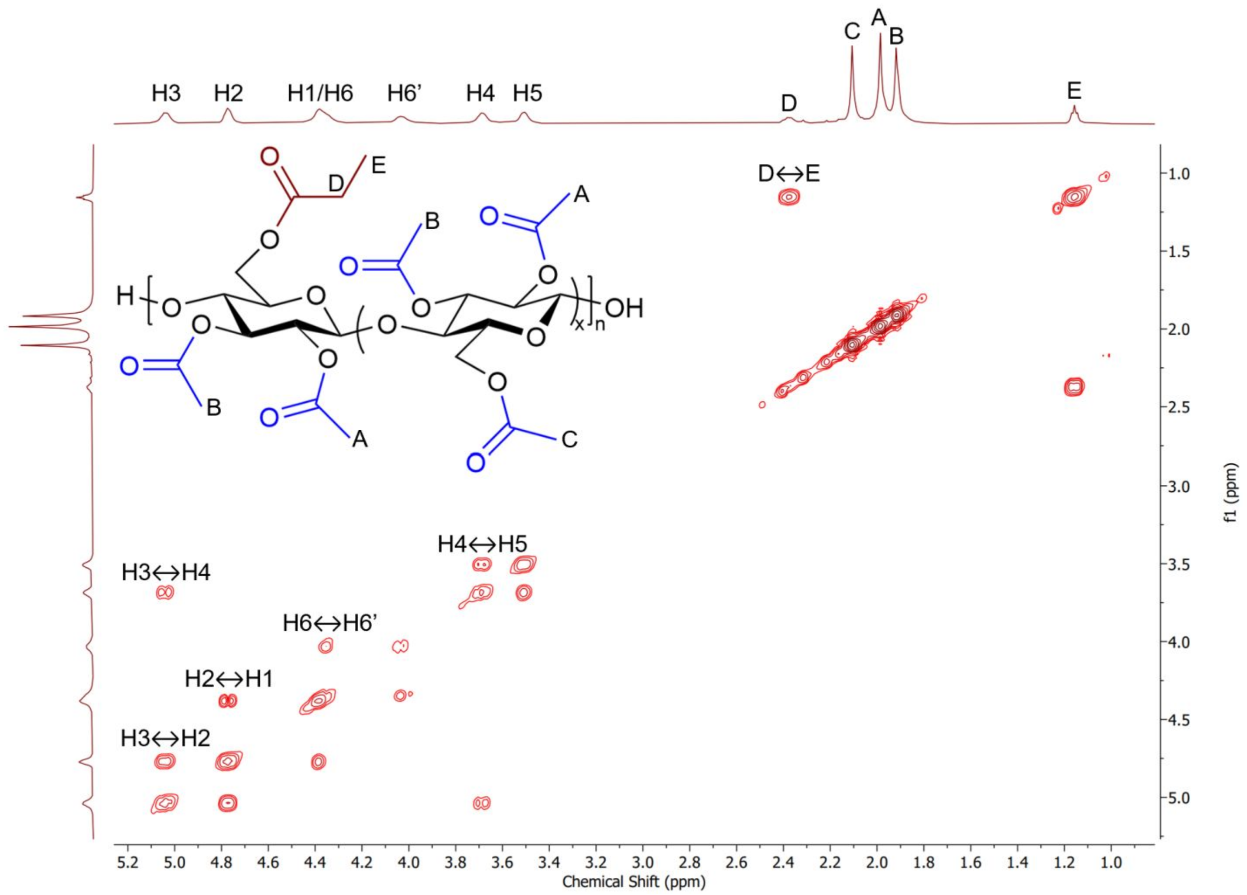

Figure S24: COSY spectrum of 2,3Ac-6Pr cellulose DS(Ac) 2.69 (F-Pr, $CDCl_3$).

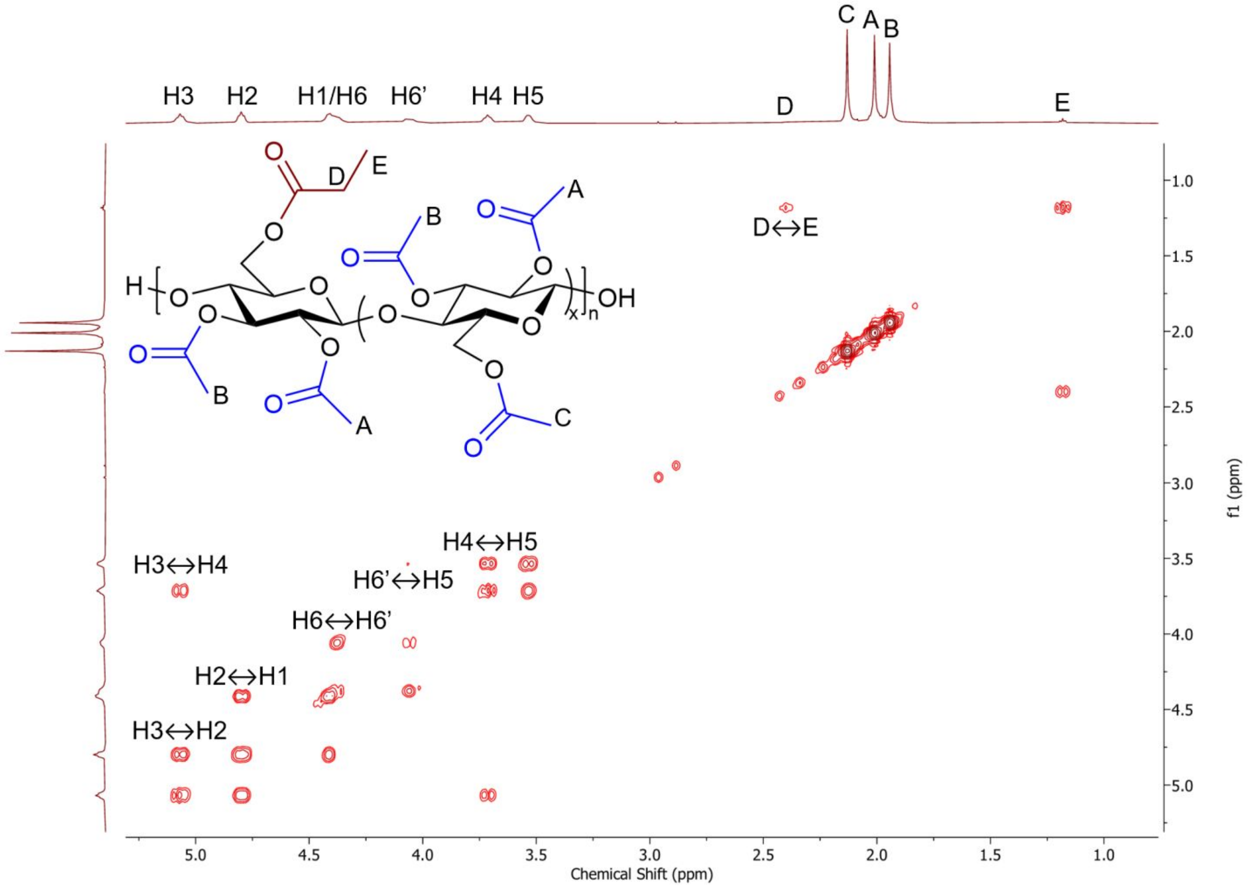

Figure S25: COSY spectrum of 2,3Ac-6Pr cellulose DS(Ac) 2.88 (G-Pr, $CDCl_3$).

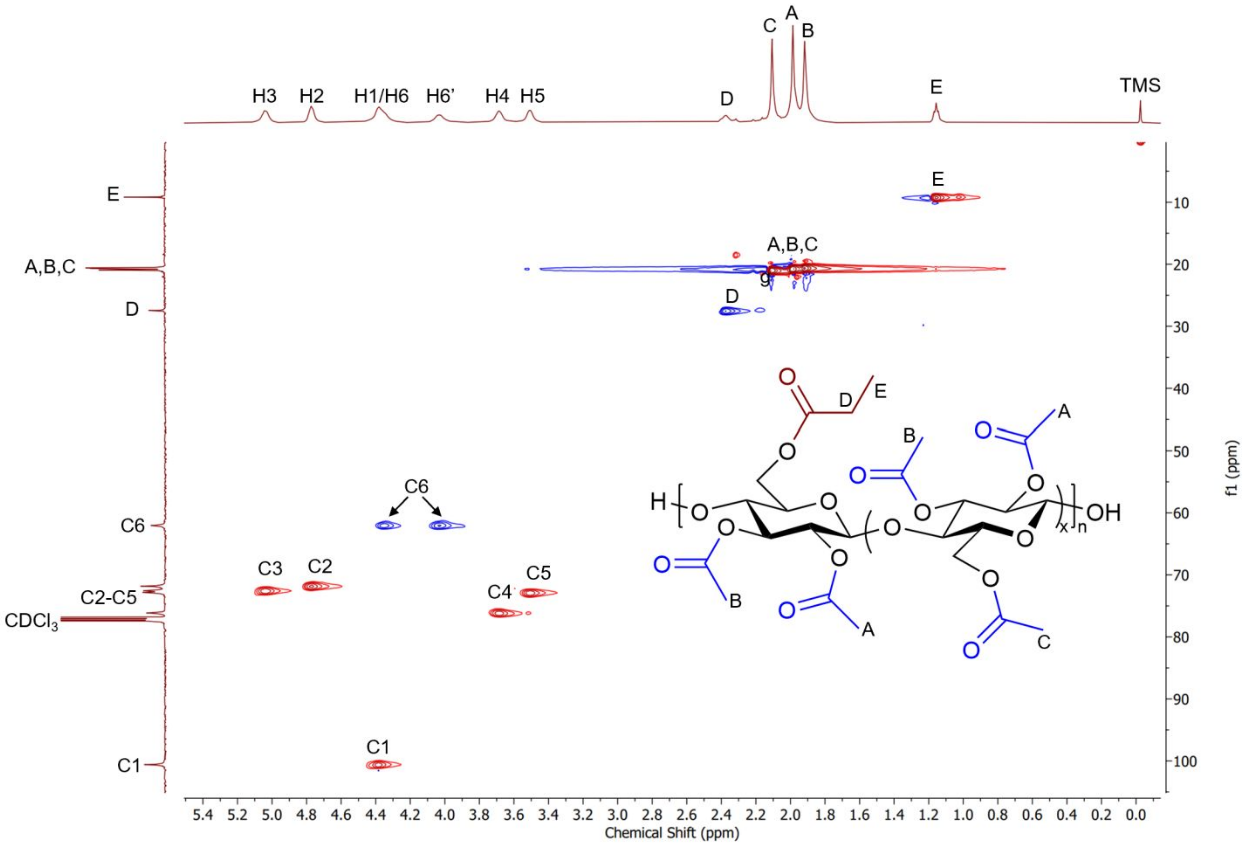

Figure S26: HSQC spectrum of 2,3Ac-6Pr cellulose DS(Ac) 2.69 (F-Pr).

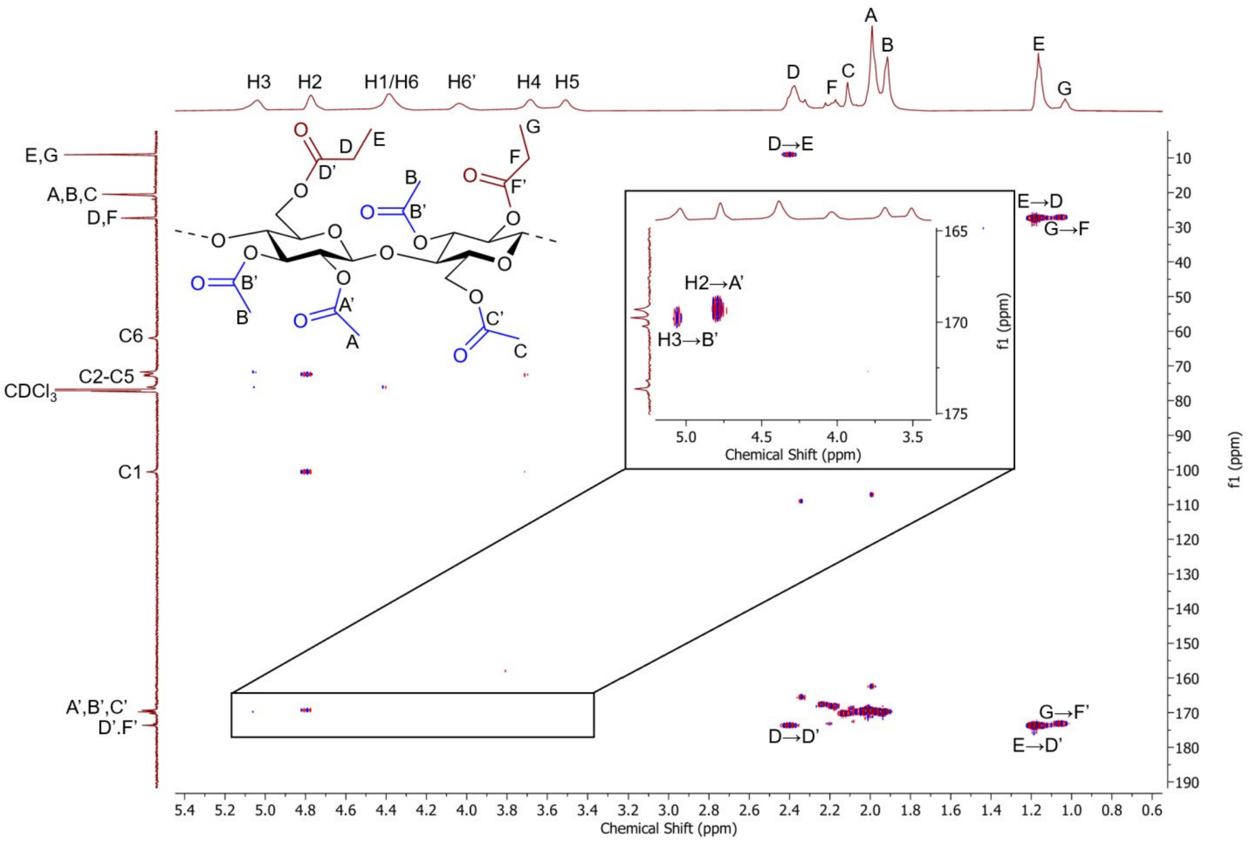

Figure S27: HMBC spectrum of 2,3Ac-6Pr cellulose DS(Ac) 2.04 (B-Pr).

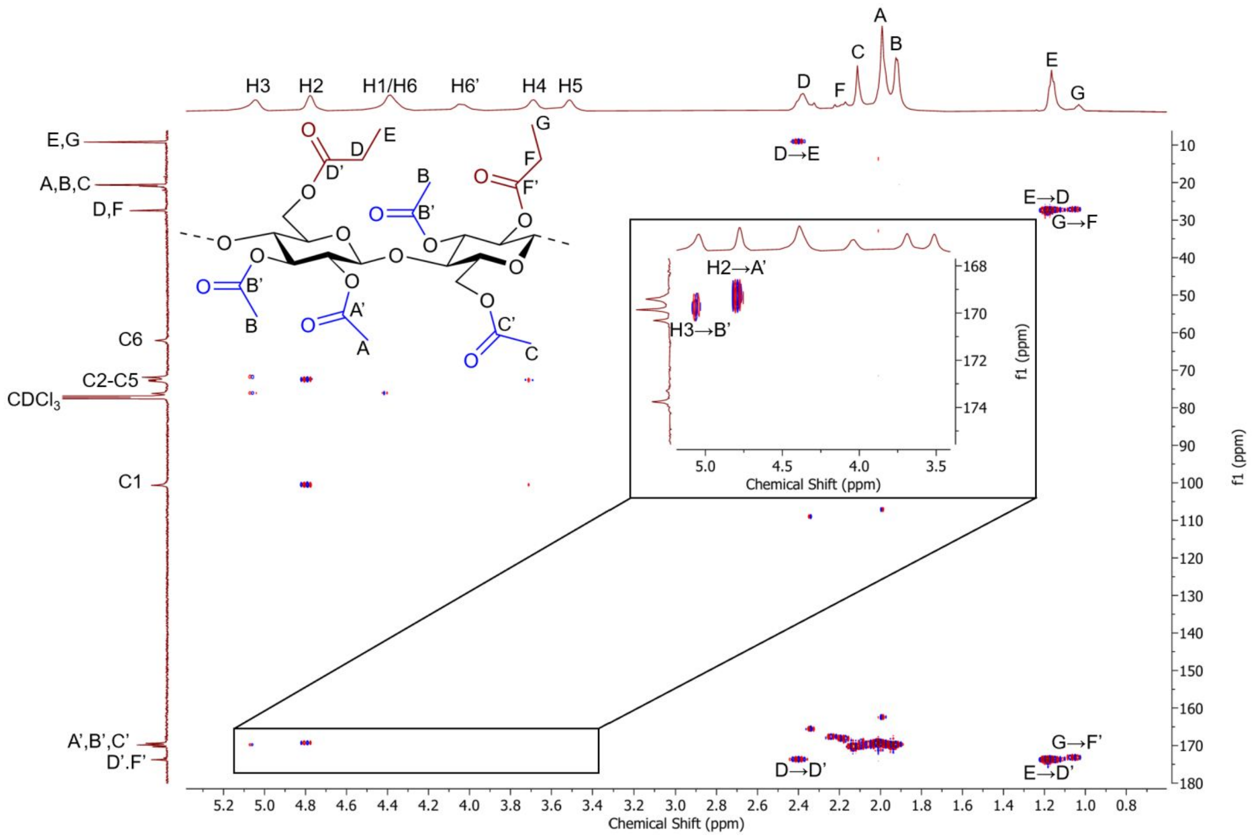

Figure S28: HMBC spectrum of 2,3Ac-6Pr cellulose DS(Ac) 2.34 (D-Pr).

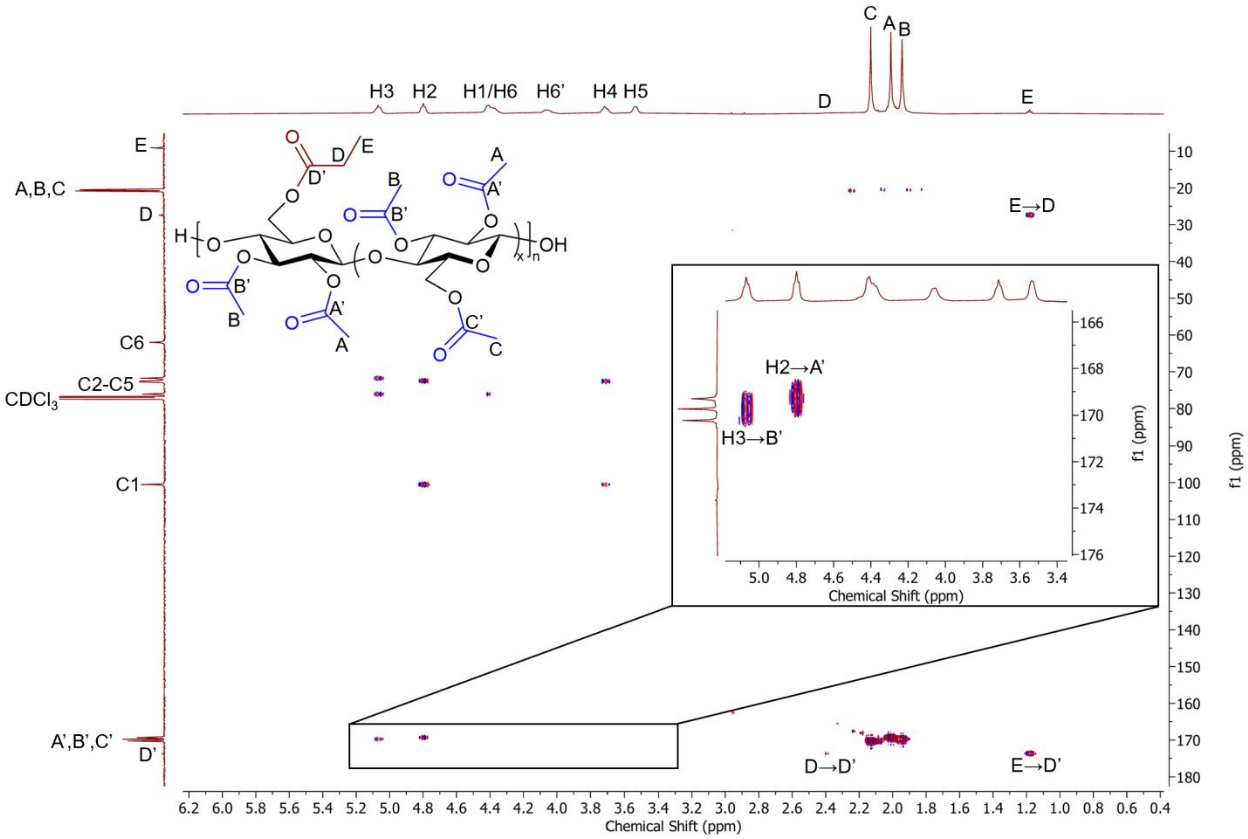

<u>**Figure S29:** HMBC spectrum of 2,3Ac-6Pr cellulose DS(Ac) 2.88 (G-Pr).</u>

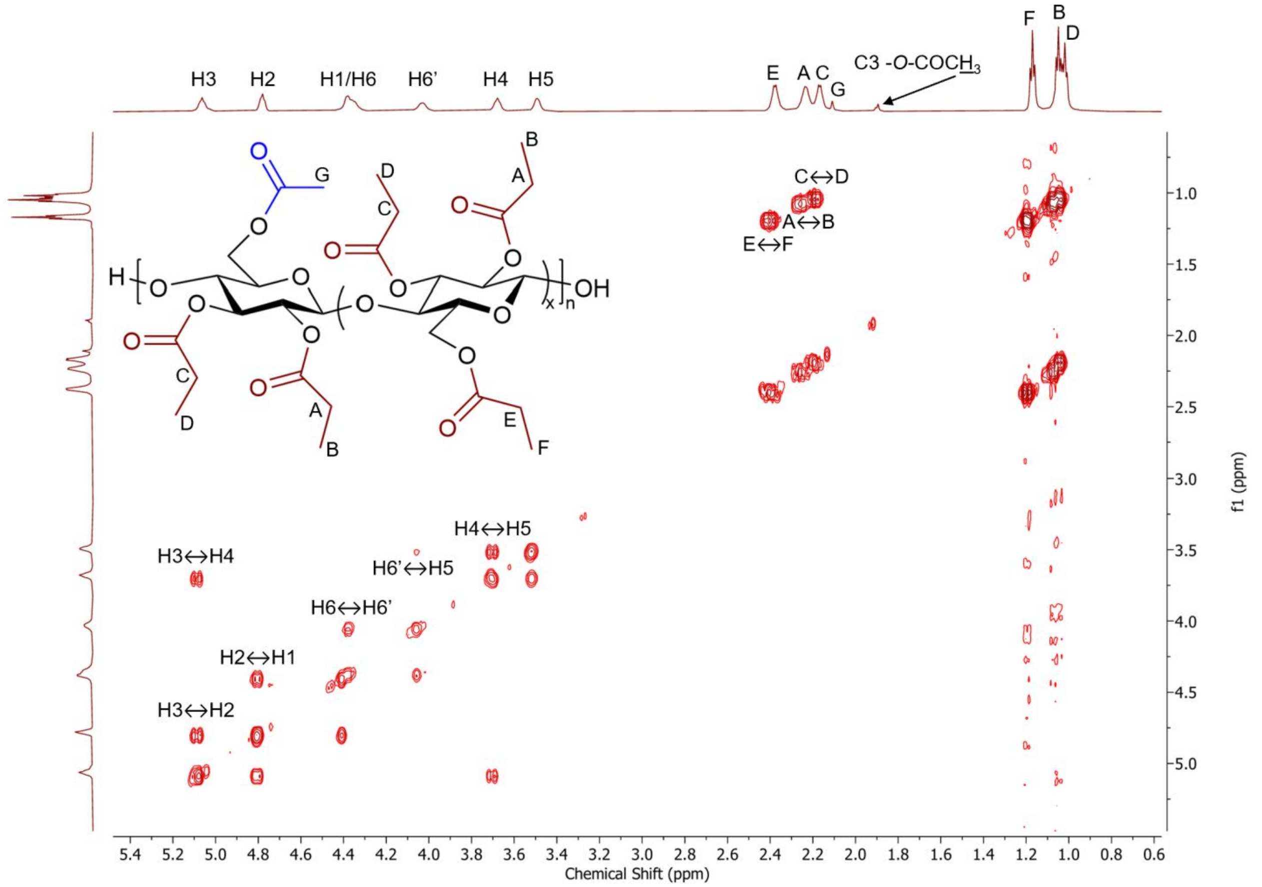

Figure S30: COSY spectrum of 2,3Pr-6Ac cellulose DS(Pr) 2.85 (G-Ac/Pr, $CDCl_3$).

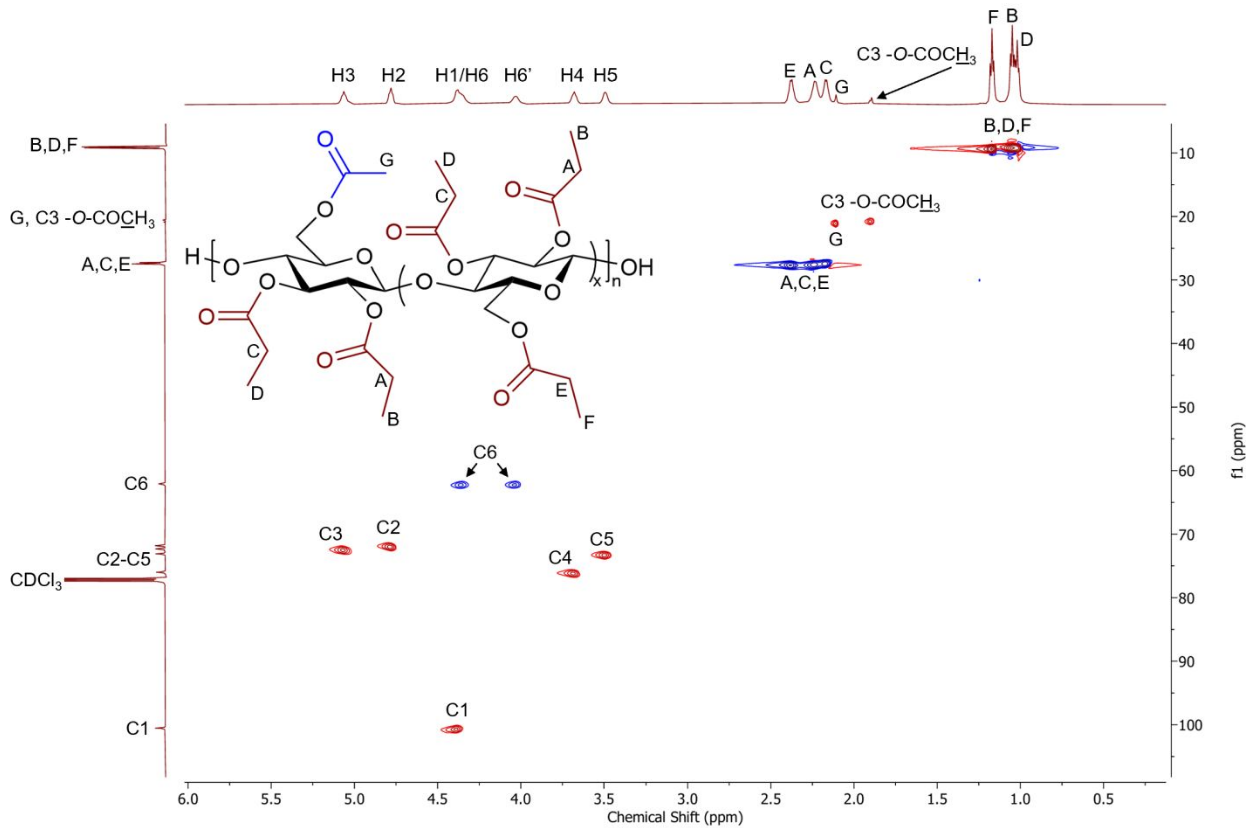

Figure S31: HSQC spectrum of 2,3Pr-6Ac cellulose DS(Pr) 2.85 (G-Ac/Pr).

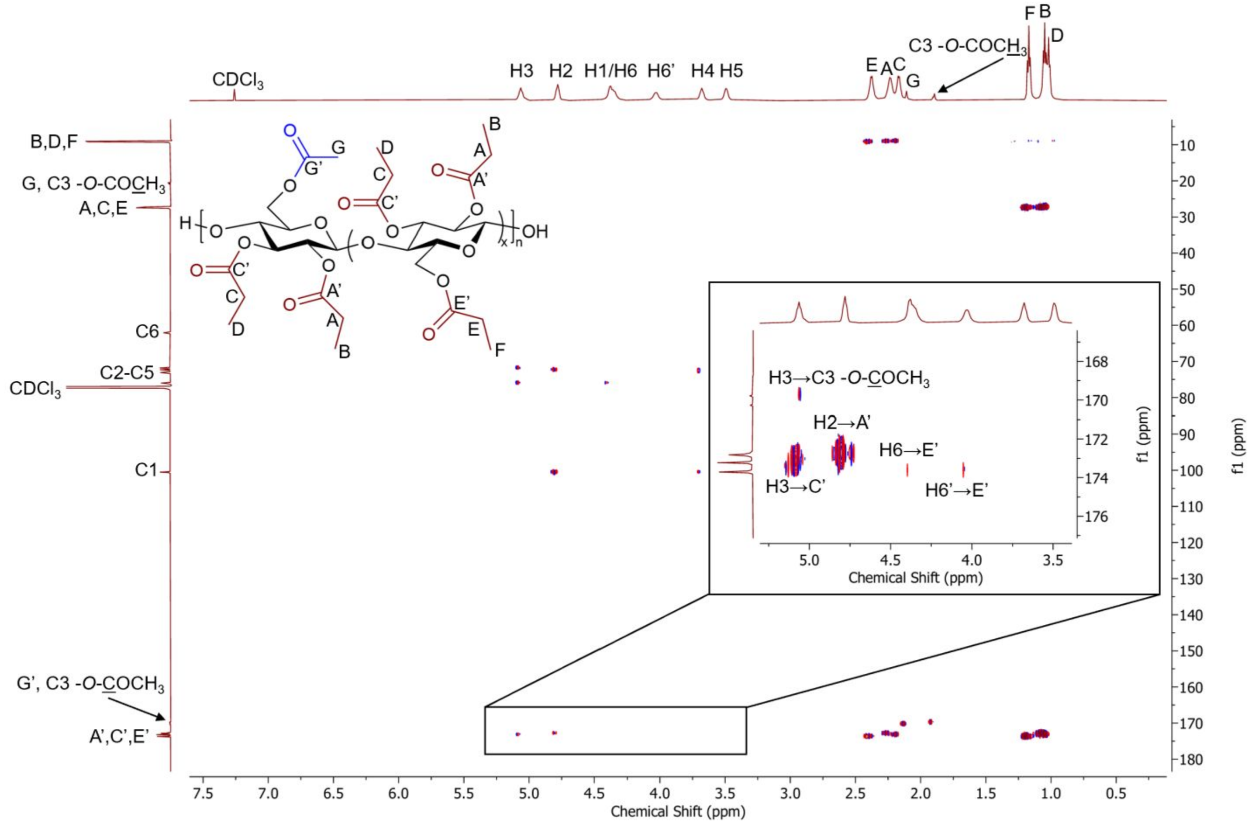

Figure S32: HMBC spectrum of 2,3Pr-6Ac cellulose DS(Pr) 2.85 (G-Ac/Pr).

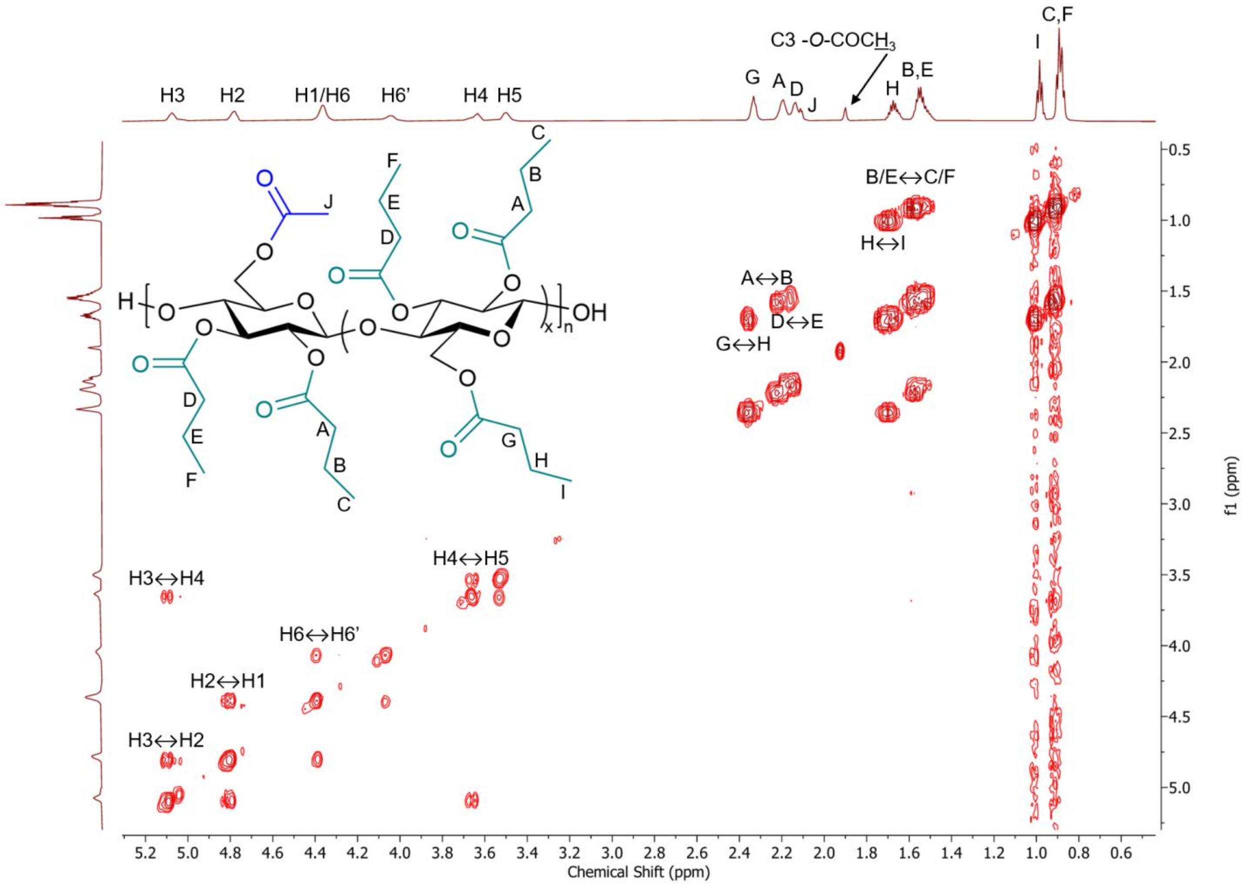

Figure S33: COSY spectrum of 2,3Bu-6Ac cellulose DS(Bu) 2.72 (G-Ac/Bu, $CDCl_3$).

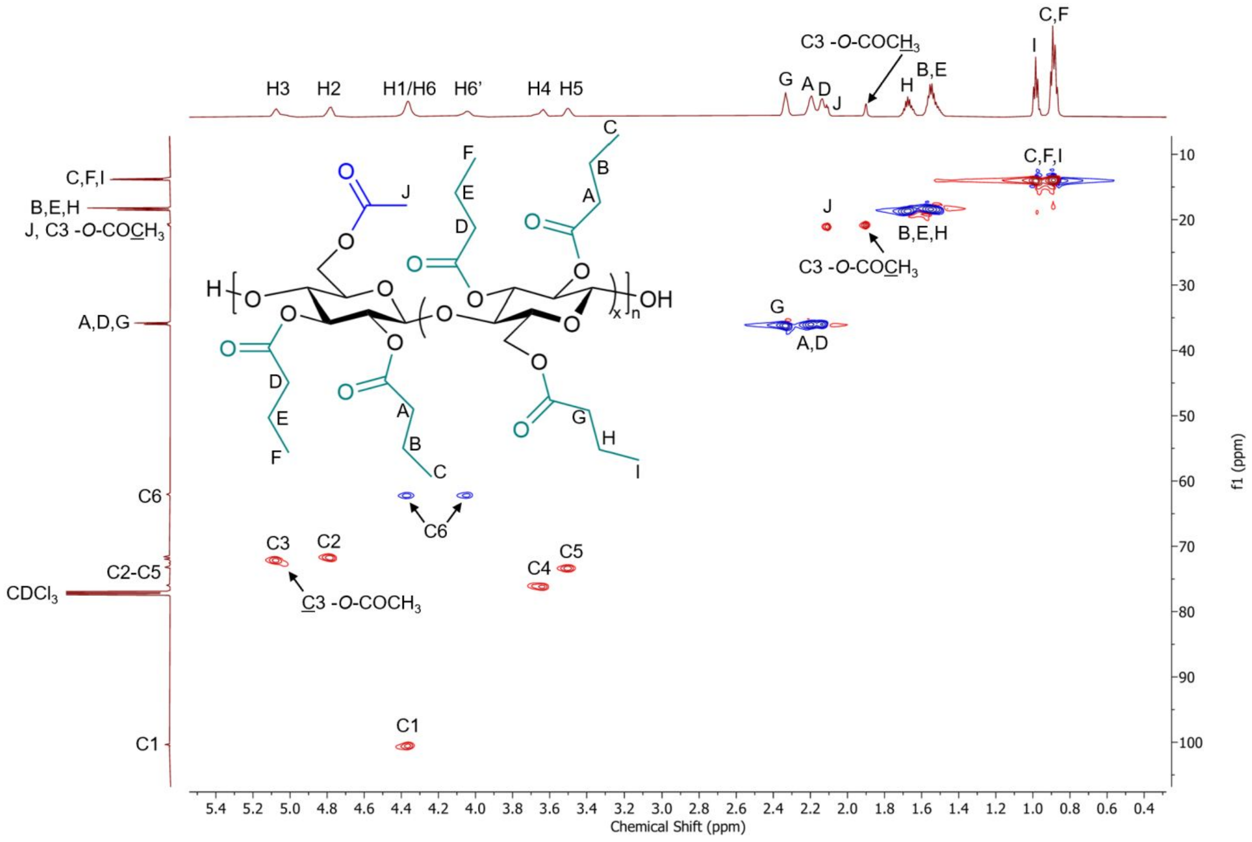

Figure S34: HSQC spectrum of 2,3Bu-6Ac cellulose DS(Bu) 2.72 (G-Ac/Bu).

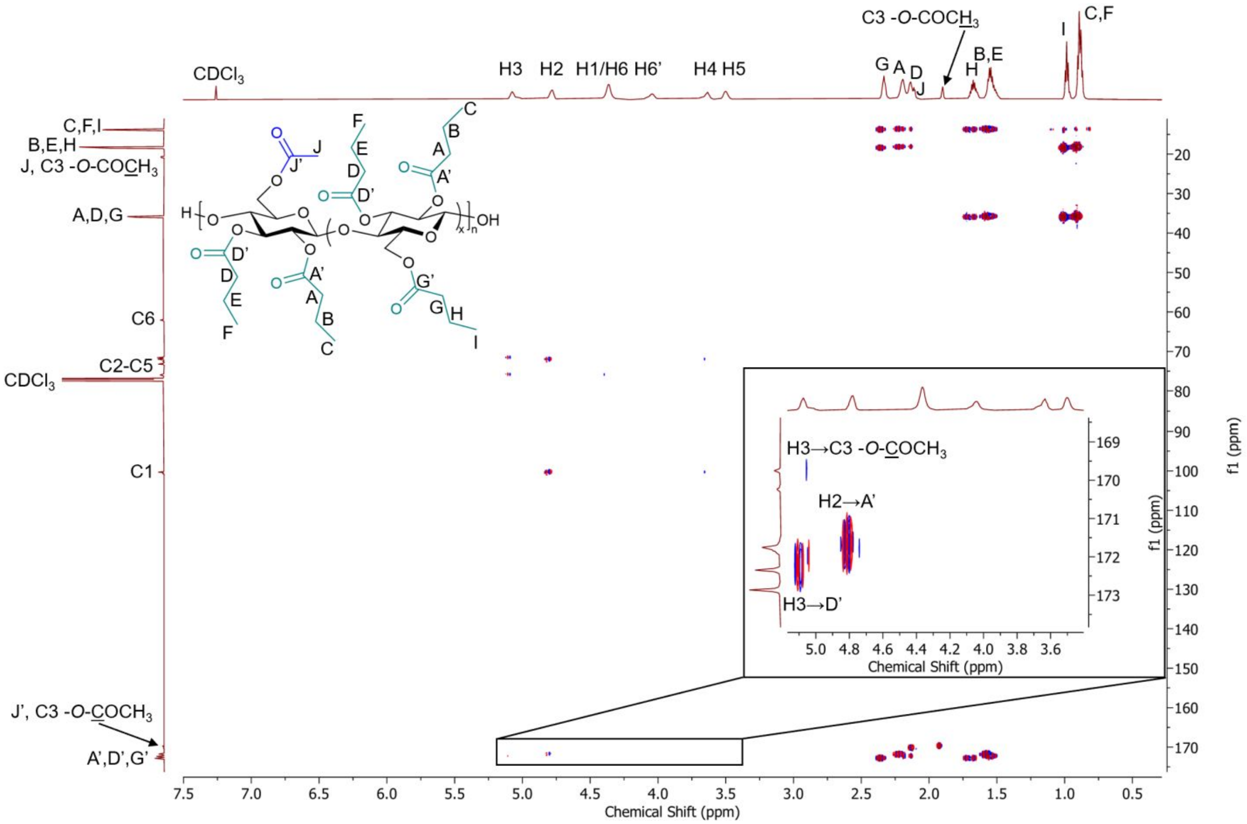

Figure S35: HMBC spectrum of 2,3Bu-6Ac cellulose DS(Bu) 2.72 (G-Ac/Bu).

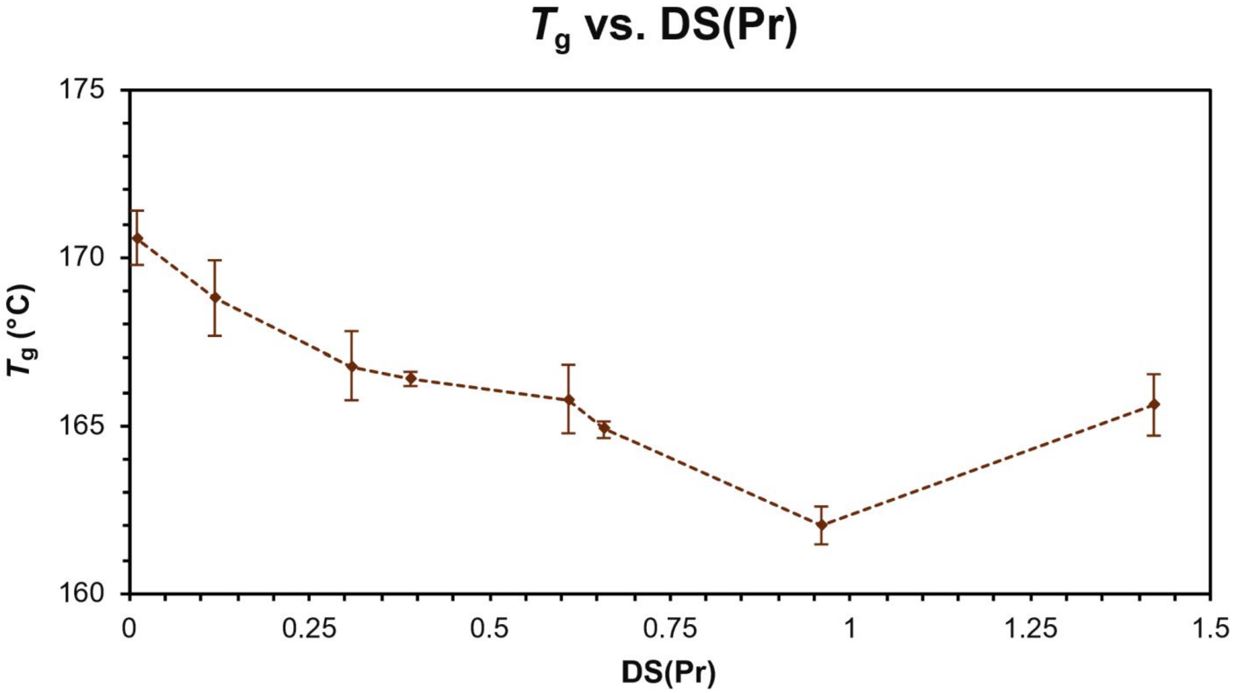

Figure S36: Plot of T_g versus DS(Pr) for 2,3Ac-6Pr cellulose esters.

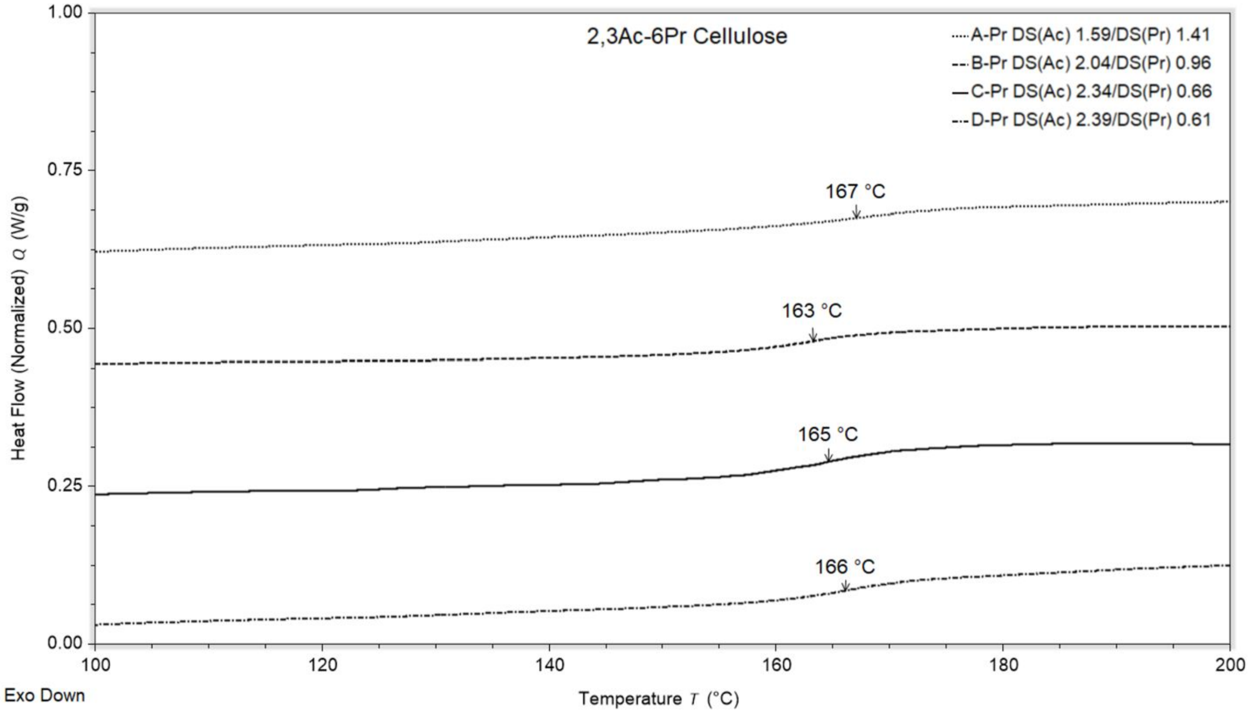

Figure S37: Representative DSC thermograms of A–D-Pr.

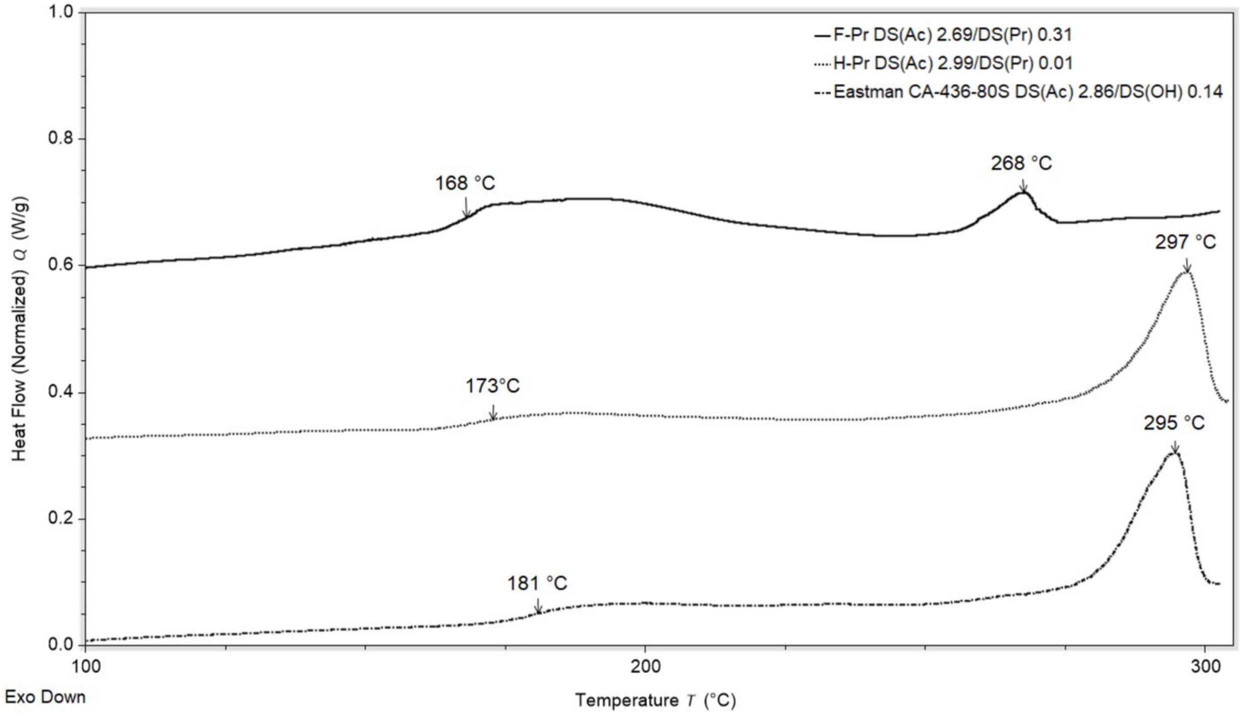

Figure S38: Representative DSC thermograms of F-Pr, H-Pr, and Eastman CA-436-80S.

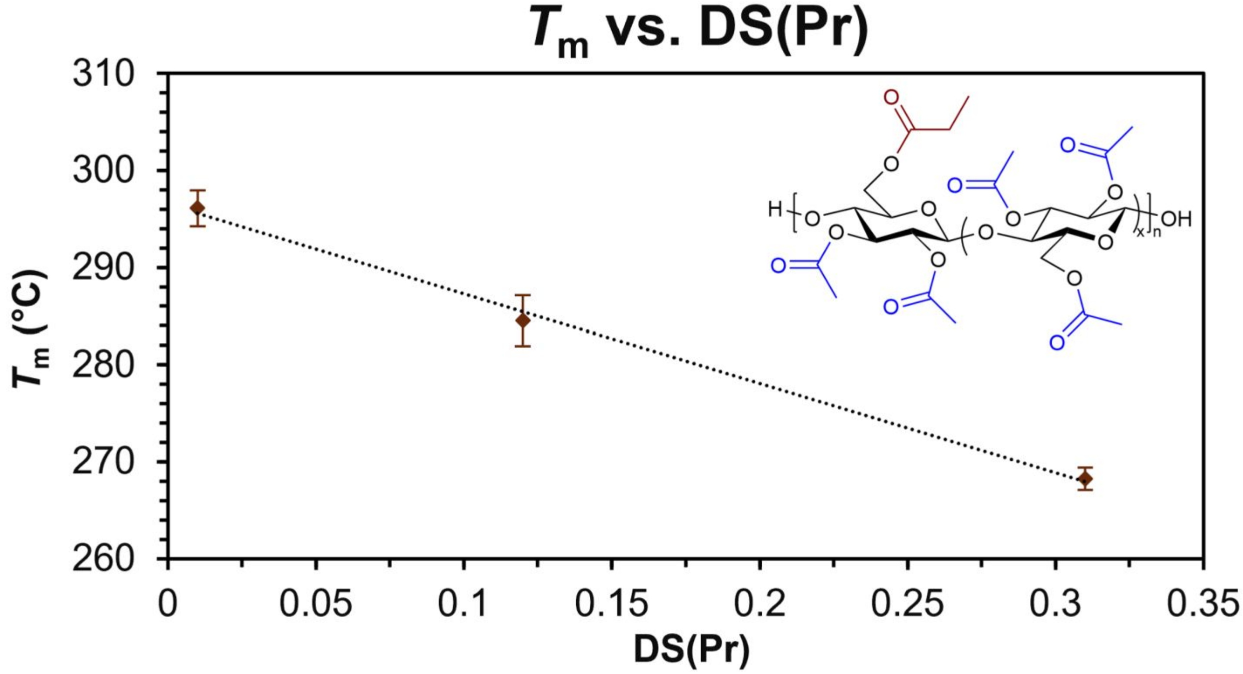

Figure S39: Plot of T_m vs DS(Pr) for semicrystalline 2,3Ac-6Pr cellulose esters.

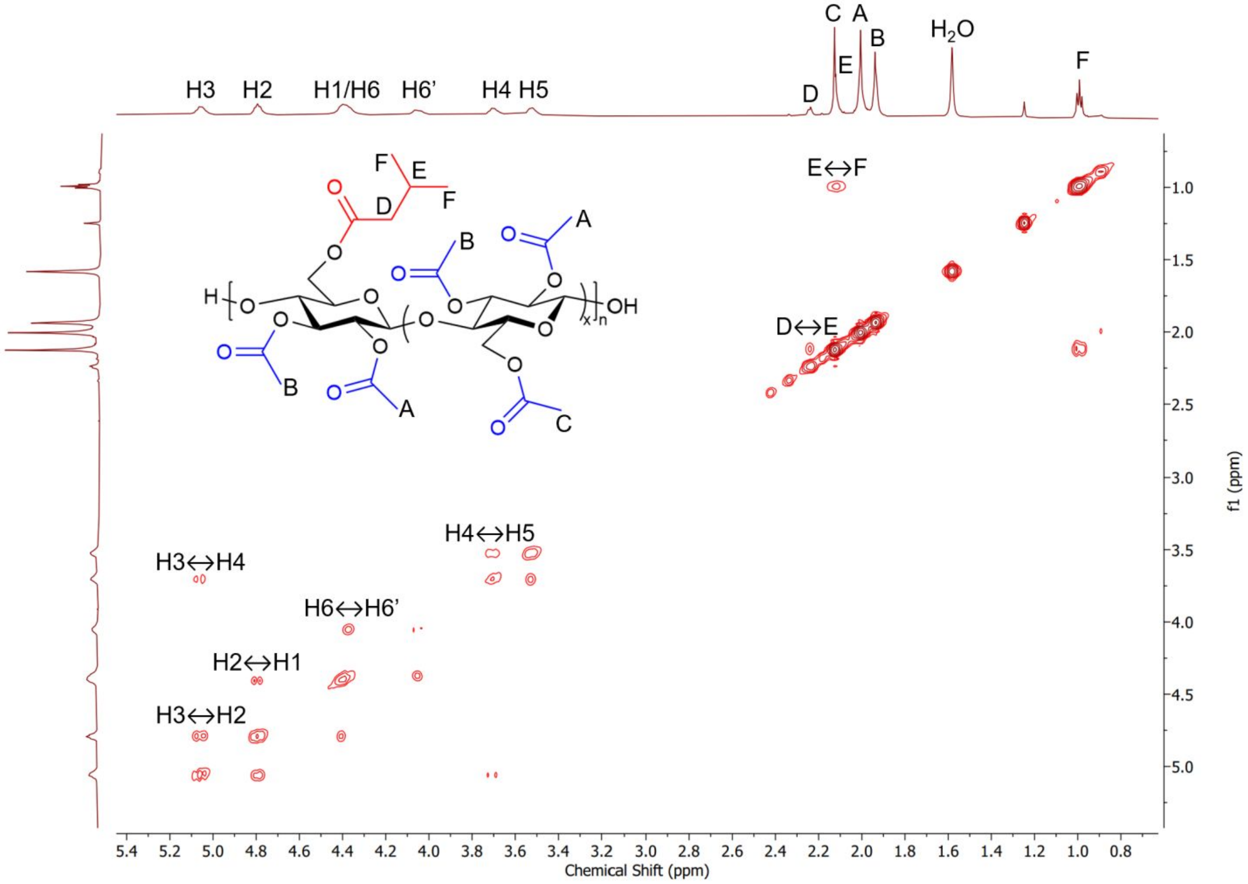

Figure S40: COSY spectrum of 2,3Ac-6*i*Va cellulose DS(Ac) 2.69 (F-*i*Va, $CDCl_3$).

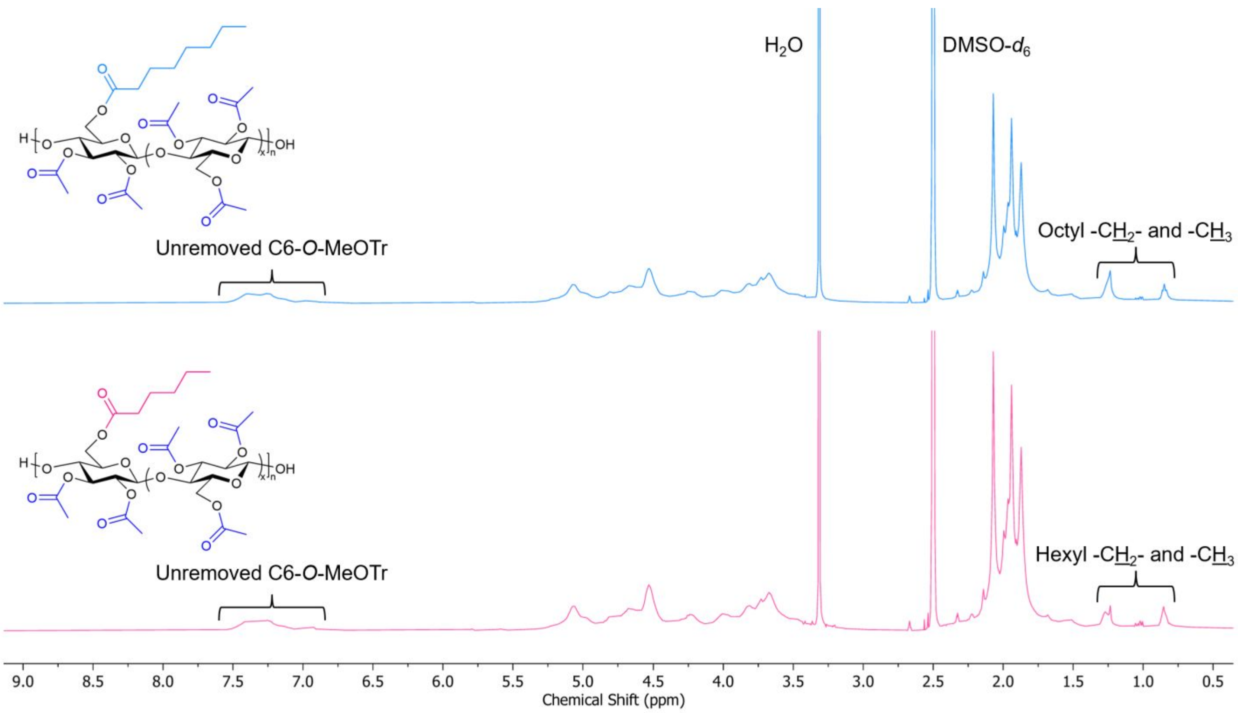

Figure S41: Stacked ^{1}H NMR spectra of 2,3Ac-6Hex and 2,3Ac-6Oct cellulose DS(Ac) 2.61 with remaining C6-*O*-MeOTr ethers (E-Hex and E-Oct).

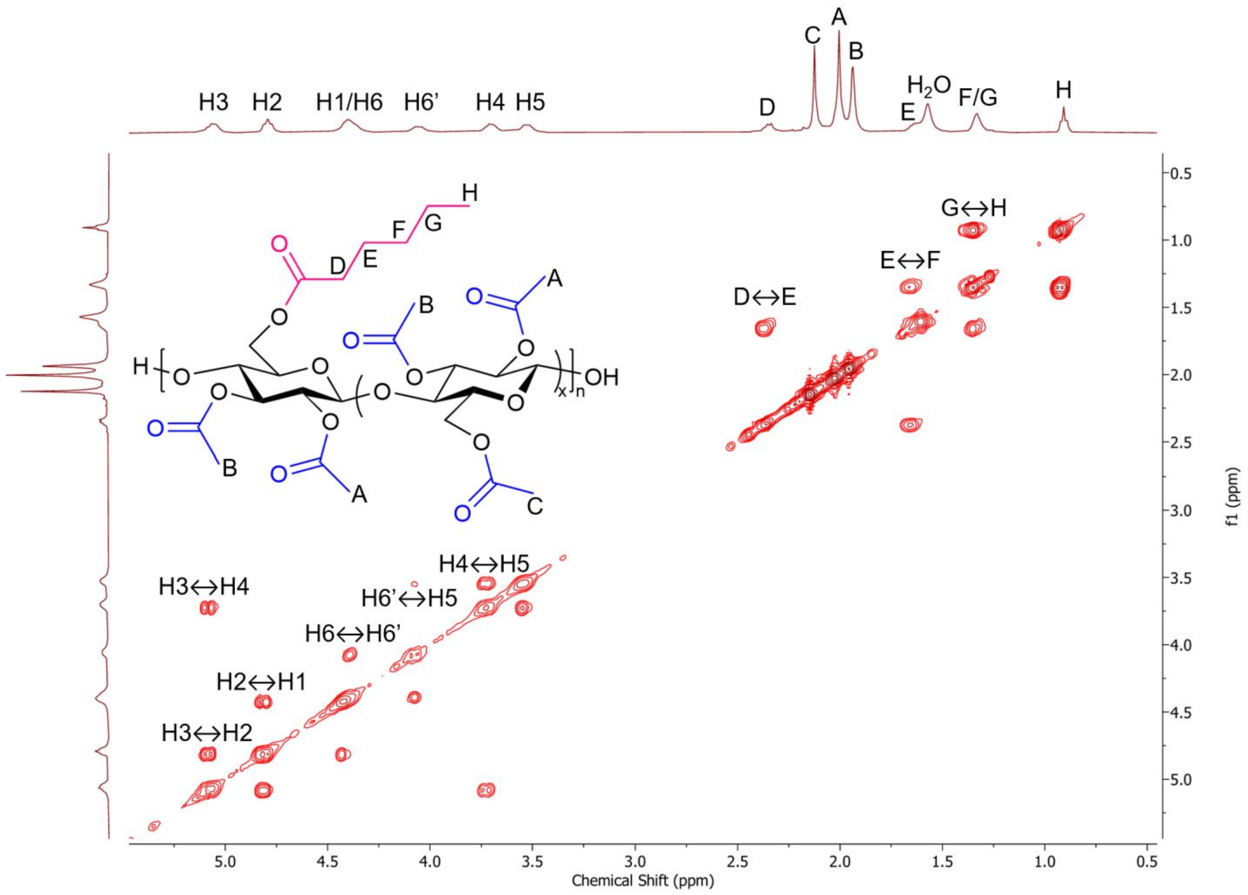

Figure S42: COSY spectrum of 2,3Ac-6Hex cellulose DS(Ac) 2.61 (E-Hex, $CDCl_3$).

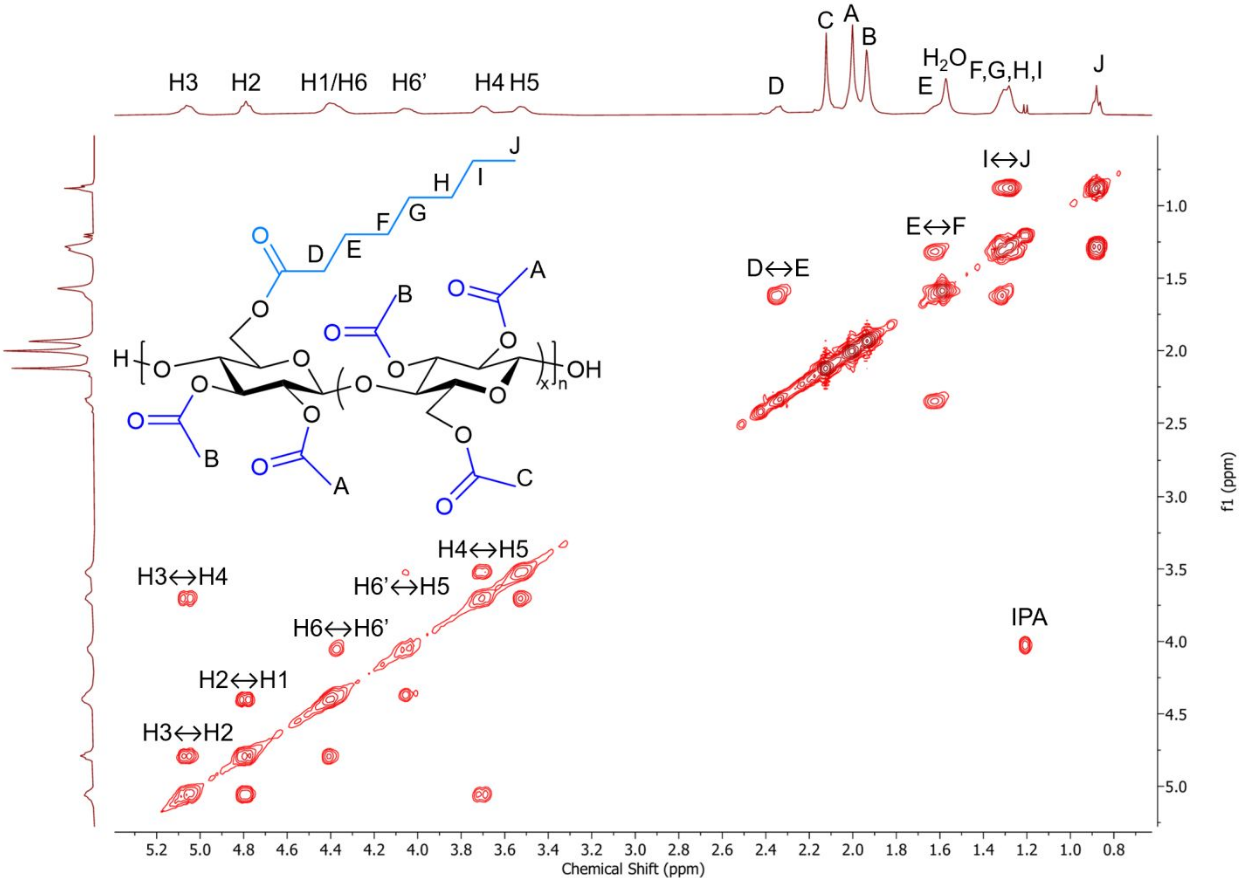

Figure S43: COSY spectrum of 2,3Ac-6Oct cellulose DS(Ac) 2.61 (E-Oct, $CDCl_3$).

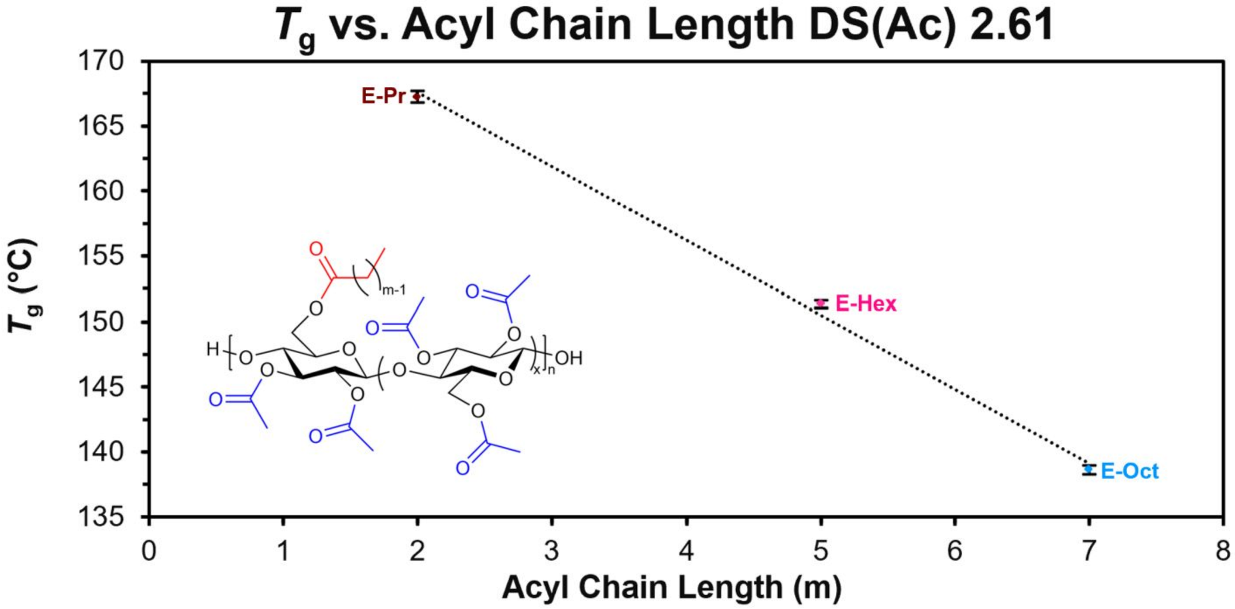

Figure S44: Plot of T_g versus acyl chain length for 2,3Ac-6Pr, 2,3Ac-6Hex, and 2,3Ac-6Oct cellulose esters DS(Ac) 2.61.

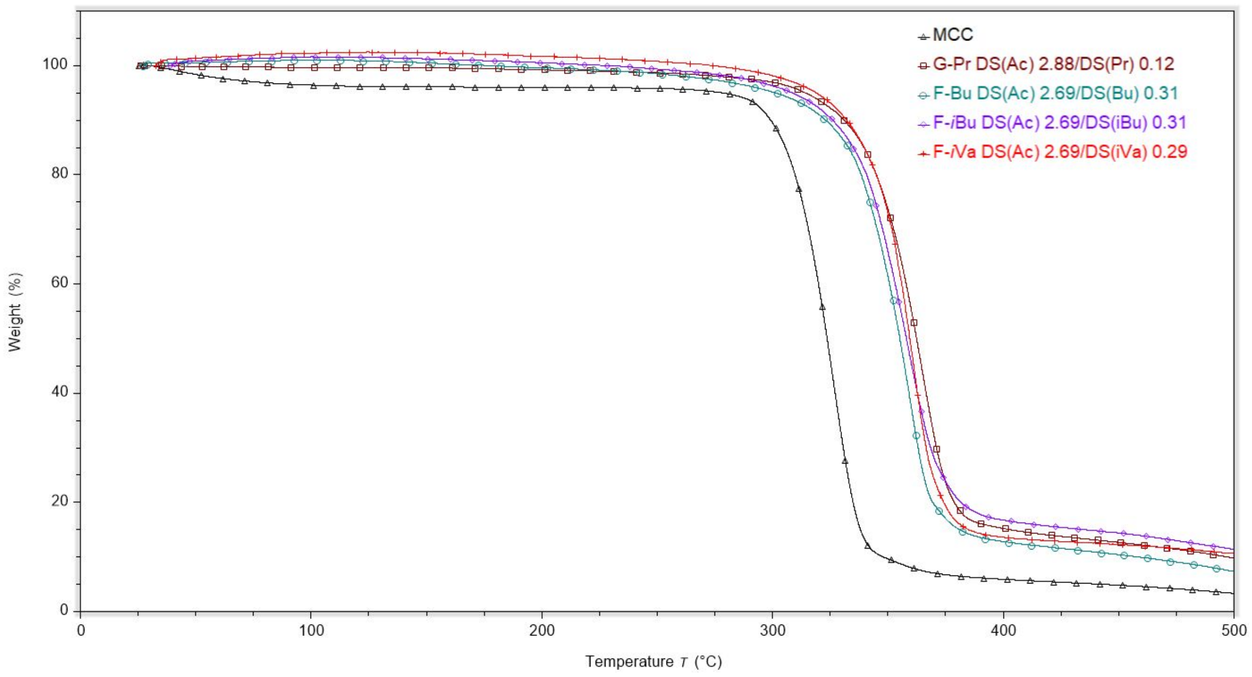

Figure S45: Representative TGA thermograms of MCC and regioselectively substituted mixed cellulose esters G-Pr, F-Bu, F-*i*Bu, and F-*i*Va.

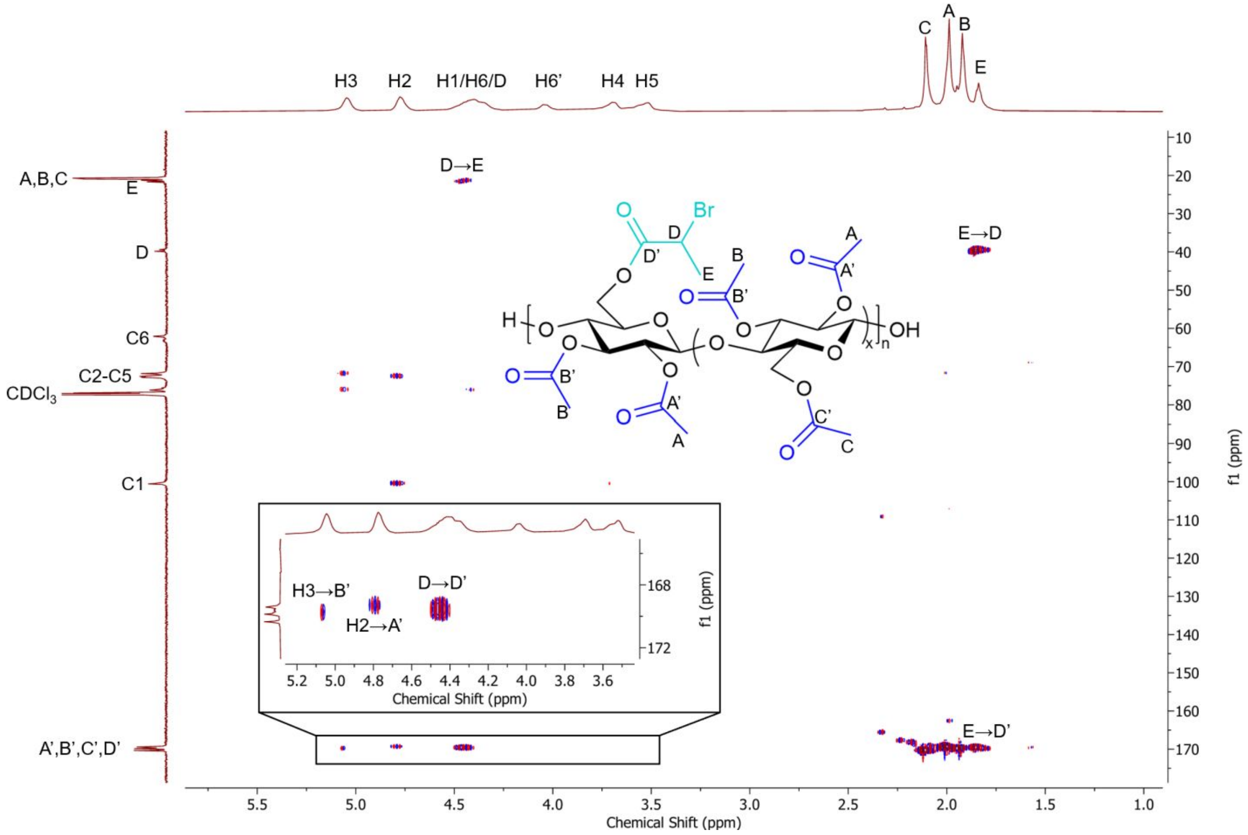

Figure S46: HMBC spectrum of 2,3Ac-6BPr cellulose DS(Ac) 2.69 (F-BPr).

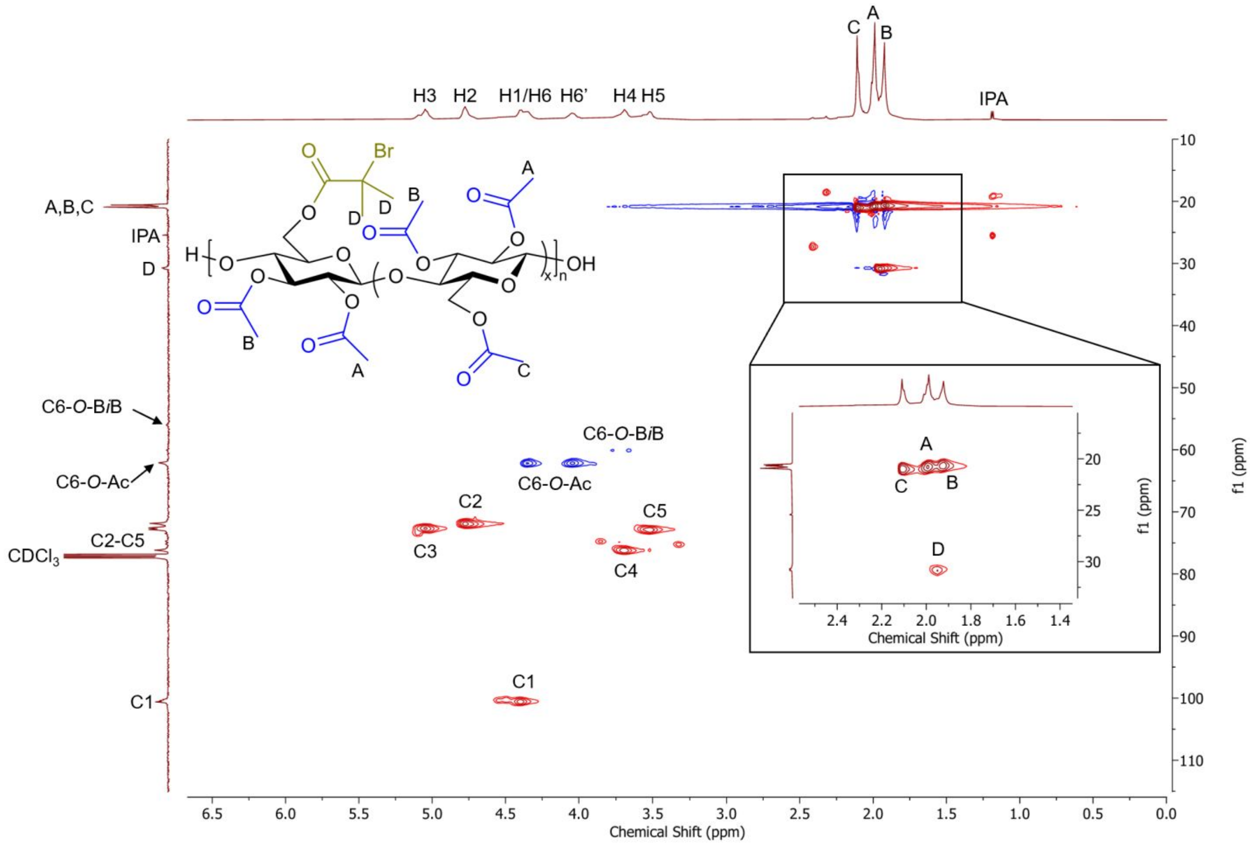

Figure S47: HSQC spectrum of 2,3Ac-6B*i*B cellulose DS(Ac) 2.69 (F-B*i*B).

References

1. Evans, R.; Wearn, R. H.; Wallis, A. F. A. Molecular Weight Distribution of Cellulose as its Tricarbanilate by High Performance Size Exclusion Chromatography. *J. Appl. Polym. Sci.*, **1989**, *37*, 3291-3303.
